# The public health impact of malaria vaccine RTS,S in malaria endemic Africa: country-specific predictions using 18 month follow-up Phase III data and simulation models

**DOI:** 10.1186/s12916-015-0408-2

**Published:** 2015-07-29

**Authors:** Melissa A Penny, Katya Galactionova, Michael Tarantino, Marcel Tanner, Thomas A Smith

**Affiliations:** Department of Epidemiology and Public Health, Swiss Tropical and Public Health Institute, Basel, Switzerland; University of Basel, Basel, Switzerland

**Keywords:** Malaria, Vaccine, Simulation, Public health impact

## Abstract

**Background:**

The RTS,S/AS01 malaria vaccine candidate recently completed Phase III trials in 11 African sites. Recommendations for its deployment will partly depend on predictions of public health impact in endemic countries. Previous predictions of these used only limited information on underlying vaccine properties and have not considered country-specific contextual data.

**Methods:**

Each Phase III trial cohort was simulated explicitly using an ensemble of individual-based stochastic models, and many hypothetical vaccine profiles. The true profile was estimated by Bayesian fitting of these models to the site- and time-specific incidence of clinical malaria in both trial arms over 18 months of follow-up. Health impacts of implementation via two vaccine schedules in 43 endemic sub-Saharan African countries, using country-specific prevalence, access to care, immunisation coverage and demography data, were predicted via weighted averaging over many simulations.

**Results:**

The efficacy against infection of three doses of vaccine was initially approximately 65 % (when immunising 6–12 week old infants) and 80 % (children 5–17 months old), with a 1 year half-life (exponential decay). Either schedule will avert substantial disease, but predicted impact strongly depends on the decay rate of vaccine effects and average transmission intensity.

**Conclusions:**

For the first time Phase III site- and time-specific data were available to estimate both the underlying profile of RTS,S/AS01 and likely country-specific health impacts. Initial efficacy will probably be high, but decay rapidly. Adding RTS,S to existing control programs, assuming continuation of current levels of malaria exposure and of health system performance, will potentially avert 100–580 malaria deaths and 45,000 to 80,000 clinical episodes per 100,000 fully vaccinated children over an initial 10-year phase.

**Electronic supplementary material:**

The online version of this article (doi:10.1186/s12916-015-0408-2) contains supplementary material, which is available to authorized users.

## Background

The first malaria vaccine against *Plasmodium falciparum* to reach Phase III clinical trials, RTS,S/AS01, has demonstrated moderate levels of efficacy against both clinical and severe malaria in young children in the 18 month follow-up of Phase III trials across 11 African sites and in several Phase II trials in Africa [1–5]. Site- and time-specific data from the Phase III trial recently published [1] indicated a vaccine efficacy against clinical cases over 18 months post third dose of 46 % (95 % CI 42–50) in children 5–17 months at first vaccination and 27 % (95 % CI 20–32) in infants (6 weeks at first immunisation, 12 weeks at third dose) [1], with much higher observed efficacy at 6 months post third dose (5–17 months: 68.3 % (95 % CI 64.3–71.8), 6–12 weeks: 47.2 % (95 % CI 39.4–54.1)) indicating an initial quick decay [1]. Since the malaria burden in many countries is still high, even a vaccine whose efficacy decays quickly may be of public health benefit. A WHO policy recommendation on the implementation of RTS,S vaccination in a number of malaria endemic countries in Africa is possible earliest at the end of 2015 [6]. Quantitative predictions of the expected public health impact and cost-effectiveness for different immunisation schedules may partly inform this recommendation.

*Plasmodium falciparum* malaria is transmitted to humans through bites from infected mosquitoes and has a complex life cycle in the human host. An infected mosquito injects sporozoites into subcutaneous tissue of the host; the sporozoites then travel to the liver. Successful invasion of hepatocytes depends on the circumsporozoite protein (CSP) of the sporozoite [7]. Following replication in the liver the parasite enters the blood stream, infecting erythrocytes and multiplying. It is the erythrocytic cycle of *Plasmodium falciparum* that causes clinical disease.

The RTS,S vaccine induces antibodies in the host against CSP and thus, with high enough antibody titre, prevents liver infection and subsequent clinical malaria that would have resulted from a blood stage infection. RTS,S has been shown to be efficacious and safe [1], but as antibody titres to CSP wane so does protection against successful infection of the liver [8], and observed efficacy against clinical disease decays relatively rapidly in the trial [1]. Repeated malaria infections induce natural, but not complete, immunity in the host to many stages of the parasite life cycle, predominantly to the blood stage causing clinical disease. There is a tendency for efficacy against clinical malaria to wane more rapidly in sites where exposure is higher [1], which is to be expected, because natural immunity to blood stage parasites is more rapidly acquired by non-vaccinated individuals. Any partially protective malaria infection blocking intervention, such as RTS,S or seasonal malaria chemoprophylaxis, aimed at infants and young children will give rise to age-shifts of burden and susceptibility to infection for this reason.

A moderately efficacious, leaky vaccine such as RTS,S, that reduces probability of infection but faces a high force of infection has complicated dynamics, including effects that cannot be detected in field trials [9] and, prior to Phase IV follow-up studies, mathematical models are essential to predict long-term outcomes of vaccination programs when delivered to populations outside trial settings. Such models indicate how population-level outcomes relate to vaccine properties (efficacy and duration of protection) or to the schedule of delivery, age at vaccination, exposure and other contextual factors. Models can address the question of whether different clinical efficacies observed in different transmission settings [4] are a result of differences in the challenge or due to differences in the vaccine effect. By identifying key long-term drivers of differences in public health impact and cost-effectiveness between possible immunisation schedules, or between different health system contexts, models can also help optimise vaccination schedules.

A number of micro-simulation models of malaria in humans have been specifically designed for predicting the public health impact of interventions, including malaria vaccines [10–13]. These models take into account levels of herd immunity and long-term effects of vaccination or other infection blocking interventions, such as deferral of events to older ages, and suggest that vaccination with pre-erythrocytic vaccines such as RTS,S via the Expanded Program of Immunisation (EPI), could substantially reduce paediatric morbidity and mortality during the first decade of vaccine use. The benefits of RTS,S are likely to be highest with levels of transmission entomological inoculation rate (EIR) between 2 to 50, which corresponds to intermediate levels of transmission in our model [9, 11, 14–17]. The EIR at the start of a vaccination program is critical, irrespective of how it has come about, or of whether transmission is increasing or decreasing [18], while herd immunity is likely to be negligible [9, 11]. A probabilistic sensitivity analysis [17] indicated that the EIR distribution, decay rate of vaccine effects and model for severe disease are important drivers of uncertainty in public health impact.

Development of these models focused heavily on fitting models to field data because of the need for quantitative predictions, but only limited data were available on the actual profile of the RTS,S vaccine. Previously published results from Phase III clinical trials of RTS,S [3, 4] have been of limited value for parameterising mathematical models of public health impact. Site- and time-specific data recently published [1] for the first 18 months of follow-up of Phase III trials now make it possible to carry out comprehensive fitting of models of vaccine action and their validation.

This paper reports the use of models within the OpenMalaria platform [11] to obtain precise estimates of underlying vaccine properties given 18 months follow-up, with observations every six months, across 11 trial sites. Using a Bayesian Markov chain Monte Carlo (MCMC) approach the likely profile of the RTS,S vaccine is determined, estimating the rate of decay of efficacy in the Phase III trial, thus allowing one to project longer term trial outcomes. Investigations of validity and the consequent estimates of vaccine properties and of clinical efficacy expected in each of the trial sites for follow-up longer than 18 months are also investigated.

In addition and beyond previous analyses, country-specific estimates are made of the likely public health impact of RTS,S programs in 43 sub-Saharan African countries, with vaccine properties aligned with the latest results from Phase III trials of RTS,S resulting from the fitting analysis in this paper. The predictions are made via a weighted averaging approach over a large database of simulations that take into account country-specific context of current malaria burden, intervention coverage, demographics, and health system capacity. Several possible implementation strategies of immunising infants and children are considered. The parameterisation and prediction approach using micro-simulations provides us with uncertainty estimates around both the vaccine profile and the predictions of public health impact, highlighting where additional data are needed. Although a very large number of computationally expensive simulations are needed, the method will allow the estimates to be updated once final Phase III data are available, without re-running these simulations.

The predictions for both public health impact and for clinical trial efficacy fitting make use of an ensemble of structurally different models [11], each a variant on a single baseline model [10], with final results derived by aggregating many simulation runs. Further examination of public health impact includes analysis of result sensitivity to vaccine properties (initial efficacy against infection, vaccine half-life of efficacy against infection, decay shape) and country-specific properties (transmission, access to care) and considers the effects of structural and stochastic uncertainty on our predictions.

## Methods

### Simulations from an individual-based stochastic model of malaria transmission

The simulation models were built around the original micro-simulation model developed for predicting the likely impact of the RTS,S malaria vaccine [10]. This model includes components simulating infection of humans, the course of parasitaemia, pathogenesis, severe disease and mortality, and infection of mosquitoes. All these components were parameterised by fitting to available field data [10, 11].

Simulations of both the clinical trial and of the public health impact of RTS,S were made using six different model variants to represent *Plasmodium falciparum* malaria. These model variants form an ensemble from which to make predictions of the impact of RTS,S in the trials and were chosen from among a larger set of model variants [11] because they represent the diversity of available variants that fit well to the calibration datasets. They are described in brief in Table 1.

**Table 1:** Summary of Simulations: Variables and levels

Variable	Levels simulated
Vaccination age for fitting databases	EPI cohort 6, 10, 14 weeks
	5-17 month cohort all children ages between 5-17 months first dose, and for third dose 8-20 months
Vaccination age in predictions databases	EPI schedule 6, 10, 14 weeks
	Extended routine vaccination schedule : 6, 7.5 and 9 months
	Boosting is 18 months post third dose (EPI 21 months, extended routine 27 months)
Model variants [11]	1) R0000 Base model
	2) R0068 Heterogeneity in transmission: within-host variability
	3) R0131 Immunity decay in effective cumulative exposure
	4) R0132 Immunity decay in immune proxies
	5) R0133 Immunity decay in both immune proxies and effective cumulative exposure
	6) R0670 Heterogeneity in susceptibility to co-morbidity
EIR	0.1^*a*^,1,2,4,8,16,64,256
Access to uncomplicated case management ^*b*^ (%)	0,5,40
Access inpatient care for severe cases ^*c*^ (%)	0,100 (for fitting 0,40,80)
Vaccination coverage ^*d*^ (%)	0,100
Initial efficacy against infection (%)	30,50,65,80,95 (for fitting 20,30,40,50,60,85)
Half-life (years)	1,2,5
Weibull decay shape parameter (*k*)	*k*=1 (exponential)
	*k*=0.5 (bi-phasic)
	*k*=2 (slow decay, followed by quick decay)

^*a*^EIR of 0.1 was not simulated, but any predictions for this level are taken as 10% of EIR 1

^*b*^Probability of access to treatment for uncomplicated disease during a 5-day period

^*c*^Probability of access to hospital care (or equivalent) for severe disease during any 5-day period

^*d*^For each of the four delivery schedules

### Predictions via weighted ensemble predictions

For both prediction of clinical trial outcomes and country-specific predictions of impact, weighted averages of a large number of simulations were calculated with a wide range of vaccine characteristics, deployed across a range of health system and transmission settings. The weights applied to each simulation are dependent on the country-specific data and on vaccine properties being investigated for predictions or fitting.

For fitting to trial data, two databases of simulations that predict the effect of vaccination of two vaccine cohorts, EPI and 5–17 months in the Phase III trials, were created. Each simulated the trials explicitly as a complete factorial combination of all levels of each variable listed in Table 1. This resulted in a total of 311,040 simulations of vaccination (coverage 100 %, at 6–12 weeks or 5–17 months) and 4,320 comparator simulations (coverage 0), with the variables and levels being six structurally different models: eight different levels of EIR, three different levels of access for uncomplicated disease, and three different levels of access for severe disease. In addition, vaccine characteristics considered were: initial efficacy against infection (six levels 20 %–85 %), the half-life of decay of efficacy against infection over time (three levels 1–5 years), and decay shape (four levels, corresponding to exponential and three Weibull decay functions) as well as vaccination coverage (summarised in Table 1). Additionally, for every simulation combination (referred to as a scenario) results from multiple seeds were recorded to estimate the stochastic uncertainty in the predictions. Results for an EIR of 0.1 were not simulated but calculated by linear interpolation between the comparators and the results for EIR 1 (as done previously [19]).

For country-specific prediction four sets of OpenMalaria simulations were created, one each for each of the four immunisation schedules considered for RTS,S delivery, each comprising a full factorial design covering the entire span of vaccine properties, health system-specific parameters, vaccine schedule coverage and transmission-specific parameters for each of the six model variants (see Table 1). Overall this required a total of 226,800 simulations of vaccination (coverage 100 %), and 1,260 comparator simulations (coverage 0 %).

Each scenario tracked a population with size 100,000, and a model burn-in period of 99 years was completed (to achieve a periodic stable state) before vaccination was initiated. For the public health impact predictions, events and population demographics were recorded with yearly surveys for 20 years from the start of vaccination campaign. Simulated surveys for the clinical trial simulations were carried out at 6 month intervals. At each survey and for each age group the following was monitored: the prevalence of patent parasitaemia, the numbers of uncomplicated cases, severe cases, direct malaria deaths, indirect malaria deaths, sequelae events, first line, second line and third line treatments given, hospitalised cases that recovered, hospitalised cases that resulted in sequelae and hospitalised cases that resulted in death.

To obtain impact predictions of a given vaccine delivery schedule or vaccinated cohort for a given country or trial site and vaccine profile, for a certain outcome, weighted averages over all simulations in the appropriate database were used (see Additional file 1: Methods). Results are presented as mean weighted averages and reported range via minimum and maximum limits over the weighted averages for all models and seeds, without model weighting. This captures both structural and stochastic uncertainty in the model.

### Pre-erythrocytic vaccine efficacy and decay

The action of a pre-erythrocytic vaccine like RTS,S is implemented in the models as vaccine efficacy in preventing a new infection. This corresponds to the proportion of blood stage infections averted, and hence is similar to the efficacy measured in a sporozoite challenge trial. This is different from the efficacy in averting clinical episodes as reported in the Phase III clinical trials, which differs from the simulated efficacy both in average value and in the way in which it evolves over time, with factors including transmission heterogeneity and age-shifting of susceptibility leading to greater decays over time in field-measurable quantities than in the underlying efficacy against infection assumed in the models [11].

OpenMalaria allows different rates of decay [20] in underlying efficacy over time and different shapes of the decay. For fitting, decay was assumed to follow a Weibull decay curve described by the initial value of the efficacy, the half-life, and a shape parameter, *k*. For further details see Additional file 1: Methods. Simulations were carried out with shape parameter *k* with values of 0.5,1 or 4, where *k*=1 corresponds to exponential decay. For *k* less than 1, the initial decay is faster than exponential and then slower than exponential after the time equivalent to half-life is reached; this is similar to a bi-phasic like decay, with a sharp decline (quick decay) in efficacy followed by longer decay. For *k* greater than 1, the initial decay of efficacy against infection is slow until the time equivalent to half-life, and then the decay is much faster.

### Determining vaccine properties from Phase III clinical trial data

#### Simulations of RTS,S Phase III clinical trials with OpenMalaria

The vaccination cohorts 6–12 weeks and 5–17 months were explicitly simulated according to the trial design [1]. The cohorts were as follows: 1) For the 6–12 week cohort, the vaccinated cohort was constructed by vaccinating all simulated individuals for a year when they reached age 3 months (assuming that at that point they received the third dose and achieved maximum efficacy against infection). These individuals were followed for 6 monthly intervals after their third dose to replicate trial reported events (this accounted for seasonality in exposure). The control cohort was not vaccinated, but the same ages were followed as the vaccinated cohort (illustrated in Additional file 1: Figure SM1a). 2) For the 5–17 month simulation design, individuals aged 5–17 months at day one of simulation were enrolled into two equally sized cohorts. The vaccinated cohort was vaccinated at day one after warm-up assuming different levels of initial vaccine efficacy against infection that would be achieved at third dose. Events were then counted at 6 monthly periods. The control cohort received no vaccination.

Additional file 1: Figure SM1a details the cohorts and how events averted are calculated for the virtual cohorts.

#### Fitting of vaccine properties

The underlying vaccine properties were fitted to site-specific *according to protocol* (ATP) values of the numbers of clinical cases meeting the primary case definition in each 6 month period, in each age group in each trial site for the control cohort and for each vaccinated cohort (EPI and 5–17 months). Due to absence of site- and time-specific data from Phase II, trial data is restricted to Phase III sites using adjuvant AS01. Data from the Kilifi and Manhica trial sites were used for a preliminary validation and thus not used for the fitting of vaccine properties. All data was published in [1], with the study conducted in accordance with Good Clinical Practice guidelines, and in compliance with the Helsinki Declaration. The trial protocol was approved by the ethical review board at each study centre and partner institution and by the national regulatory authority in each country (detailed in Additional file 2: Table S1A of the clinical trial publication [1]). As this work involves data stimulations and analysis, informed consent was not required.

A Bayesian MCMC approach was used to estimate vaccine properties, site-specific access to care, and the extent of within-site variation in clinical disease (number of episodes per individual for a defined time period). This approach results in a posterior distribution for unknown parameters. The log of the observed clinical data (disease rates in the control and vaccinated groups at each time point) was assumed to be normally distributed with the log of the model predictions for a given set of parameters. Namely,

(1) $$ \log(Y_{t,i})|\theta,\sigma_{i} \sim \text{Normal} \left(\log(\hat{\mu}_{t,i}(\theta)),\sigma_{i} \right),  $$

where *Y*_*t*,*i*_ is the observed disease rate (for control or vaccinated) at time *t* and site *i*, $\hat {\mu }_{y,i}$ is the weighted model prediction for the equivalent outcome at time *t* and site *i*, *θ* represents the parameters being fitted (vaccine properties and access to care), *σ*_*i*_ is the standard deviation for trial site *i*. The weighted model prediction, $\hat {\mu }_{t,i}$, is a predicted weighted estimate for the disease rate at time *t* and site *i* detailed below and uses two databases of cohort predictions from OpenMalaria, the trial site-specific inputs concerning transmission and the MCMC sampled parameters *θ* to calculate the weights for efficacy, half-life and access.

A series of different models were fitted (increasing in complexity and varying as to which parameters to fit or assume, or whether to parameterise against one cohort (6–12 weeks or 5–17 months) or both simultaneously) as listed in Additional file 2: Table S1. The models fit were:

1. Fit cohort-specific efficacy, fit site-specific access, fit common variance in incidence across sites and assume vaccine half-life (either 1 year or 3 years)
2. Fit cohort-specific efficacy, fit site-specific access, fit site-specific variance in incidence and assume vaccine half-life (either 1 year or 3 years)
3. Fit cohort-specific efficacy, fit vaccine half-life, fit site-specific access, fit common variance in incidence across sites
4. Fit cohort-specific efficacy, fit vaccine half-life, fit site-specific access, fit site-specific variance in incidence

For models fitted simultaneously to data of both vaccinated cohorts, separate vaccine initial efficacies against infection were fitted for each cohort, but common half-lives of decay of efficacy against infection, access to care and levels of within-site variation in incidence were used. In addition, site-specific parameters were estimated for the average exposure to infective mosquitoes (EIR) and the proportion of uncomplicated malaria fevers accessing care, by simultaneous fitting to the parasite prevalence, and the recorded clinical incidence data from the control arms. For each site, within-site variability in EIR was allowed by defining a limited number of EIR bins. For any specific EIR, an estimate of the proportion of the site population exposed at that level was used, calculated from population-weighted averages of the pixel-specific posterior distributions corresponding to that bin, derived from the Malaria Atlas Project (MAP) 2010 prevalence surfaces [21], as described in Additional file 1: Methods and [Penny et al: Distributions of malaria exposure in endemic countries in Africa considering country levels of effective treatment, submitted].

The standard statistical criteria (using the deviance information criterion (DIC)) were calculated and used to compare the different fitted models and determine the most appropriate model for final vaccine parameters (see Additional file 1: Methods).

In each case, the response to which the models were fitted was the number of episodes recorded in the health facilities divided by those at risk (as opposed to the total number of clinical cases uncomplicated and severe, making no assumption on case definition in the trials), which was assumed to correspond to the number of malaria treatments recorded in the simulations divided by the number of individuals at risk.

Two chains with very different initial conditions for efficacy, access to care and half-life were used for each fit. Uniform non-informative priors were assigned for all parameters. Posterior distributions were sampled for each of the fitted parameters (EPI efficacy against infection, 5–17 months efficacy against infection, vaccine half-life, within site variation against clinical disease and site-specific access to care).

### Country-specific predictions of the expected public health impact of RTS,S

Conditional on the vaccine properties informed by fitting to Phase III data, predictions of the likely public health impact of RTS,S when deployed in 43 sub-Saharan Africa malaria endemic countries via four vaccination schedules was made. Multiple doses of RTS,S are required to give modest protection against clinical episodes and to induce high antibody titres. A 3-dose regimen of vaccination was considered and given via the Expanded Program on Immunisation (EPI) with a standard diphtheria-tetanus-pertussis (DTP) schedule of 3 doses between 6 and 12 weeks of age. In addition, an extended routine schedule beginning with the vitamin A visit at 6 months and subsequent doses at 7.5 months and ending with measles-containing vaccine at 9 months is examined (this schedule is considered as a possible implementation of the 5 to 17 months cohort in the Phase III trials [22] which demonstrated higher clinical efficacy compared to the 6–12 week cohort in trial data [4, 22]). The addition of a booster at 18 months after the third dose to both routine EPI and the extended routine (6–9 months) was also considered. The likely efficacy of the RTS,S booster dose has not yet been demonstrated, and in the absence of Phase III data the initial efficacy against infection and decay of the booster dose was assumed to be the same as that of the third dose.

#### Vaccine properties and weights

The vaccine property of initial efficacy against infection, half-life and decay shape and subsequent weights used in the weighted averages (see Additional file 1: Methods) for each delivery are calculated to give initial efficacies, half-life and decay shape as determined via fitting to Phase III trials (see Table 2).

**Table 2:** Fitted vaccine RTS,S properties

Cohorts	Initial efficacy	Half-life (exponential decay)
6-12 week	62.7 %	1.12 years
cohort	(with 95 % CI 39.5-80.3 %)	(with 95 % CI 1-1.43 years)
5-17 month	79.2 %	1.12 years
cohort	(with 95 % CI 67.3-84.8 %)	(with 95 % CI 1-1.43 years)

#### Country-specific malaria transmission, health system and vaccination coverage

Country-level realistic distributions of malaria exposure, access to case management for malaria treatment and inpatient care, demographics and vaccination coverage have been collated and modelled. Further details are provided in Additional file 1: Methods.

Vaccine introduction was assumed to occur at the beginning of 2017 for all countries and the country-specific immunisation coverage levels for RTS,S delivered via routine EPI based on the third dose of DTP reported by WHO-UNICEF for EPI in 2012 [23]. For simplicity and to avoid erroneous assumptions, instantaneous coverage of RTS,S vaccination is assumed at 2017 (at 2012 DTP3 levels) and these remain constant from 2017 up to 2032. DTP3 coverage levels were scaled by 75 % for extended routine (6–9 months) delivery. In addition, boosting schedules for EPI and extended routine assume 80 % coverage of the third dose for that schedule. It is noted that these coverage values are controversial [24] and that the WHO-UNICEF values for EPI may be slightly optimistic. An overestimation of the coverage achieved will lead to overestimation of the public health impact of the vaccine program.

The level of malaria transmission (distributions of EIR) for a particular country was estimated based on the MAP 2010 prevalence surfaces [21] for the geographic area in question. Similar to the trial sites for fitting, for this method MAP prevalence and the OpenMalaria model relationship between EIR and prevalence, along with country-specific access to effective treatment, were used to derive distributions of exposure [Penny et al: Distributions of malaria exposure in endemic countries in Africa considering country levels of effective treatment, submitted]. Country-level estimates of access to malaria treatment for uncomplicated cases are detailed in Additional file 1: Methods and [25]. The derived distributions of malaria transmission for each country reflect transmission at the current level of control interventions.

#### Public health impact outcomes

The numbers of malaria infections, uncomplicated malaria episodes, severe malaria episodes, malaria-related hospitalisations, and direct and indirect malaria deaths for each country by time were simulated, both in the absence of the vaccination and in the presence of the RTS,S program (illustrated in Additional file 1: Figure SM1b). Public health impact was calculated as events averted in each country in time (or cumulative in time), events averted (or cumulative) per 100,000 fully vaccinated individuals and cumulative effectiveness for a given outcome. The events averted comprise numbers of uncomplicated episodes, severe episodes, hospitalisations, direct malaria deaths, all deaths (direct malaria deaths and indirect associated with co-morbidities) and unweighted and undiscounted disability adjusted life years (DALYs) averted. Analogous algorithms were used to compile the numbers of events averted for each of these outcomes. Details of DALY calculations and how the public health impact was calculated over weighted averages are detailed in Additional file 1. Indirect malaria deaths are deaths that occur because of malaria infection but that do not satisfy the definition of direct malaria deaths. These comprise neonatal deaths secondary to malaria in pregnancy, and deaths resulting from interactions between pathogens where malaria plays an essential role, but the terminal illness does not satisfy the definition of severe malaria [26].

#### Sensitivity analysis

Both the robustness and sensitivity of the country-specific predictions of public health impact of RTS,S with respect to vaccine parameter uncertainty and country-specific implementation, transmission, and health system parameters were assessed. Ranges of public health impact predictions are produced by varying a single input while keeping all other parameters at their reference value (see Table 3 and Additional file 1: Table SM1). Uncertainty about vaccine properties will have the greatest impact on the level of predictions. Ranges for vaccine properties are based on posteriors from the fitting to Phase III data assumed; other ranges associated with country-specific inputs are illustrative of a given country setting but broad enough to inform our understanding of the direction and magnitude of the potential bias in impact estimates induced by uncertainty around these key parameters (Additional file 1: Table SM1).

**Table 3:** Sensitivity analysis and reference levels of inputs

Name	Initial efficacy ^*a*^	Half-life ^*a*^	Decay shape ^*a*^	Vaccine coverage
Reference	EPI: 62.5 %, 6-9 months: 79.2 %	EPI : 1.12 year, 6-9 months: 1.12 year	Exponential	EPI: DTP3, 6-9 months: 75 % of DTP3
B	Reference	Reference	Reference	Increase 6-9 months to EPI coverage
C	Increase (EPI: 80.3 %, 6-9 months: 84.8 %)	Reference	Reference	Reference
D	Decrease (EPI: 39.5 %, 6-9 months: 67.3. %)	Reference	Reference	Reference
E	Reference	Increase (EPI : 3 year, 6-9 months: 3 year)	Reference	Reference
F	Decrease (EPI: 39.5 %, 6-9 months: 67.3. %)	Increase (EPI : 3 year, 6-9 months: 3 year)	Reference	Reference
G	Increase (EPI: 80.3 %, 6-9 months: 84.8 %)	Increase (EPI : 3 year, 6-9 months: 3 year)	Reference	Reference

^*a*^Vaccine efficacy against infection and vaccine half-life of decay against infection

## Results

### Methodological advances

The weighted ensembles approach using large databases of predictions has enabled both a novel methodology for parameterising underlying vaccine properties of RTS,S and a means to update those parameters quickly as new clinical efficacy data from the Phase III trial is available. In addition, using databases of predictions of implementing the vaccine in populations via four possible immunisation schedules, the weighting methodology allows quick estimation of the expected public health impact for most up-to-date vaccine properties. The results presented here are based on the 18-month follow-up of the RTS,S Phase III trials.

### RTS,S vaccine properties determined from Phase III clinical trial data

**Fig. 1:**
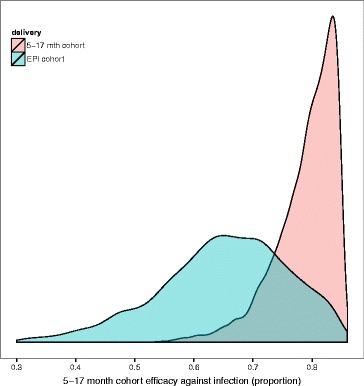
Posterior distributions of initial efficacy against infection for 5–17 month and EPI cohort for best fitted model. Posterior distributions of efficacy against infection for the 5–17 month cohort and EPI cohort for models fitted with the adjusted transmission assumptions (ii). Results are from final model fit, fitting vaccine properties initial efficacy, half-life against infection for exponential decay, site-specific access to effective treatment, and site-specific variation in incidence. The distribution is shown for efficacy when fitting for both cohorts; rose colour indicates the 5–17 month cohort and blue the EPI cohort

**Fig. 2:**
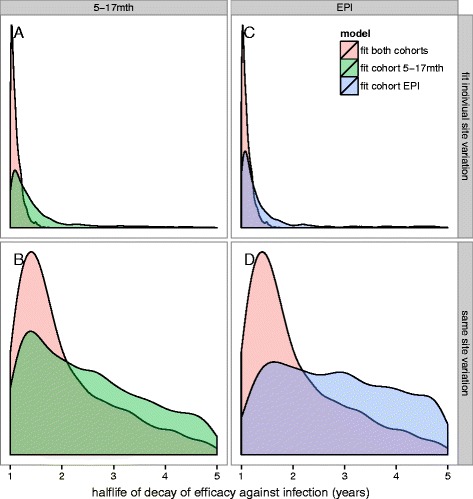
Posterior distributions of half-life of decay of efficacy against infection. Posterior distributions of half-life of decay of efficacy against infection for models fitted with the adjusted transmission assumptions (ii), assuming exponential decay. Panels **a** and **c** show fits when site-specific variation in incidence is fitted. Panels **b** and **d** show fits when common variation in incidence across all sites is fitted. The green histograms indicate when 5–17 month cohort is fitted alone, the blue the EPI cohort and the rose when both cohorts fit

Results of the Bayesian MCMC fits to data from the 18 month follow-up Phase III data are summarised in Additional file 2: Table S1. The table shows estimated posterior distributions (mean and 95 % confidence interval) for vaccine properties (efficacy against infection for EPI and 5–17 month cohort, vaccine half-life) for each of the fitted models for transmission assumption (ii) of Additional file 1: Methods, assuming exponential decay. Plots of the posterior distributions are shown in Figs. 1 and 2 and Additional file 2: Figures S1-S2 and Figures S4-S5. Statistical models were fitted either jointly to both trial cohorts or individually to single cohorts. Model diagnostic estimates of deviance and deviance information criteria (DIC) are also summarised in Additional file 2: Table S1. Results and differences between the models are given in Additional file 2: Results.

In general, when fitting to both cohorts simultaneously, or to the 6–12 week or 5–17 month cohort separately (Additional file 2: Table S1) the best fitting models with lowest DIC were obtained when models fit for site-specific variation in incidence, even though these estimates had the same values for all sites. In addition, best fits were obtained by either assuming a vaccine half-life of 1 year or when fitting for vaccinehalf-life.

Our optimum model fit, with lowest DIC and narrowest posterior distributions for half-life and efficacy (model 18), estimated vaccine properties as follows (mean, 95 % confidence intervals):

1. Initial vaccine efficacy against infection in the 6–12 week cohort : 62.7 % (39.5–80.3 %)
2. Initial vaccine efficacy against infection in the 5–17 month cohort : 79.2 % (67.3–84.8 %)
3. Half-life for decay of efficacy against infection with exponential decay of 1.12 years (with 95 % CI 1–1.43 years)

All the fitted models had estimates of the half-life of vaccine efficacy against infection of approximately 1 year. This estimate does not depend on the linear interpolation between simulated scenarios since 1 year is among the values of half-life simulated (Table 1). Lower DIC was obtained for models fitting for site-specific variation in incidence, assuming a half-life of 1 year or fitting for half-life, indicating that under the assumption of exponential decay, and with only 18 months of follow-up data, the half-life against vaccine efficacy against infection is likely to be around 1 year, rather than longer (Fig. 2 and Additional file 2: Table S1).

For the EPI cohort, the predicted mean initial efficacy against infection is lower than that of the 5–17 month cohort, and the posterior distributions on efficacy against infection (see Additional file 2: Figure S2) are much wider than those predicted for the 5–17 month cohort. Narrower distributions are obtained when one fits for a common variation in incidence across all trial sites. Not surprisingly, much lower mean initial efficacy is predicted for models assuming a half-life of three years.

The predicted posterior distributions for half-life of decay (Fig. 2) are similar for both cohorts, with lower half-life obtained when both cohorts are fitted jointly. Overall the posterior densities for both EPI efficacy and 5–17 month efficacy and for the corresponding half-life of efficacy are narrower when the model is fitted jointly to both cohorts (Fig. 2), rather than when separate models are fitted. This is also not surprising as there is more data informing site-specific parameters (that is, access to care). When the model includes site-specific terms to model the variance in incidence, the half-life posteriors are much narrower and more precise.

Models attempting to fit for the decay shape parameter of the non-exponential Weibull decays failed to converge, because more time points are needed to simultaneously estimate the effects of other factors and the shape of vaccine efficacy decay.

#### Access to care and site-specific variation in clinical incidence

Estimated site-specific access to care for our optimal model (model 18) indicates that access to effective treatment is low for most trial sites (Additional file 2: Figure S4 shows posterior distributions for access to effective treatment for model 18 (fit for half-life, efficacy, site-specific variation in incidence with adjusted transmission assumptions (ii) for both cohorts)), though it is still higher than the average levels for the countries in which the trials took place [25].

Site-specific variation in estimated incidence varied widely between sites when prevalence was low (Additional file 2: Figure S5). Estimating site-specific variation, as opposed to a common variation in incidence, resulted in better fitting models, and narrow posterior distributions of vaccine properties initial efficacy and half-life, indicating that the variation within each site is perhaps more important than the variation between sites.

#### Comparison of different model predictions with site-specific data and validation

Plots of trial site clinical efficacy by 6 month periods from the 18 month follow-up of Phase III [1], along with predicted mean and credible intervals for different fitted models are shown in Additional file 2: Figure S3 for the 5–17 month cohort and for the EPI cohort. Further plots comparing predicted incidence with observed (Additional file 2: Figure S6 and Additional file 2: Figure S7) are discussed in Additional file 2: Results. Further comparisons to incidence are detailed in Additional file 2: Figure S6 and Additional file 2: Figure S7.

In general, the model predictions for clinical efficacy for both the EPI and 5–17 month cohorts captured the observed trends in the trials [1], and predictions fall within the data confidence limits. For some sites large confidence bounds were obtained on the predicted disease rate per person year and clinical efficacy, indicating difficulties in achieving convergence for those sites with low transmission or less than three observed time points. An exception was the first 6 month time point efficacy for the 5–17 month cohort, to which the fit was rather poor, possibly related to maternal immunity in the model [27].

Two sites, Manhica and Kilifi, were not used in the fitting, but predicted clinical efficacy is shown in Additional file 2: Figure S8 for the 5–17 month and EPI cohorts. There are reported wide confidence bounds for both sites and outliers with estimates of clinical efficacy less than 0, and thus limited data to validate the model with any certainty. The validation thus provided no reason to reject the new parameterisations, but had only very limited statistical power.

#### Predicted clinical efficacy beyond 18 months

**Fig. 3:**
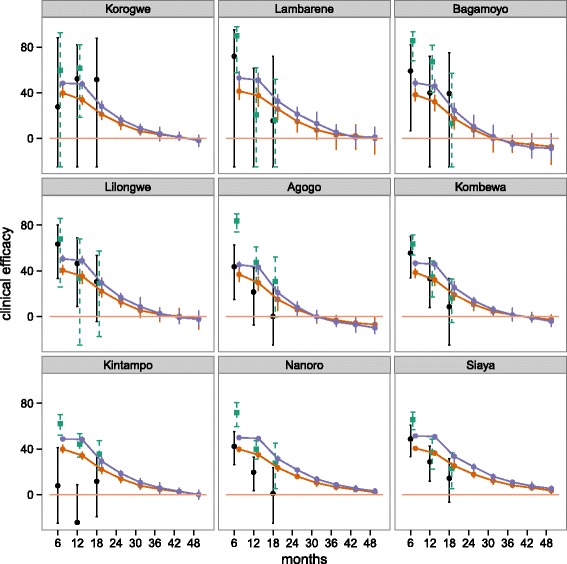
Predicted clinical efficacy beyond 18 months for EPI and 5–17 month cohorts for trials sites used for the fitting. Projections of clinical efficacy by site for the EPI cohort (orange) and 5–17 month cohort for follow-up longer than 18 months for trials sites used for fitting. Predictions are results of assuming vaccine parameters from model 18 (fit for half-life, site-specific variation and to both cohorts) and assuming site levels of exposure from adjusted transmission assumptions (ii). Black indicates mean estimates of trial data with 95 % CI for the EPI cohort and green for 5–17 month cohort, orange the model predictions for EPI cohort and purple model predictions for the 5–17 month cohort

Predictions of expected clinical efficacy by 6 month time points in each of the trial sites for 6, 12 and 18 month follow-up and for longer follow-up than 18 months are shown in Fig. 3 for both the 5–17 month and EPI cohorts. These results assume exponential decay with vaccine properties from fitted model 18 (Additional file 2: Table S1). Results show that we expect efficacy below zero, with a small rebound, at around 3–3.5 years for some trial sites. This is consistent with Phase II follow-up [22]. The clinical efficacy of both cohorts is predicted to converge around 3 years after last vaccine dose.

**Fig. 4:**
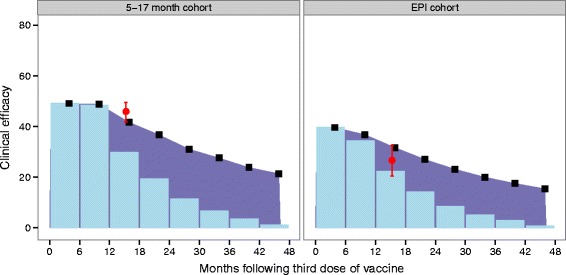
Clinical efficacy predicted for both 6 month periods and cumulative predicted clinical efficacy in time. Predicted estimates of clinical efficacy at each 6 month follow-up, and cumulatively in time for the EPI and 5–17 month cohort over all trial sites. Reported efficacy at 18 months post third dose (mean and 95 % CI) over all trial sites for each cohort is indicated by red. Prediction estimates by 6 month time periods (mean) are shown in blue bars for each cohort, 5–17 months (left) and EPI (right). Predictions for cumulative efficacy in time are shown in black, with purple shading to indicate difference between 6 month period predictions. Predictions are from the best fitted model (fit to both cohorts, fit half-life, and site-specific variation), with the adjusted transmission assumptions (ii)

Predictions of expected clinical efficacy by 6 month time points, namely the percentage of clinical events averted in the previous 6 month period, and expected cumulative efficacy in time over all trial sites for the two cohorts are shown in Fig. 4. The overall efficacy against clinical disease in time is predicted to be sustained for both the 6–12 week and 5–17 month cohorts, even up to four year follow-up. However, the prediction of efficacy against clinical cases (including repeated episodes in the same individuals) for 6 month time intervals indicates that the proportion of cases averted in each 6 month period will decrease to 10 % towards the end of the final follow-up of the trial.

### Predictions of the public health impact of RTS,S implementation in endemic malaria countries from 2017

The values for each of the factors driving country-specific predictions of public health impact are tabulated in Additional file 1: Table SM2. These include country demographics (total population and surviving infants), underlying transmission profiles, access to effective treatment and vaccination coverage. Overall, the predicted non-vaccine burden from the models suggest that there are somewhat more malaria episodes and deaths attributable to malaria than are estimated by WHO [Penny et al: Distributions of malaria exposure in endemic countries in Africa considering country levels of effective treatment, submitted], though these estimates vary considerably between countries.

A substantial number of clinical events are predicted to be averted 10 years following introduction (total over endemic countries in Table 4 or per fully vaccinated individuals in Table 5). Under the immunisation schedules of targeting only the young, and considering that the protection from the vaccine wanes relatively rapidly, this translates into a relatively low proportion of malaria events averted over the entire population (range from 1–4 % for clinical events and up to 10 % for deaths (Additional file 2: Figures S14-S15), depending on immunisation schedule). These low proportions are to be expected since malaria disease can occur at any age, but only the youngest cohorts will be targeted by vaccine. The proportion of events averted for persons under five is much higher.

**Table 4:** Cumulative total events averted (all ages) across 43 sub-Saharan African countries, cumulative by 5 year periods for each of the four deliveries: EPI (6–12 weeks), EPI with boosters, expanded routine (6–9 months) and expanded routine with booster

Events averted	At 5 year followup (thousands)	At 10 year followup (thousands)	At 15 year followup (thousands)
EPI (6-12 weeks)			
Uncomplicated	72,723 (47,523-82,667)	148,991 (101,067-176,664)	204,966 (144,404-246,843)
Severe	1,757 (1,223-2,460)	2,608 (1,159-3,943)	3,020 (446-5,414)
Hospitalisations	842 (579-1243)	1,254 (527-1,955)	1476 (220-2,653)
Direct deaths	206 (126-340)	370 (152-576)	482 (149-798)
All deaths	503 (235-647)	1033 (558-1389)	1537 (879-2074)
DALYs	27,075 (12,869-34,801)	55,687 (30,489-74,302)	83,364 (48,162-111,434)
Direct DALYs	11,482 (7,159-18,740)	20,743 (9,307-31,884)	27,642 (9,999-44,694)
EPI with booster			
Uncomplicated	97,337 (63,960-110,268)	217,564 (146,622-256,595)	304,518 (212,980-364,054)
Severe	2,441 (1,705-3,333)	4,087 (2,554-5,810)	4,975 (2,225-7,629)
Hospitalisations	1,168 (808-1,655)	1,960 (1,241-2,858)	2,416 (1,113-3,703)
Direct deaths	281 (173-421)	549 (332-823)	731 (384-1,134)
All deaths	622 (320-816)	1334 (771-1674)	1995 (1163-2580)
DALYs	33,413 (17,362-43,664)	71,818 (41,729-89,598)	108,255 (63,463-138,582)
Direct DALYs	15,519 (9,626-23,042)	30,580 (18,902-45,470)	41,714 (23,481-64,095)
Expanded routine (6-9 months)			
Uncomplicated	58,230 (38,260-66,696)	119,426 (80,366-141,899)	162,454 (112,002-195,551)
Severe	1,528 (1,054-2,054)	2,560 (1,788-3,490)	3,239 (1,974-4,611)
Hospitalisations	727 (501-1,048)	1224 (836-1,746)	1,565 (890-2,315)
Direct deaths	162 (88-264)	306 (173-488)	406 (217-652)
All deaths	329 (160-448)	684 (375-886)	1008 (500-1293)
DALYs	17,670 (8,749-24,049)	36,881 (20,354-47,826)	54,822 (27,867-70,146)
Direct DALYs	8,955 (4,999-14,537)	17,123 (9,960-26,781)	23,251 (13,386-36,555)
Expanded routine with booster			
Uncomplicated	79,300 (52,661-90,279)	179,372 (121,591-211,522)	250,819 (173,421-300,168)
Severe	2,060 (1,455-2,711)	3,744 (2,863-5,028)	4,839 (3,376-6,722)
Hospitalisations	982 (688-1,346)	1,786 (1,332-2,499)	2,323 (1,608-3,335)
Direct deaths	218 (137-324)	439 (305-655)	585 (385-883)
All deaths	418 (228-565)	909 (561-1122)	1333 (811-1634)
DALYs	22,409 (12,370-30,176)	48,905 (30,345-60,518)	72,513 (44,534-88,531)
Direct DALYs	12,012 (7,628-17,739)	24,429 (17,088-35,728)	33,367 (22,551-49,362)

**Table 5:** Cumulative total events averted per 100,000 fully vaccinated individuals (all ages) across 43 sub-Saharan African countries, cumulative by 5 year periods for each of the four deliveries: EPI (6–12 weeks), EPI with boosters, expanded routine (6–9 months) and expanded routine with booster

Event averted	At 5 year followup	At 10 year followup	At 15 year followup
EPI (6-12 weeks)			
Uncomplicated	62,110 (40,590-70,600)	61,750 (41,890-73,220)	55,010 (38,750-66,240)
Severe	1,500 (1,040-2,100)	1,080 (480-1,630)	810 (120-1,450)
Hospitalisations	720 (490-1,060)	520 (220-810)	400 (60-710)
Direct deaths	180 (110-290)	150 (60-240)	130 (40-210)
All deaths	430 (200-550)	430 (230-580)	410 (240-560)
DALYs	23,120 (10,990-29,720)	23,080 (12,640-30,790)	22,370 (12,930-29,910)
Direct DALYs	9,810 (6,110-16,010)	8,600 (3,860-13,210)	7,420 (2,680-11,990)
EPI with booster			
Uncomplicated	83,130 (54,620-94,170)	90,170 (60,770-106,350)	81,720 (57,160-97,700)
Severe	2,080 (1,460-2,850)	1690 (1,060-2,410)	1,340 (600-2,050)
Hospitalisations	1,000 (690-1,410)	810 (510-1,180)	650 (300-990)
Direct deaths	240 (150-360)	230 (140-340)	200 (100-300)
All deaths	530 (270-700)	550 (320-690)	540 (310-690)
DALYs	28,540 (14,830-37,290)	29,760 (17,290-37,130)	29,050 (17,030-37,190)
Direct DALYs	13,250 (8,220-19,680)	12,670 (7,830-18,840)	11,190 (6,300-17,200)
Expanded routine (6-9 months)			
Uncomplicated	66,310 (43,570-75,950)	65,990 (44,410-78,410)	58,130 (40,080-69,970)
Severe	1,740 (1200-2,340)	1,410 (990-1,930)	1160 (710-1,650)
Hospitalisations	830 (570-1,190)	680 (460-960)	560 (320-830)
Direct deaths	180 (100-300)	170 (100-270)	150 (80-230)
All deaths	370 (180-510)	380 (210-490)	360 (180-460)
DALYs	20,120 (9,960-27,390)	20,380 (11,250-26,430)	19,620 (9,970-25,100)
Direct DALYs	10,200 (5,690-16,550)	9,460 (5,500-14,800)	8,320 (4,790-13,080)
Expanded routine with booster			
Uncomplicated	90,300 (59,970-102,800)	99,120 (67,190-116,890)	89,750 (62,050-107,410)
Severe	2,350 (1,660-3,090)	2,070 (1,580-2,780)	1,730 (1,210-2,410)
Hospitalisations	1,120 (780-1,530)	990 (740-1,380)	830 (580-1,190)
Direct deaths	250 (160-370)	240 (170-360)	210 (140-320)
All deaths	480 (260-640)	500 (310-620)	480 (290-580)
DALYs	25,520 (14,090-34,360)	27,020 (16,770-33,440)	25,950 (15,940-31,680)
Direct DALYs	13,680 (8,690-20,200)	13,500 (9,440-19,740)	11,940 (8,070-17,660)

**Fig. 5:**
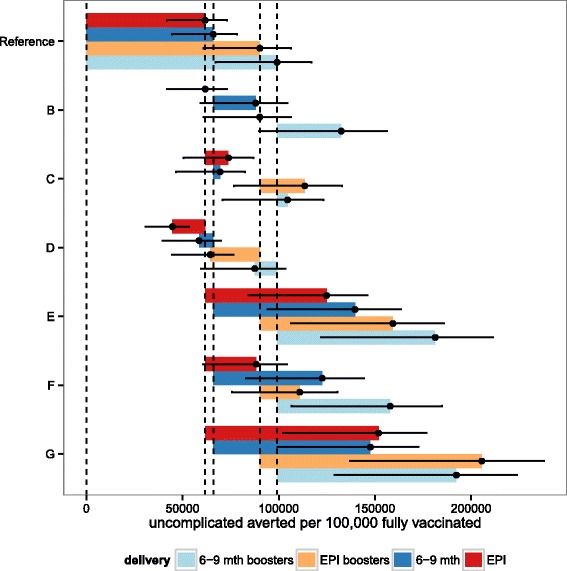
Predicted cumulative uncomplicated cases averted per 100,000 fully vaccinated over 10 years for sub-Saharan Africa for each of the four vaccine implementations: EPI, EPI with boosters, extended routine and extended routine with boosters. Predictions of the overall number of uncomplicated cases averted per 100,000 fully vaccinated over ten years, for vaccine and coverage sensitivities B-G (see Table 3), for EPI (red), EPI with boosters (orange), extended routine (dark blue), and extended routine with booster (light blue). Points correspond to the means of the predictions based on weighted averages over all simulations of the vaccine profile. Vertical lines correspond to the means of the predictions for the reference vaccine profile for each of the four vaccination schedules. Error bars represent the minima and maxima of the predictions based on replication of the simulations with 6 different model variants each with 5 random number seeds

**Fig. 6:**
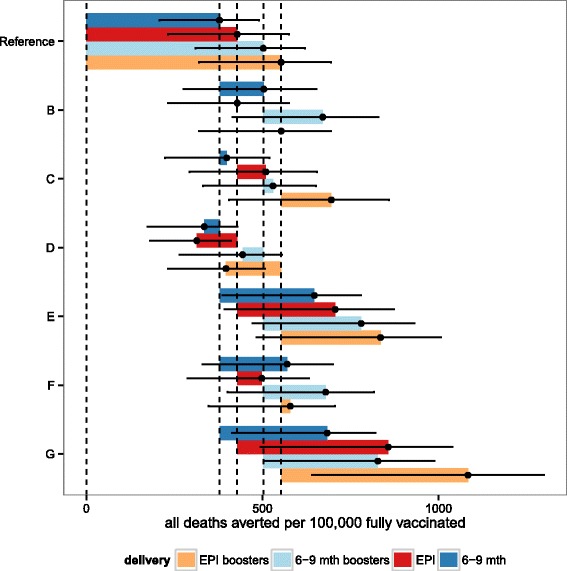
Predicted cumulative all deaths averted per 100,000 fully vaccinated over 10 years for sub-Saharan Africa for each of the four vaccine implementations: EPI, EPI with boosters, extended routine and extended routine with boosters. Predictions of the overall number of all cause deaths averted per 100,000 fully vaccinated over ten years, for vaccine and coverage sensitivities B-G (see Table 3), for EPI (red), EPI with boosters (orange), extended routine (dark blue), and extended routine with booster (light blue). Points correspond to the means of the predictions based on weighted averages over all simulations of the vaccine profile. Vertical lines correspond to the means of the predictions for the reference vaccine profile for each of the four vaccination schedules. Error bars represent the minima and maxima of the predictions based on replication of the simulations with 6 different model variants each with 5 random number seeds

**Fig. 7:**
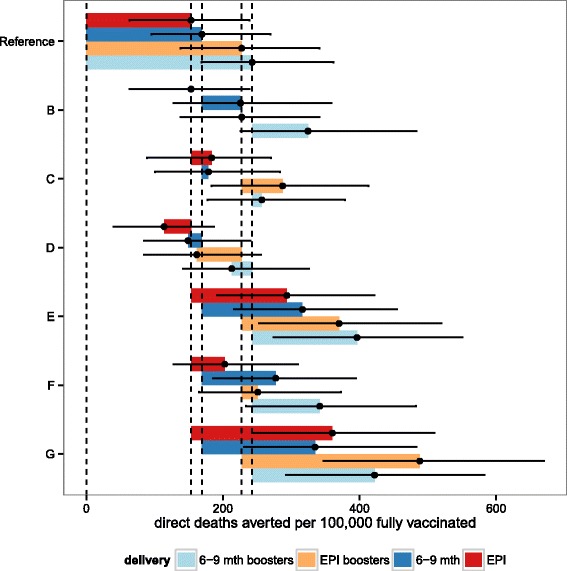
Predicted cumulative direct deaths averted per 100,000 fully vaccinated over 10 years for sub-Saharan Africa for each of the four vaccine implementations: EPI, EPI with boosters, extended routine and extended routine with boosters. Predictions of the overall number of direct malaria deaths averted per 100,000 fully vaccinated over ten years, for vaccine and coverage sensitivities B-G (see Table 3), for EPI (red), EPI with boosters (orange), extended routine (dark blue), and extended routine with booster (light blue). Points correspond to the means of the predictions based on weighted averages over all simulations of the vaccine profile. Vertical lines correspond to the means of the predictions for the reference vaccine profile for each of the four vaccination schedules. Error bars represent the minima and maxima of the predictions based on replication of the simulations with 6 different model variants each with 5 random number seeds

**Fig. 8:**
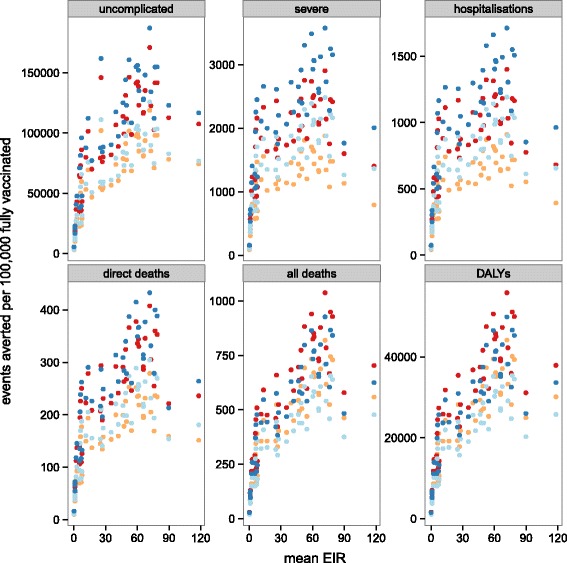
Mean predicted cumulative events averted per 100,000 fully vaccinated over 10 years by mean level of transmission (EIR) for sub-Saharan Africa for each of the four vaccine implementations: EPI, EPI with boosters, extended routine and extended routine with boosters. Predictions of the overall number of different events averted per 100,000 fully vaccinated over ten years, for vaccine reference profile (see Table 3) by mean level of transmission for each country. Immunisation strategies are EPI (red), EPI with boosters (orange), extended routine (dark blue), and extended routine with booster (light blue). Points correspond to the means of the predictions based on weighted averages over all simulations of the vaccine profile

For each outcome, the uncertainty ranges for the different deployment schedules overlap for the predictions of both numbers and proportions of events that would be averted by vaccination. In general the ranking of predictions is similar, whether the results are expressed as total numbers of events averted, events averted per 100,000 fully vaccinated children, or as percentages of the total burden averted. When the best fitting (reference) vaccine profile is assumed, EPI vaccination is predicted to avert more deaths than vaccination of 6–9 month old children over a 10 year time horizon (Figs. 6 and 8), though when indirect mortality is excluded and the results expressed as deaths averted per 100,000 fully vaccinated children, the point prediction is higher for vaccination at 6–9 months (Fig. 7). Similarly the total number of cases averted is higher with EPI (Fig. 5), but cases averted per 100,000 fully vaccinated children is somewhat higher with vaccination at 6–9 months, though in all these analyses the uncertainty intervals overlap. Adding a booster dose to the schedule increases the effects roughly in proportion to the total number of doses administered (Figs. 5, 6, 7 and 8).

Comparison of these predictions with those based on vaccine profiles with less support from the data indicates the sensitivity of these results to the main uncertainties in the profiles. Each of panels B-G in Figs. 5, 6 and 7 (and Additional file 2: Figures S9-S11) corresponds to a set of alternative assumptions described in Table 3. The effects of improvements in coverage for 6–9 month vaccination, small increases (C) or decreases in initial efficacy (D), are small, while increases in the half-life of the vaccine effect are substantial (E) especially if accompanied by an increase in initial efficacy (G). If initial efficacy is decreased, and half-life increased to give a profile similar to that estimated from Phase II data [8], the effect is a small improvement in each of the measures of public health impact, but the uncertainty bounds overlap with those for the reference scenarios. The impacts of other country-specific assumptions have been quantified in a simple sensitivity analysis on country levels of transmission exposure, access to effective treatment and reduced coverage of vaccination (Additional file 1: Table SM1 and Additional file 2: Figures S12-S13). If transmission levels decrease or increase (by 50 %, Additional file 2: Figure S12-S13 (I,J)) the impact is not as significant as the impact of increased or decreased access to effective treatment (Additional file 2: Figure S12-S13 (I,J)).

**Fig. 9:**
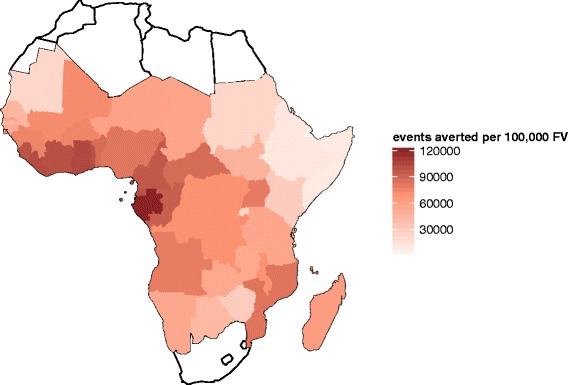
Mean predicted total uncomplicated and severe events averted per 100,000 fully vaccinated after 10 years by country for EPI (6–12 weeks) immunisation schedule. Cumulative total uncomplicated and severe events averted per 100,000 fully vaccinated by country, cumulative at 10 years post introduction immunising via EPI routine immunisation schedule of 6–12 weeks (vaccination coverage is at DTP3 levels of country immunisation)

**Fig. 10:**
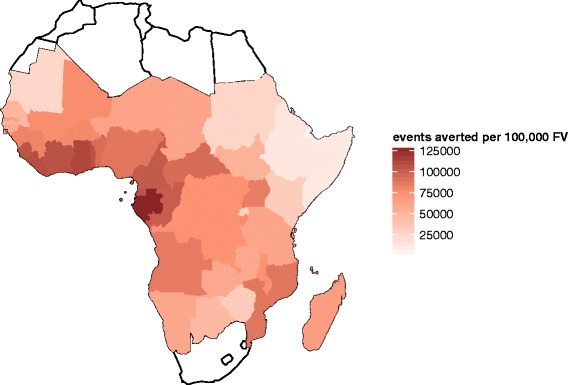
Mean predicted total uncomplicated and severe events averted per 100,000 fully vaccinated after 10 years by country for extended routine (6–9 month) immunisation schedule. Cumulative total uncomplicated and severe events averted per 100,000 fully vaccinated by country, cumulative at 10 years post introduction immunising via extended routine immunisation schedule of 6–9 months (vaccination coverage is at 75 % of DTP3 levels of country immunisation)

These overall results average out considerable variation between countries in the predicted impact (Figs. 8, 9 and 10, and Additional file 3: Tables P1-P8). The distributions of transmission intensity are the main driver of this (Fig. 8 and estimates by map in Figs. 9 and 10 and Additional file 2: Figures S16-S23). Previous analyses have found that public health impact of pre-erythrocytic vaccines will be highest at intermediate transmission intensities, where there are enough infections to make prevention worthwhile but where the parasite challenge is not so great as to drown the effect of the vaccine [9, 17]. The current analysis indicates that there is a strong general increase in impact with average level of transmission at the country level, indicating that in only a few countries (for example, Burkina Faso) are substantial proportions of the population in the range where vaccine effectiveness is compromised by an overwhelming parasitological challenge. There is a decrease in effectiveness (the percentage of events averted) with increasing transmission rate, especially for severe disease and hospitalisation (Additional file 2: Figure S14). The effectiveness of vaccination increases with access to effective treatment at the country level (Additional file 2: Figure S15), but absolute numbers of events averted is predicted to be lower with increased access to effective treatment.

## Discussion and conclusions

Simulation models of the public health impact of pre-erythrocytic vaccines against malaria are not new, but there is new urgency in making specific predictions for RTS,S/AS01 linked to the malaria situation in endemic countries using available Phase III data to parameterise models. The reason is that a recommendation on the use of RTS,S is expected as early as the end of 2015. Previously the public health impact of introducing the RTS,S vaccine into routine vaccination schedules in Africa has been difficult to predict because available clinical trial data were inadequate for accurately estimating the kinetics of vaccine protection, and this uncertainty in the vaccine profile meant that geographically specific predictions of likely impact [17] were mainly of value to indicate general principles and data gaps. Site- and time- specific data from 18 months of follow-up of the Phase III trials [1] have now enabled us to estimate the vaccine profile accurately enough for quantitative predictions of impact at national level to have sufficient plausibility for guiding policy decision as well as for informing subsequent implementation decisions by ministries of health.

Using available clinical trial data, the estimate of the initial efficacy against infection of RTS,S/AS01 is around 63 % (95 % CI 39.5–80.3 %) for infants and 79.2 % (95 % CI 67.3–84.8 %) for children, and is slightly higher than the efficacy in challenge trials which directly estimate the same quantity. In challenge trials with RTS,S in adults, 42 % [28] and 47 % [29] protection against an infection challenge was observed with adjuvant AS02, and 50 % observed when using adjuvant AS01B [30]. Consistent with our results is the almost equivalent estimate obtained with natural challenge of 65.9 % (95 % CI 42.6–79.8 %) protection against first infection in a Phase I/IIb trial immunising infants with RTS,S/AS01 [31]. The model estimates for RTS,S/AS01 initial efficacy against infection in this work are substantially higher than those previously estimated by modelling from the initial Phase II RTS,S/AS02 of 52 % [16], and, as expected, higher than the directly measured efficacy against clinical episodes at 18 months follow-up [1]. However, there is considerable uncertainty around them, especially for the 6–12 week cohort.

The underlying vaccine profile of efficacy against infection and decay, which reflects the induced pre-erythrocytic immunity, is most likely the same across the trial sites, even though the measured clinical efficacy which also depends on secondary effects on blood stage immunity, appears to be lower in sites with higher exposure [1]. This effect can be accounted for by between-site variation in transmission level, the extent of transmission heterogeneity, and in levels of access to care, all of which modify the relationship between the underlying efficacy in preventing infection and efficacy against clinical disease, justifying our use of site-independent estimates of the underlying initial efficacy and decay.

RTS,S initial protection is high and decays relatively rapidly and although clinical efficacy over time might seem low, RTS,S implemented in addition to current malaria control measures across endemic countries in Africa will have substantial impact in averting malaria cases. RTS,S would avert 100–580 malaria deaths and 45,000 to 80,000 clinical events for every 100,000 fully vaccinated child in the first 10 years of the program. This would potentially increase if boosting doses are added. The uncertainty in the vaccine profile is compounded in these predictions of public health impact by the uncertainty in the distributions of transmission levels in the different countries. This does not even take into consideration the uncertainties in demographic projections, in future trends of malaria and control, and in the assumptions about vaccination coverage; with coverage levels and population growth in higher transmission areas predicted to have a much larger impact than uncertainty in future trends of transmission. In addition, the differences in predicted impact between the vaccination schedules are small in relation to the uncertainty ranges. In particular, the predictions of public health impact of EPI vaccination and vaccination at 6–9 months are very similar, with the former averting overall slightly more episodes of illness, and the latter overall more deaths depending on coverage (a consequence of the age dependence in the case fatality rate and association with indirect mortality due to co-morbidities at younger ages [26]).

Previous simulations of the effects of paediatric malaria vaccination programs demonstrated minimal herd immunity effects [9], meaning that this intervention strategy will not have any substantial effect on overall levels of malaria transmission. This is a consequence of the targeting of a narrow age range (those at highest risk of life-threatening disease) to vaccinate, not of the vaccine profile per se. Indeed, the high initial efficacy of RTS,S/AS01 is similar to the profile aimed at for vaccines aiming to interrupt transmission [32], and mass administration of a vaccine with such a high efficacy would have substantial transmission effects [9]. However, the current strategy for licensure of RTS,S does not envisage mass vaccination, and this is outside the scope of this paper, but previous efforts have indicated the potential benefits in low transmission settings [9]. Post-registration use of the vaccine will be important, as will further modelling investigations.

The availability of very extensive data on prevalence from MAP [21] means that there is a better basis for estimating the vaccine-avertable burden of disease for malaria than for other major childhood infections. The high burden of *Plasmodium falciparum* disease means that we predict the public health impact of RTS,S to be comparable to that of other new childhood vaccines, such as those against Haemophilus influenza type b and pneumococcus, despite the leakiness and relatively low efficacy of the vaccine. Such a large public health impact is based on much higher rates of severe disease and mortality than have been observed in the trials (where severe disease rates were low and malaria mortality almost absent, presumably because very high standards of care were achieved [1]). These higher levels of disease are those measured in the non-trial datasets to which the OpenMalaria models were originally fitted [11, 26]. For comparisons with other vaccines it is also relevant to consider that some deaths arising from co-infections could be averted by vaccination against either of the pathogens concerned. This particularly applies to our simulated numbers of indirect malaria deaths, which are intended to capture the effects of interactions between *Plasmodium falciparum* and co-infections, especially respiratory bacteria.

A very important source of uncertainty in our predictions is in the kinetics of the vaccine effect on infection rates. The analysis suggests that the efficacy in preventing infections decays exponentially with a half-life of decay of around 1 year (Table 2), which is much faster than was previously thought but is in line with published data of IgM serum concentrations [8]. The public health impact will depend on not just the half-life, but also the functional form of efficacy decay. Once data from longer follow-up periods of the trial are available it should become possible to estimate whether decay curves belonging to families other than the exponential are more appropriate. In line with previous analyses [11] we infer that the efficacy measured against clinical malaria in the trial is declining over time even more rapidly than the underlying effect in preventing new infections, so the superficial interpretation that the decline in efficacy means that vaccination has only a transient effect should be resisted. Conversely, the temptation should be resisted to present efficacy as values cumulated up to specific time points, which makes waning of efficacy less evident. It is essential to compare incidence between the arms of the trial over each time interval, allowing recurrent events in the same children. However, the prediction that the time-period specific efficacy in some trial sites may fall below zero by the end of the trial, based on extrapolating the existing decay, highlights the need to manage expectations so that such a result is not misinterpreted. This is an unavoidable property of a leaky vaccine combatting recurrent challenges from a pathogen that stimulates partial immunity. Some clinical events in vaccinated children will be delayed, rather than averted, a phenomenon that must be taken into account in predicting public health impact of all partially protective malaria interventions, but which should not be interpreted as an adverse effect of vaccination.

Data are still being accrued that will be crucial for estimating the shape of the efficacy decay, and the estimation will be repeated when the results from the full follow-up of 32 months are available. This analysis will also enable us to assess whether a different efficacy for the boosting dose is expected compared to the third dose given 18 months prior to boost. This will considerably reduce the uncertainty in predictions of the effect of boosting.

All models assume no rapid evolution of the parasite sensitivity to the RTS,S antigen, and fears over resistance are actually small, but this should not impact the evaluation of a new intervention with the potential to prevent malaria morbidity and mortality.

Since the computational requirements of our analysis were enormous, with each of the simulations from OpenMalaria requiring significant computing time, repeating the analysis is not a trivial exercise. However, a distinct benefit of our data-basing and weighting approach is that estimates for different countries, trial sites or geographical areas with different transmission and health system parameters can be made without running new micro-simulations. Only the fitting and weighting steps will need to be repeated when new trial data are available, and these have comparatively low computational requirements. Bayesian MCMC estimation of weighting factors also provides a way to fit the very complex OpenMalaria models simultaneously to multiple outcomes from the trials (prevalence and clinical incidence) without the computationally expensive need to re-run the simulations iteratively. Other advantages offered by the model averaging approach over estimates based on single parameterisations include the propagation of the uncertainty in the vaccine profile through to the public health impact predictions, allowing the influence of these factors to be compared with the sensitivity to assumptions about transmission and health systems. Weighted averaging of simulations also provides a straightforward approach for analysis by repeating the calculation of public health impact, using different weight vectors. The use of a model ensemble capturing different assumptions around development of immunity and degree of transmission heterogeneity also provides lower-bound estimates of the impact of structural uncertainty [11], and replicating simulations with random number seeds tells us how much stochasticity influences our results.

An additional key message from this analysis is that the decay in efficacy is the parameter contributing the most uncertainty to the prediction of public health impact of RTS,S and for second generation malaria pre-erythrocytic vaccines. Other promising pre-erythrocytic vaccines have already demonstrated near 100 % efficacy in challenge trials [33] before rechallenge. The developers of these vaccines also need to consider that, while a high initial efficacy is clearly highly desirable, the temporal pattern of decay in efficacy is of equal, if not more, importance as a determinant of the likely public health impact of vaccination programs.

## Additional files

Additional file 1
**Supplementary methods.** Detailed supplementary methods including tables and figures associated with model fitting and predictions.

Additional file 2
**Supplementary results 1.** Additional tables associated with fitting of vaccine properties and country-specific predictions of vaccine impact.

Additional file 3
**Supplementary results 2.** Additional tables associated with country-specific predictions of vaccine impact.

## Abbreviations

ATP: according to protocol
CSP: circumsporozoite protein
DALYs: disability adjusted life years DIC: deviance information criterion
DTP: diphtheria-tetanus-pertussis
EIR: entomological inoculation rates
EPI: Expanded Program on Immunisation
MAP: Malaria Atlas Project
MCMC: Markov chain Monte Carlo
