# Supplementary material for: The public health impact of malaria vaccine RTS,S in malaria endemic Africa: country-specific predictions using 18 month follow-up Phase III data and simulation models
Source: BMC Med. 2015 Jul 29;13:170. doi: 10.1186/s12916-015-0408-2 (PMC4518512; doi:10.1186/s12916-015-0408-2)
Supplement: Additional file 1 — Supplementary methods. Detailed supplementary methods including tables and figures associated with model fitting and predictions. [file 12916_2015_408_MOESM1_ESM.pdf]

The public health impact of malaria vaccine RTS,S in malaria endemic Africa: country-specific predictions using 18 month follow-up Phase III data and simulation models

Melissa A Penny*[1,2], Katya Galactionova[1,2], Michael Tarantino[1,2], Marcel Tanner[1,2], Thomas A Smith[1,2]

[1] Swiss Tropical and Public Health Institute, Basel, Switzerland
[2] University of Basel, Basel, Switzerland

* Corresponding Author:
Melissa A Penny
Department of Epidemiology and Public Health
Swiss Tropical and Public Health Institute
Socinstrasse 57, 4051 Basel, Switzerland
email: melissa.penny@unibas.ch

Additional File 1: Supplementary Methods

Computing weighted predictions and corresponding weights

To obtain a prediction via weighted averages over all simulations for a given vaccine delivery schedule or vaccinated cohort for a given country or trial site and vaccine profile, for a certain outcome O, the following formula is used,

$$\mu_{\mathrm{O}}(c,i) = \sum_{j,k,l,m,n,p} \omega_{\mathrm{eir}}(j)\omega_{\mathrm{eff}}(k)\omega_{\mathrm{half-life}}(l)\omega_{\mathrm{decSh}}(m)\omega_{\mathrm{accU}}(n)\omega_{\mathrm{accS}}(p)O(c,i,j,k,l,m,n,p) \qquad (1)$$

where $\mu_{\mathrm{O}}(c,i)$ represents a weighted average of outcome O for a particular coverage level c and model i. Output $\mathrm{O}(c,i,j,k,l,m,n,p)$ represents the predicted outcome from a scenario (calculated separately for a number of seeds (used to initialise the random number stream)) corresponding to coverage level c, model i, EIR j, efficacy k, half-life l, decay shape m, access to uncomplicated care n and access for severe care p. The weights are represented via vectors ω_{name} where name refers to variables model (mod), EIR (eir), efficacy (eff), half-life (halflife), decay shape (decSh), access for uncomplicated (accU), access for severe (accS). The summation subscripts j,k,l,m,n,p correspond to level of each variable, for example j = 1, ..., 8 for EIR levels. The number of levels in each variable depends on the final simulation structure (see Table 1 (main text))

For all predictions we present mean weighted averages and report the range via minimum and maximum limits over the weighted averages for all models and seeds, without model weighting. We do this to reflect the full structural and stochastic uncertainty in the mode.

Pre-erthrocytic vaccine efficacy and decay

The action of a pre-erythrocytic vaccine like RTS,S is input to the models as vaccine efficacy in preventing a new infection. This corresponds to the proportion of blood stage infections averted, and hence is similar to the efficacy measured in a sporozoite challenge trial. This is different from the efficacy in averting clinical episodes as reported in the Phase III clinical trials, which differs from the simulated efficacy both in average value and in the way in which it evolves over time, with factors including transmission heterogeneity and age-shifting of susceptibility leading to greater decays over time in field-measurable quantities than in the underlying efficacy against infection assumed in the models [1].

OpenMalaria allows different rates of decay [2] in underlying efficacy over time and different shapes of the decay. For fitting, decay was assumed to follow a Weibull curve described by the initial value of the efficacy, the half-life, and a shape parameter, k. Simulations were carried out with shape parameter k with values of $0.5, 1$ or 2, where $k = 1$ corresponds to exponential decay. The Weibull decay function takes the form

$$\epsilon(t) = \epsilon_0 \exp\left[\frac{-(\log(2))^{\frac{1}{k}} t}{L^k}\right]. \tag{2}$$

Here $\epsilon(t)$ is the efficacy against infection at time t, ϵ_0 the initial efficacy against infection of the vaccine at last given dose of vaccine, and L (years) is the half-life of efficacy. Decay shapes for different values of shape parameter k and half-life L are illustrated in Figure SM2. When $k = 1$, an exponential decay of efficacy against infection is obtained. For k less than 1, the initial decay is faster than exponential and then slower than exponential after the time equivalent to half-life is reached, this is similar to a bi-phasic like decay [3, 4], with a sharp decline (quick decay) in efficacy followed by longer decay. For k greater than 1 we observe slow decay of efficacy against infection until the time equivalent to half-life L, and then a much faster decay.

Methodology associated with parameterising vaccine properties

Calculating disease rates and clinical efficacy

The disease rate, the number of events per person in a given period is given by

$$D_x = \frac{E_x(t)}{H_x(t)}. \tag{3}$$

Here, D_x represents the disease rate for group x (control c or vaccinated v) for 6-month period t, E_x the number of events recorded in the corresponding six month period and H_x the number of individuals at risk.

The simulation model results were fitted to the disease rates from the trial, not the clinical efficacy. The clinical efficacy predicted by the models can be calculated as

$$\eta(t) = 1 - \frac{D_v(t)}{D_c(t)}, \tag{4}$$

which is 1 minus the vaccinated disease rate divided by the control disease rate.

Malaria Exposure in the trial sites

The estimates of vaccine properties were made conditional on estimates of the exposure to malaria in each of the 11 trial sites. Malaria exposure as a model input is the EIR and we use two different approaches to estimating site-specific EIR distributions:

i. Estimates based on the MAP 2010 prevalence surfaces [5] for the district in which the trial site is located. For this method MAP prevalence and the OpenMalaria model relationship between EIR and prevalence along with site-specific access to effective care was used to derive distributions of exposure [6]. The access to effective care assumed for each trial site was a scaled estimate from DHS admin-1 [7].

ii. Estimates adjusted to account for differences between MAP-based prevalence and observed trial site prevalence calculated from Phase III trial data [8]. We estimated a relationship between OpenMalaria model estimates of infant prevalence versus predicted 2-10 years prevalence (the age group represented by MAP data). And using relationship between OpenMalaria model input EIR vs

predicted 2-10 years prevalence. These two relationships were then combined to estimate a functional dependence between infant prevalence and EIR. Using the site-specific estimates of prevalence (recorded as parasite positivity in the trial control group), transformations were calculated on a per-model basis and applied to each of the EIR distributions calculated in (i). The net effect for all sites and all models was a reduction in effective EIR.

Estimate (i) is likely to over-estimate of exposure in the trial sites given that it does not allow for the fact these intensively studied sites generally have higher intervention coverages (especially of bednets) than neighbouring areas. All analysis in this paper was completed with both transmission assumptions (i) and (ii), but for the majority of the time we present results for assumption (ii).

Determining best fitted model

To compare the different fitted models and determine the most appropriate fitted model for final vaccine parameters, the Deviance Information Criterion (DIC) was calculated. The DIC is comprised of two terms, the posterior mean of the deviance, $\bar{D}$, and the effective number of parameters, p_D. The deviance, D is given by,

$$D = -2\log\left(p(y|\theta)\right), \tag{5}$$

where, $p(y|\theta)$ is the likelihood, and the lower this value the easier the model. The effective number of parameters penalises the DIC when more parameters are being fit and is the difference between the posterior mean of the deviance and the deviance of the posterior means. The DIC is given by

$$DIC = \hat{D} + P_D. \tag{6}$$

In general, when comparing two models fitted to the same data set, differences in DIC greater than 10 would favour the model with the lower DIC. For differences between DIC that are less than 10, it would be difficult to completely rule out the model with the higher DIC.

Methodology associated with country-specific predictions of public health impact of RTS,S

Country specific vaccine coverage and implementation

For purposes of comparison across countries we assume vaccine introduction is at the beginning of 2017 for all countries and that country specific immunisation coverage levels for RTS,S delivered via routine EPI are based on the third dose of DTP reported by UNICEF/WHO for EPI in 2012 [9]. No scale up is assumed for simplicity and to avoid erroneous assumptions 2012 levels are assumed at 2017 up to 2030.

EPI coverage levels are scaled by 75% for expanded routine (6-9 months) delivery. Table SM2 summarises the coverage levels for vaccination via EPI and via expanded routine (6-9 months) for each of the 43 countries, respectively. In addition, boosting schedules for EPI and expanded routine assume 80% coverage of the third dose for that schedule. We note that these coverage values are controversial [10] and that the WHO-UNICEF values for DTP3 may slightly be optimistic. An overestimation of the coverage achieved will lead to overestimation of the public health impact of the vaccine program.

Vaccine coverage is simulated by treating a vaccinated population with coverage c as a weighted average of c times a population with coverage 100% and $(1-c)$ times a population with coverage 0%. This linearization is justified by the result from previous analyses that herd immunity effects are negligible in EPI and EPI with booster simulations using these models [1,11], so the vaccine effects on populations can be treated linear functions of the contributions of individuals with different ages and vaccination histories to the population.

To make weighted predictions for the boosting schedule for with EPI or expanded routine, the simulation databases for both the boosting and 3 dose schedule are used where $80\%c$ of the boosting schedule

is used, and 20%c of the appropriate 3 dose schedule and $(1-c)$ times a population with 0% coverage, where c is the country specific coverage level for that routine without booster.

Country specific transmission, population levels and health systems parameters and weights

Population numbers of surviving infants after first year of life and the total population over all ages are projected from UN statistics [12].

The level of malaria transmission, via distributions of EIR, for a particular country was estimated based on the MAP 2010 prevalence surfaces [5] for geographic area in question. For this method MAP prevalence and the OpenMalaria model relationship between EIR and prevalence, along with country specific access to effective care, was used to derive distributions of exposure [6]. The percentage of the population in each EIR simulated level for this approach, as well as summary statistics arithmetic and geometric mean (and interquartile ranges), are given in [6] and Table SM2.

For purposes of our predictions we use a simplification of the full functionality of the OpenMalaria case-management model for uncomplicated malaria [13], namely we capture all variations in case management effectiveness (including imperfect efficacy of treatment) in the parameter denoting access. Country specific access to uncomplicated malaria case management is the probability of accessing effective treatment of case management, during any 5-day period. This is dependent on country specific estimates of access to 100% effective care given a malaria fever [7] and a conversion to 5-day probability detailed in [14]. These estimates are detailed in Table SM2.

Access to health care for severe cases was as assumed for previously published work [15]. This assumes a 5 day probability of 48% from [16]. Respective weights for the simulated levels of severe access were calculated to achieve 48%.

DALY calculations

Health outcomes are expressed in terms of disability adjusted life years(DALYs) on the basis of the duration of disability and respective disability weights. Years of life lived with disability (YLDs) are calculated using standard methods [17] on the basis of the duration of disability, and respective disability weights. These weights for different malaria-attributable disease conditions were obtained from the Global Burden of Disease (GBD) study. [13, 18]

Years of life lost (YLLs) and DALYs are calculated assuming age-specific life expectancies, based on the life-table from Butajira, Ethiopia, with an average life expectancy of 46.6 years at birth [19]. This life-table was chosen because it represents a sub-Saharan African setting that is characterized by low malaria transmission, so that the survival may be close to what we would expect without malaria. For example, it is very similar to that for Hai district, a high altitude site in Tanzania [20]. In the light of recent recommendations [21] DALYs are presented without age-weighting and undiscounted, being the current recommendation [21].

PHI calculation details

The simulations of the dynamics of malaria were carried out using approximately stable and stationary simulated human populations, with age distributions based on those in malaria endemic areas. To maintain these age distributions, age-specific out-migrations (as well as deaths) were also simulated [22]. Given that our predictions are a weighted average from a large number of simulations and that vaccination coverage is changing in time we must allow for events recorded for different age groups to be associated with the coverage levels of the time in which they would have been vaccinated. To project effects of national level scale up of vaccination for specific years and real growing populations, the results were post-processed as follows:

The number of events averted, $\tilde{E}_i(t,\tau)$, in year t, among individuals of age τ years in a simulated stable and stationary population of 100,000 people in country i is obtained by weighting each simulation

j, summing over all the simulations, and multiplying by the coverage applicable in the year that these individuals were originally vaccinated, i.e.: ,

$$\tilde{E}_i(t,\tau) = c_i(t-\tau)\sum_j \hat{\omega}_{i,j} E_j(\tau), \tag{7}$$

where $E_j(\tau)$ is the events averted in vaccination simulation j at coverage 100% in individuals of age τ years obtained by matching with a corresponding non-vaccination simulation, both with population 100,000; $\hat{\omega}_{i,j}$ is the weight corresponding to simulation j (a multiplication of many weights); $c_i(t-\tau)$ is the vaccine coverage in country i in year $(t-\tau)$. Our recorded age-groups were not evenly spaced and for ages greater than 6 years, events were recorded in age categories greater than 1 year. In this case the $c_i(t-\tau)$ is a weighted average over the coverages when that age category would have been vaccinated, namely

$$c_i(t-\tau) = \sum_{k=l}^{\tau} c_i(t-\tau)/(\tau-l+1), \tag{8}$$

where l is the lower bound of the age group τ , and when $\tau = t$ all coverage in that age category are used in the weighting and the denominator is the size of the age category. Summing over all age groups, τ , and allowing for population growth, the total number of events averted during each calendar year, t, in country i was estimated as:

$$E_i(t) = P_i(t)\sum_\tau \tilde{E}_i(t,\tau)/S(t), \tag{9}$$

where $P_i(t)$ is the total population of country i and $S(t)$ the simulated population size of 100,000. The cumulative number of events averted, $E_{i,cum}(t)$ at time t is thus

$$E_{i,cum}(t) = \sum_{v=1}^{t} P_i(v)\sum_\tau \tilde{E}_i(v,\tau)/S(t). \tag{10}$$

The corresponding number of vaccine doses, and the number of fully vaccinated each year, was obtained from the expected coverage in real programs, rather than by counting the vaccinations internal to the simulation. The number of vaccine doses, for a three dose schedule vaccination campaign, in a given year t is

$$V_i(t) = 3c_i(t)B_i(t), \tag{11}$$

., where $B_i(t)$ is the expected size of the cohort being vaccinated in country i at year t.

To calculate the total number of events averted per 100,000 fully vaccinated in year t, namely, $F_{i,cum}(t)$, the following formulation is required

$$F_{i,cum}(t) = P_i(t)\sum_\tau \tilde{E}_i(t,\tau)/(c_i(t)B_i(t)), \tag{12}$$

where $B_i(t))$ is the expected size of the cohort being vaccinated in country i at year t. The cumulative number of events averted per 100,000 fully vaccinated, $F_{i,cum}(t)$ at time t is thus

$$F_{i,cum}(t) = \sum_{v=1}^{t}\left(P_i(v)\sum_\tau \tilde{E}_i(v,\tau)\right) / \left(\sum_{v=1}^{t} c_i(v)B_i(v)\right). \tag{13}$$

Finally, cumulative effectiveness, $\epsilon_{i,cum}(t)$, in year t for an outcome is given by

$$\epsilon_{i,cum}(t) = \sum_{v=1}^{t}\left(\sum_\tau \tilde{E}_i(v,\tau)\right) / \left(\sum_{v=1}^{t} E_i^0(v)\right), \tag{14}$$

where $E_i^0(t)$ is the number of events observed in the no-vaccination scenario for country i.

References

1. Smith T, Ross A, Maire N, Chitnis N, Studer A, et al. (2012) Ensemble modeling of the likely public health impact of a pre-erythrocytic malaria vaccine. PLoSMed 9: e1001157–.

2. OpenMalaria (2015). OpenMalaria decay functions. URL https//github.com/SwissTPH/openmalaria/wiki/ModelDecayFunctions.

3. White MT, Bejon P, Olotu A, Griffin JT, Riley EM, et al. (2013) The relationship between RTS,S vaccine-induced antibodies, CD4(+) T cell responses and protection against *Plasmodium falciparum* infection. PLoS ONE 8: e61395–.

4. White MT, Bejon P, Olotu A, Griffin JT, Bojang K, et al. (2014) A combined analysis of immunogenicity, antibody kinetics and vaccine efficacy from Phase 2 trials of the RTS,S malaria vaccine. BMC medicine 12: 117.

5. Gething PW, Patil AP, Smith DL, Guerra CA, Elyazar IR, et al. (2011) A new world malaria map: *Plasmodium falciparum* endemicity in 2010. Malaria Journal 10: 378–.

6. Penny MA, Maire N, Bever C, Pemberton-Ross P, Briet OJ, et al. (2015) Distributions of malaria exposure in endemic countries in Africa considering country levels of effective treatment. under review .

7. Galactionova K, Tediosi F, Savigny Dd, Smith TA, Tanner M (2015) Effective coverage and systems effectiveness for malaria case management in Sub-Saharan African countries. PLoS ONE In press.

8. The RTS,S Clinical Trials Partnership (2014) (2014) Efficacy and safety of the RTS,S/AS01 malaria vaccine during 18 months after vaccination: A Phase 3 randomized, controlled trial in children and young infants at 11 African sites. PLoS Med 11: e1001685 doi:10.1371.

9. WHO-UNICEF (2014). accessed 1/6/2014. URL http//apps.who.int/immunization monitoring/globalsummary/timeseries/tswucoveragedtp3.html.

10. Lim SS, Stein DB, Charrow A, Murray CJ (2008) Tracking progress towards universal childhood immunisation and the impact of global initiatives: a systematic analysis of three-dose diphtheria, tetanus, and pertussis immunisation coverage. Lancet 372: 2031–2046.

11. Penny MA, Maire N, Studer A, Schapira A, Smith TA (2008) What should vaccine developers ask? Simulation of the effectiveness of malaria vaccines. PLoS ONE 3: e3193–.

12. United Nations DoE, Social Affairs PD (2014). URL http : //esa.un.org/wpp/Excel −Data/population.htm.

13. Tediosi F, Maire N, Smith T, Hutton G, Utzinger J, et al. (2006) An approach to model the costs and effects of case management of *Plasmodium falciparum* malaria in sub-saharan Africa. American Journal of Tropical Medicine and Hygiene 75: 90–103.

14. Crowell V, Briet OJ, Hardy D, Chitnis N, Maire N, et al. (2013) Modelling the cost-effectiveness of mass screening and treatment for reducing *Plasmodium falciparum* malaria burden. Malaria Journal 12: 4–.

15. Ross A, Maire N, Molineaux L, Smith T (2006) An epidemiologic model of severe morbidity and mortality caused by plasmodium falciparum. American Journal of Tropical Medicine and Hygiene 75: 63–73.

16. Goodman CA, Coleman PG, Mills A (2000) Economic analysis of malaria control in sub-Saharan Africa. Geneva: Global Forum for Health Research.

17. Murray CJL, Lopez AD (1996) Estimating Causes of Death: New Methods and Global and Regional Applications for 1990, Geneva: World Health Organisation. pp. –.

18. Murray CJL, Lopez AD (1996) The global burden of disease: a comprehensive assessment of mortality and disability from diseases, injuries, and risk factors in 1990 and projected to 2020. Harvard: Harvard University Press.

19. Network I (2002) Population, Health and Survival at INDEPTH Sites. Ottawa: IDRC.

20. Network I (2004) Model Life Tables for sub-Saharan Africa. Aldershot, England: Ashgate.

21. Murray CJ, Ezzati M, Flaxman AD, Lim S, Lozano R, et al. (2012) Global burden of disease 2010: design, definitions, and metrics. Lancet 380: 2063–2066.

22. Maire N, Smith T, Ross A, Owusu-Agyei S, Dietz K, et al. (2006) A model for natural immunity to asexual blood stages of plasmodium falciparum malaria in endemic areas. American Journal of Tropical Medicine and Hygiene 75: 19–31.

Tables

Additional file 1: Table SM1. Additional sensitivity analysis and reference levels of inputs

name	initial efficacy[a]	half-life[a]	Vaccine coverage	effective treatment coverage	transmission assumptions
**Reference scenario**					
Ref	EPI: 62.5%, 6-9mth: 79.2%	EPI : 1.12 year, 6-9mth: 1.12 year	EPI: DTP3 , 6-9mth: 75% of DTP3	country-specific [7]	country-specific [6]
**Country specific implementation**					
H	reference	reference	decrease (25%)	reference	reference
I	reference	reference	reference	reference	increase (50%)
J	reference	reference	reference	reference	decrease (50%)
K	reference	reference	reference	increase (25%)	reference
L	reference	reference	reference	decrease (25%)	reference
M	reference	reference	decrease (25%)	decrease (25%)	reference

[a]vaccine efficacy against infection and vaccine half-life of decay against infection.

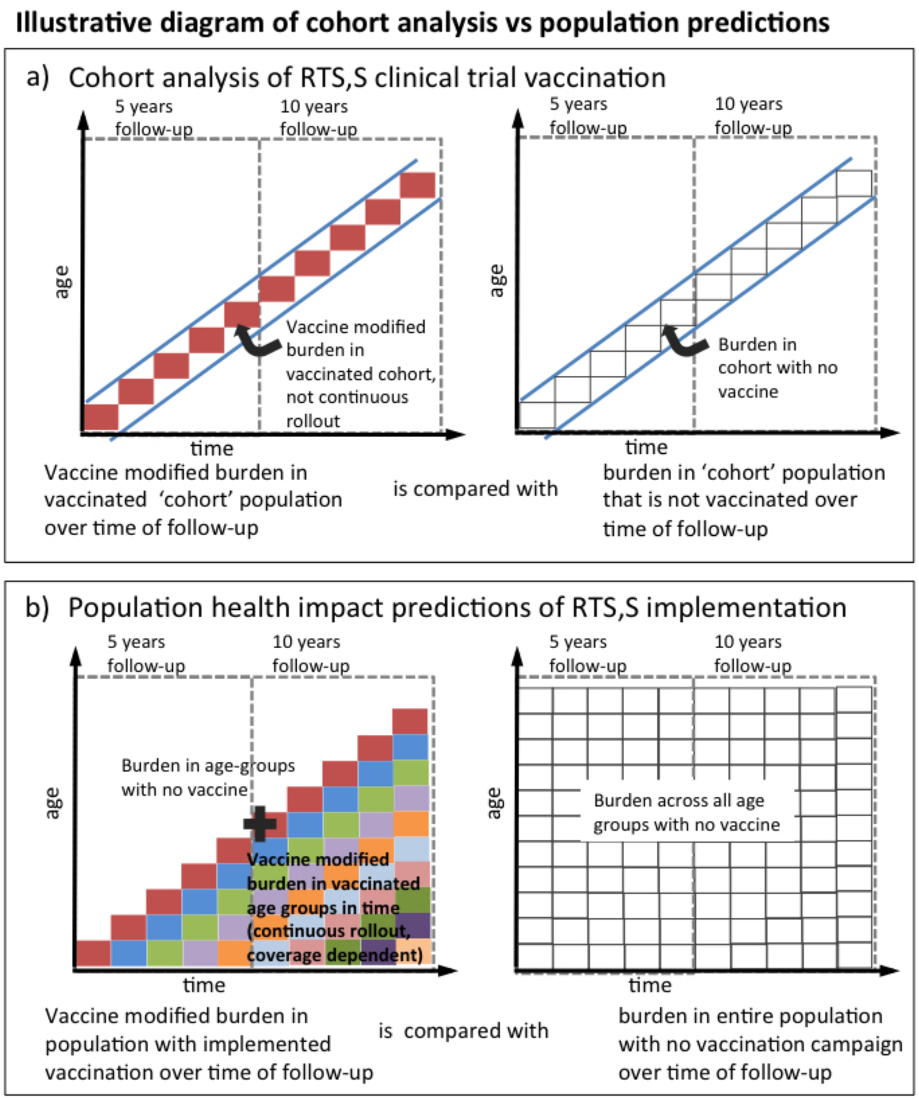

Additional file 1: Figure SM1. Lexis diagram illustrating the approaches to calculating events averted in clinical trial vs population vaccine program. a) events averted are calculating by tracking a vaccinated age group in time and comparing the burden (clinical cases, severe and death) experienced by this cohort (indicated by red boxes in left panel) compared to the same cohort with no vaccination (uncoloured boxes in the right panel). b) In a vaccination campaign with continuous coverage, the total events averted in the population is the total burden over all ages in time (uncoloured boxes in the right panel) minus the sum of the vaccine modified burden in the vaccinated population plus the burden in the non-vaccinated ages over time (coloured and non-coloured boxes in the left panel). Colours indicate vaccine modified burden.

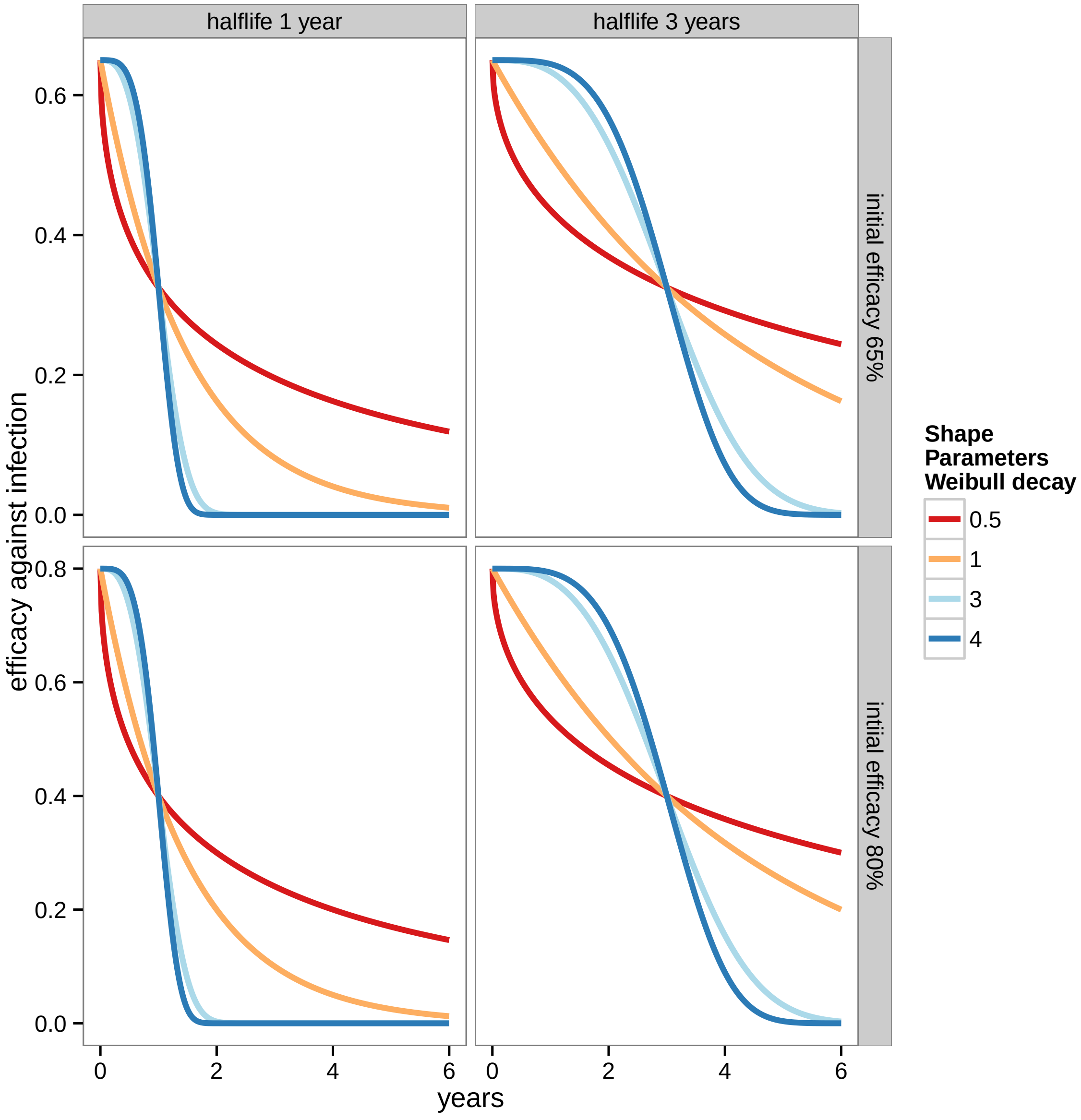

Additional file 1: Figure SM2. Weibull decay function. Decay of efficacy in time for different Weibull decay functional forms for different initial efficacy and half-life. Red indicates bi-phasic decay (shape parameter less than 1), orange exponential decay, blue more sigmoidal like decay (shape parameter greater than 1)

country	Effective coverage	DTP3 coverage	weighted mean EIR	weighted median EIR	EIR 25% quartile	EIR 75% quartile.
Angola	48.67%	91%	49.8207	6.24512	1.36697	50.43574
Benin	30.26%	85%	72.7978	13.34845	2.9218	130.34698
Botswana	71.25%	96%	5.3295	0	0	1.65285
BurkinaFaso	34.59%	90%	118.1262	73.73665	9.13031	407.32036
Burundi	42.60%	96%	13.5161	1.13055	0.24746	6.24512
Cameroon	25.92%	85%	67.4316	11.03972	1.99851	107.80241
Central Africa nRepublic	13.43%	47%	61.4501	7.55115	1.13055	89.1571
Chad	17.71%	45%	27.7761	1.65285	0.24746	11.03972
Comoros	37.61%	86%	46.5291	5.16497	0.77329	34.49795
Congo	42.88%	85%	49.0202	7.55115	1.65285	50.43574
Democratic Republic of Congo	26.87%	72%	47.4158	4.27165	0.63954	34.49795
Cote d'Ivoire	25.30%	94%	78.8227	19.51532	2.9218	157.60628
Djibouti	46.61%	81%	0.1781	0	0	0
Equatorial Guinea	19.37%	33%	76.8485	16.13999	2.41645	157.60628
Eritrea	24.71%	99%	1.058	0.07919	0.02534	0.24746
Ethiopia	15.83%	61%	0.9649	0.03705	0.00555	0.13999
Gabon	40.39%	82%	71.7469	16.13999	3.53283	130.34698
The Gambia	39.32%	98%	7.3258	1.36697	0.36179	5.16497
Ghana	39.87%	92%	52.2864	7.55115	1.65285	50.43574
Guinea	24.95%	59%	39.66	3.53283	0.63954	23.59654
Guinea Bissau	27.45%	80%	6.2679	0.77329	0.24746	2.9218
Kenya	35.69%	83%	7.6956	0.20466	0.03705	1.13055
Liberia	45.18%	77%	60.1899	16.13999	4.27165	73.73665
Madagascar	20.24%	86%	42.0316	1.99851	0.20466	23.59654
Malawi	39.05%	96%	54.5347	9.13031	1.65285	60.9833
Mali	27.51%	74%	75.9512	16.13999	2.41645	157.60628
Mauritania	22.43%	80%	5.3699	0.0655	0	0.43745
Mozambique	37.93%	76%	65.8036	11.03972	1.65285	107.80241
Namibia	37.96%	84%	11.3246	0.29921	0	2.41645
Niger	30.85%	74%	35.3448	3.53283	0.77329	19.51532
Nigeria	32.23%	41%	65.6763	11.03972	2.41645	107.80241
Rwanda	40.95%	98%	2.2015	0.24746	0.02534	0.93501
Sao Tome Principe	68.02%	96%	25.7633	6.24512	1.65285	23.59654
Senegal	32.33%	92%	5.8373	0.63954	0.16926	2.41645
Sierra Leone	36.77%	84%	61.046	11.03972	2.41645	73.73665
Somalia	7.49%	42%	1.0539	0.03705	0.00981	0.20466
NorthSudan	18.75%	92%	7.041	0.09575	0.02534	0.52893
South Sudan	8.72%	59%	16.9973	0.13999	0.02096	1.99851
Tanzania	44.46%	92%	25.1079	1.99851	0.43745	11.03972
Togo	18.09%	84%	58.9379	7.55115	1.36697	73.73665
Uganda	66.25%	78%	89.748	34.49795	7.55115	190.56627
Zambia	51.51%	78%	26.8963	2.9218	0.63954	16.13999
Zimbabwe	25.70%	89%	2.8336	0.16926	0.05417	0.63954

Additional file 1: Table SM2. Summary of country specific effective coverage of uncomplicated care, vaccination coverage (DTP3), mean and median transmission levels as described by Entomological Inoculation Rate from country levels of prevalence [6]
