# Supplementary material for: The public health impact of malaria vaccine RTS,S in malaria endemic Africa: country-specific predictions using 18 month follow-up Phase III data and simulation models
Source: BMC Med. 2015 Jul 29;13:170. doi: 10.1186/s12916-015-0408-2 (PMC4518512; doi:10.1186/s12916-015-0408-2)
Supplement: Additional file 2 — Supplementary results 1. Additional tables associated with fitting of vaccine properties and country-specific predictions of vaccine impact. [file 12916_2015_408_MOESM2_ESM.pdf]

The public health impact of malaria vaccine RTS,S in malaria endemic Africa: country-specific predictions using 18 month follow-up Phase III data and simulation models

Melissa A Penny*[1,2], Katya Galactionova[1,2], Michael Tarantino[1,2], Marcel Tanner[1,2], Thomas A Smith[1,2]

[1] Swiss Tropical and Public Health Institute, Basel, Switzerland
[2] University of Basel, Basel, Switzerland

* Corresponding Author:
Melissa A Penny
Department of Epidemiology and Public Health
Swiss Tropical and Public Health Institute
Socinstrasse 57, 4051 Basel, Switzerland
email: melissa.penny@unibas.ch

Additional File 2 – Supplementary Results 1 - additional results, plots and tables associated with fitting of vaccine properties and public health impact of RTS,S

RTS,S vaccine properties determined from Phase III clinical trial data

Discussion of best fitting models from Bayesian MCMC fit to clinical data

For each of the data sets from RTS,S Phase III 18 month follow-up [1] — EPI cohort, 5–17 month cohort, or both cohorts combined — we compared the fit of a series of different vaccine profiles (Table S1). When fitting to 6–12 weeks cohort (EPI) alone, the model with lowest DIC assumed site–specific incidence parameters and a vaccine half-life of 1 year (model 8 in Table S1). This was closely followed by two models whose differences in DIC were less than 10; one assuming site–specific incidence and a vaccine half-life of 3 years (model 11), and another assuming site–specific incidence with vaccine half-life estimated as an additional parameter (model 17) .

For the 5–17 month cohort (Table S1) the best fitting model assumed a half-life of 1 year and a common incidence rate across sites (model 1 in Table S1). This was closely followed by two models whose differences in DIC were less than 10; one estimating a common incidence rate across sites and assuming a half-life of 3 years (model 4) and another fitting both common incidence across sites as well as vaccine half-life (estimated to be 2.5 years) (model 13).

When fitting to both cohorts simultaneously and with an adjusted transmission assumption (ii) (Table S1) the best fitting models with lowest DIC estimated site–specific incidence, and either assumed a vaccine half-life of 1 year (model 9) or fit vaccine half life (model 18; estimated half-life approximately 1 year.)

Comparing DIC for the same model between MAP transmission assumption (i) (results not shown) and the adjusted transmission assumption (ii) (Table S1) for all models, the adjusted transmission assumption models had lower DIC, implying a better fit to the data.

In general, better fits were obtained when assuming a half-life of one year compared with three years. And when fitting for vaccine half-life, assuming a common site variation in incidence resulted in much larger estimated means over 2 years compared with approximately 1 year when fitting for site–specific variation.

Posterior distributions of predicted vaccine properties

The Bayesian MCMC fitting estimated high initial efficacy against infection for the 5–17 month cohort (see posterior distributions for models with lowest DIC; Figure S1) but a minimal difference in the posteriors between models fitting site–specific variation in incidence compared to those assuming a common value, except when half-life is assumed to be 3 years. In the case of assuming half-life is three years, the mean initial efficacy is predicted to be lower compared to model results when assuming a half-life of 1 year.

For the EPI cohort, the predicted mean initial efficacy against infection is lower than the 5–17 month cohort and the posterior distributions on efficacy against infection (Figure S2) are much wider than those predicted for the 5–17 month cohort. Narrower distributions are obtained when a common site variation in incidence is fit for. Not surprisingly, much lower mean initial efficacy is predicted for models assuming a half-life of three years.

Investigations of decay shapes of efficacy against infection: further analysis and effect on predicted clinical efficacy.

All fitted models assumed exponential decay of efficacy against infection since the decay shape parameter (k) of the Weibull curve makes little difference to the best fitting curve for the first part of the follow-up period, with predicted temporal patterns of clinical efficacy very similar for different decay shapes of efficacy (exponential, k=0.5 and k= 4 of the Weibull distribution). However there are substantial differences between these different curves for the time points two years or longer after vaccination. Longer follow-up data will allow us to investigate with more certainty if the functional form of the decay in efficacy against infection is non-exponential.

Supplementary Tables

Additional file 2: Table S1. Bayesian MCMC Fitted model summary to Phase III data

Model	Dataset	DIC	Deviance	pD	Efficacy against infection EPI (%) (95% CI)	Efficacy against infection 5–17 month(%) (95% CI)	Mean estimated half-life (year) (95% CI)
**Fit parameters: Site-specific access to care; vaccine efficacy against infection**<br>Assumptions: Half-life of vaccine effect 1 year; common level of within-site variation.<br>Assumptions: Exponential decay of vaccine efficacy.							
1*	5–17 month	135.7	128.5	7.2	-	79.1 (61.9,84.8)	fixed (1)
2	6–12 weeks cohort	129.3	121.6	7.7	77.1 (57.2,84.8)	-	fixed (1)
3	Both	248.5	241.1	7.4	72.4 (44.4,84.6)	79.8 (64.3,84.9)	fixed (1)
**Fit parameters: Site-specific access to care; vaccine efficacy against infection**<br>Assumptions: Half-life of vaccine effect 3 years; common level of within-site variation<br>Assumptions: Exponential decay of vaccine efficacy.							
4*	5–17 month	140.7	133.5	7.2	-	75.7 (56.3,84.7)	fixed (3)
5	EPI cohort	130.5	122.8	7.7	72.1 (50.6,84.5)	-	fixed (3)
6	Both	255.1	247	8.1	65.4 (40.3,83)	76.4 (59.4,84.5)	fixed (3)
**Fit parameters: Site-specific access to care; vaccine efficacy against infection**<br>**Fit parameters: Site-specific level of within-site variation (in disease rate)**<br>Assumptions: Half-life of vaccine effect 1 year; Exponential decay of vaccine efficacy.							
7	5–17 month	161.5	105.1	56.4	-	79.3 (65,84.8)	fixed (1)
8*	EPI cohort	115.8	69.3	46.5	66.9 (48.7,81.4)	-	fixed (1)
9*	Both	182.4	159.8	22.6	64.1 (40.9,81)	80 (69.8,84.8)	fixed (1)
**Fit parameters: Site-specific access to care; vaccine efficacy against infection**<br>**Fit parameters: Site-specific level of within-site variation (in disease rate)**<br>Assumptions: Half-life of vaccine effect 3 years; Exponential decay of vaccine efficacy.							
10	5–17 month	169.4	122	47.4	-	66 (42.8,82.3)	fixed (3)
11*	EPI cohort	121.8	83.1	38.7	45.1 (25.9,64.2)	-	fixed (3)
12	Both	211.4	186.4	25	44.2 (25.4,62.1)	63 (47,77)	fixed (3)
**Fit parameters: Site-specific access to care; vaccine efficacy against infection**<br>**Fit parameters: common level of within-site variation; Half-life of vaccine**<br>Assumptions: Exponential decay of vaccine efficacy.							
13*	5–17 month	139.1	132	7.2	-	76.3 (56.5,84.7)	2.53 (1.06,4.78)
14	EPI cohort	129.8	122.3	7.5	72.3 (49.3,84.3)	-	2.86 (1.11,4.86)
15	Both	253.3	244.9	8.4	69.7 (42.7,84.1)	78.5 (63,84.9)	2.08 (1.04,4.45)
**Fit parameters: Site-specific access to care; vaccine efficacy against infection**<br>**Fit parameters: Site-specific level of within-site variation; Half-life of vaccine**<br>Assumptions: Exponential decay of vaccine efficacy.							
16	5–17 month	153.5	111	42.5	-	75.5 (55.5,84.7)	1.57 (1.01,4.07)
17*	EPI cohort	125.2	74.6	50.6	59.1 (31.9,78.2)	-	1.51 (1.01,4.41)
18*	Both	187.9	163.9	24	62.7 (39.5,80.3)	79.2 (67.3,84.8)	1.12 (1,1.43)

Summary of models fitted to the time- and site–specific Phase III data to estimate vaccine properties. Estimated parameter values and model deviance and DIC included for fits assuming adjusted site exposure transmission assumption (ii). Models indicated by $*$ are those with lowest DIC for each of the fitting data sets (EPI cohort, 5–17 month cohort or both).

Supplementary Figures

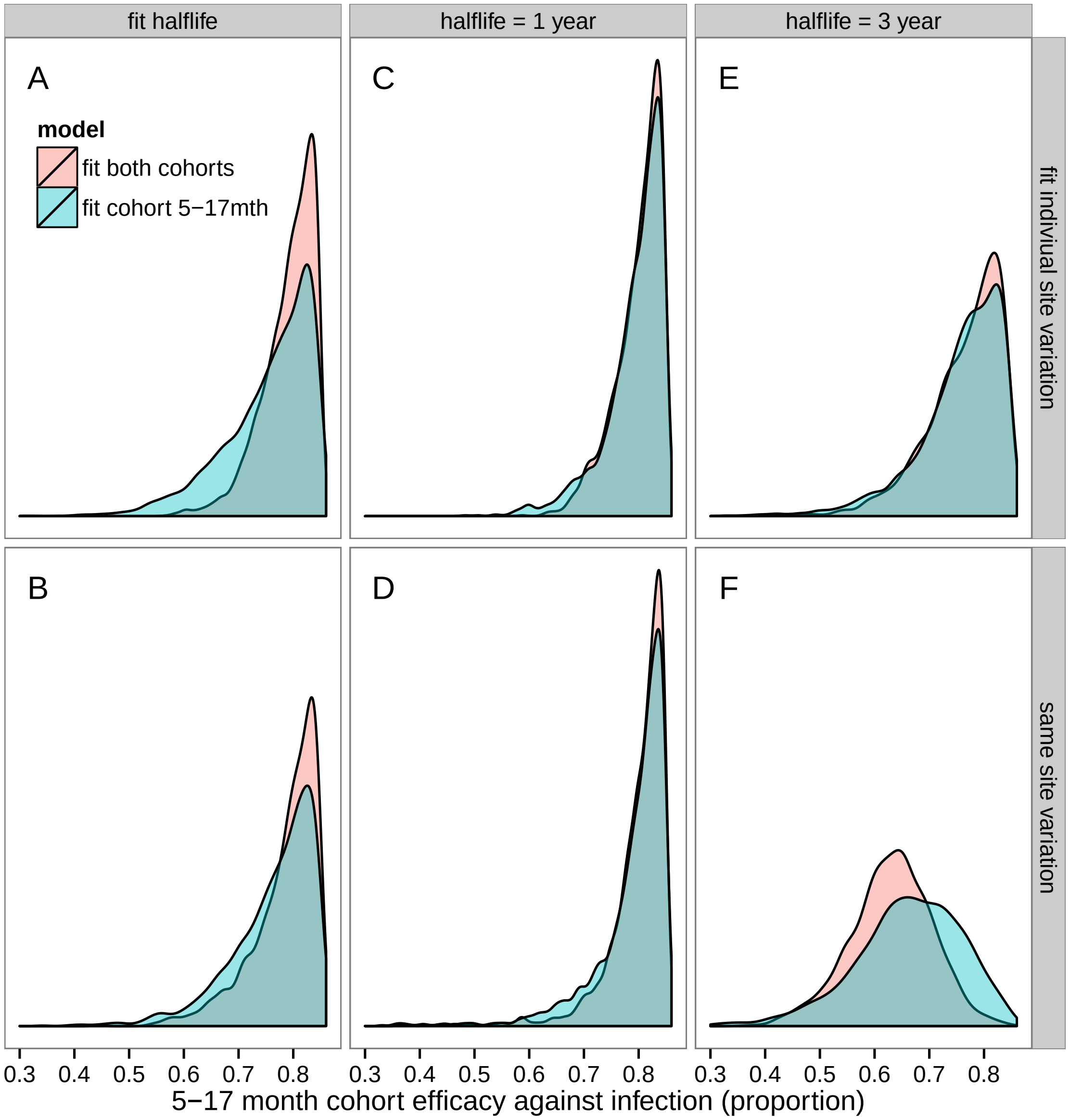

Additional file 2: Figure S1. Posterior distributions of initial efficacy against infection for the 5–17 month cohort. Posterior distributions of efficacy against infection for the 5–17 month cohort for models fitted with the adjusted transmission assumptions (ii). Panels A,C and E show fits when site–specific variation in incidence is fitted, Panels B, D and F show fits when common site–specific variation is fitted. Panels A and B fit for half-life, C and D assume half-life of 1 year and panels E and F assume a half-life of 3 years. In each panel the distribution is shown for efficacy when fitting for both cohorts(rose) or 5–17 month cohort alone (blue).

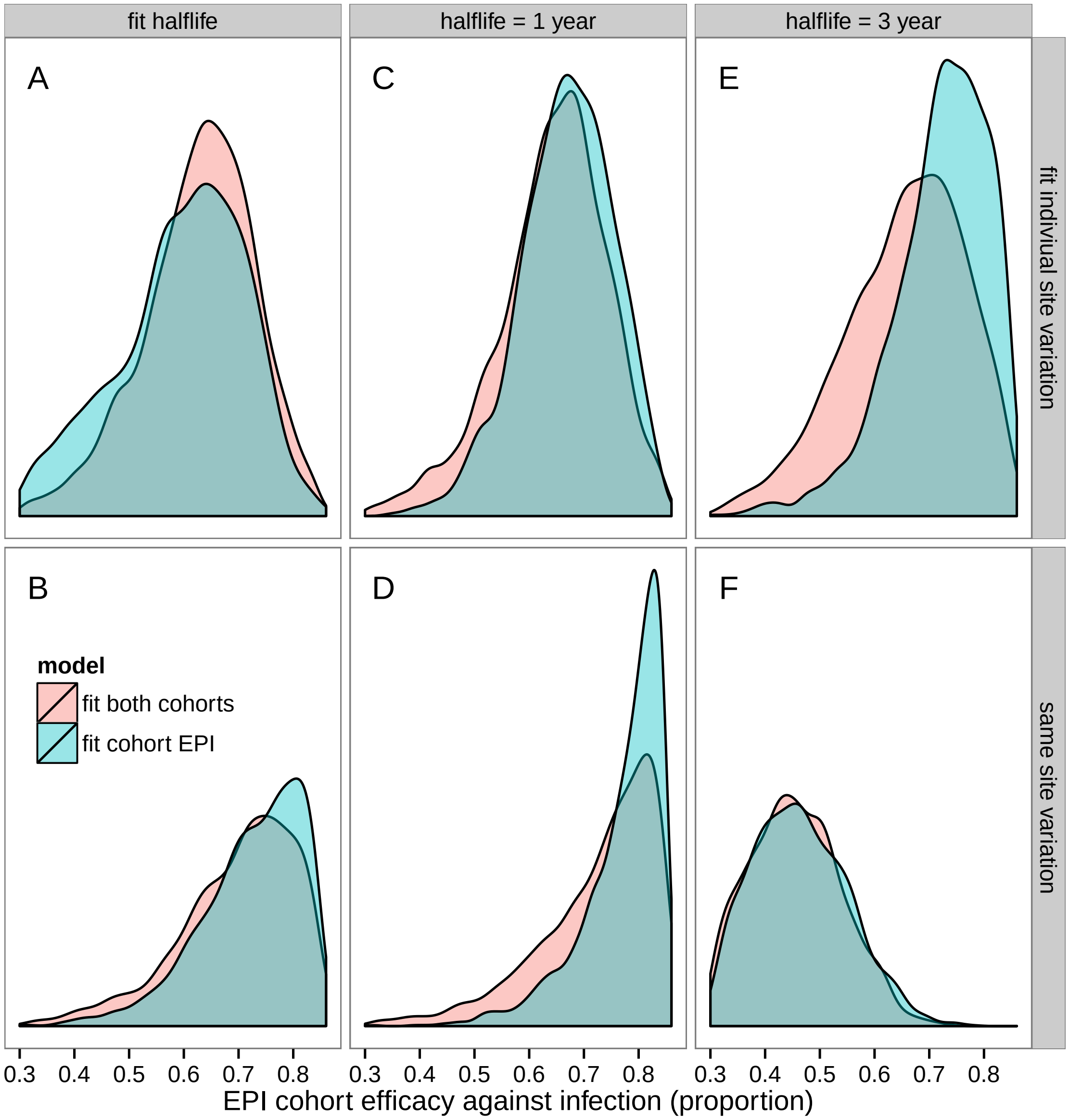

Additional file 2: Figure S2. Posterior distributions of initial efficacy against infection for the EPI month cohort. Posterior distributions of efficacy against infection for the EPI for models fitted with the adjusted transmission assumptions (ii). Panels A,C and E show fits when site–specific variation in incidence is fitted, Panels B, D and F show fits when common site–specific variation is fitted. Panels A and B fit for half-life, C and D assume half-life of 1 year and panels E and F assume a half-life of 3 years. In each panel the distribution is shown for efficacy when fitting for both cohorts (rose) or 6–12 weeks cohort (EPI) alone (blue).

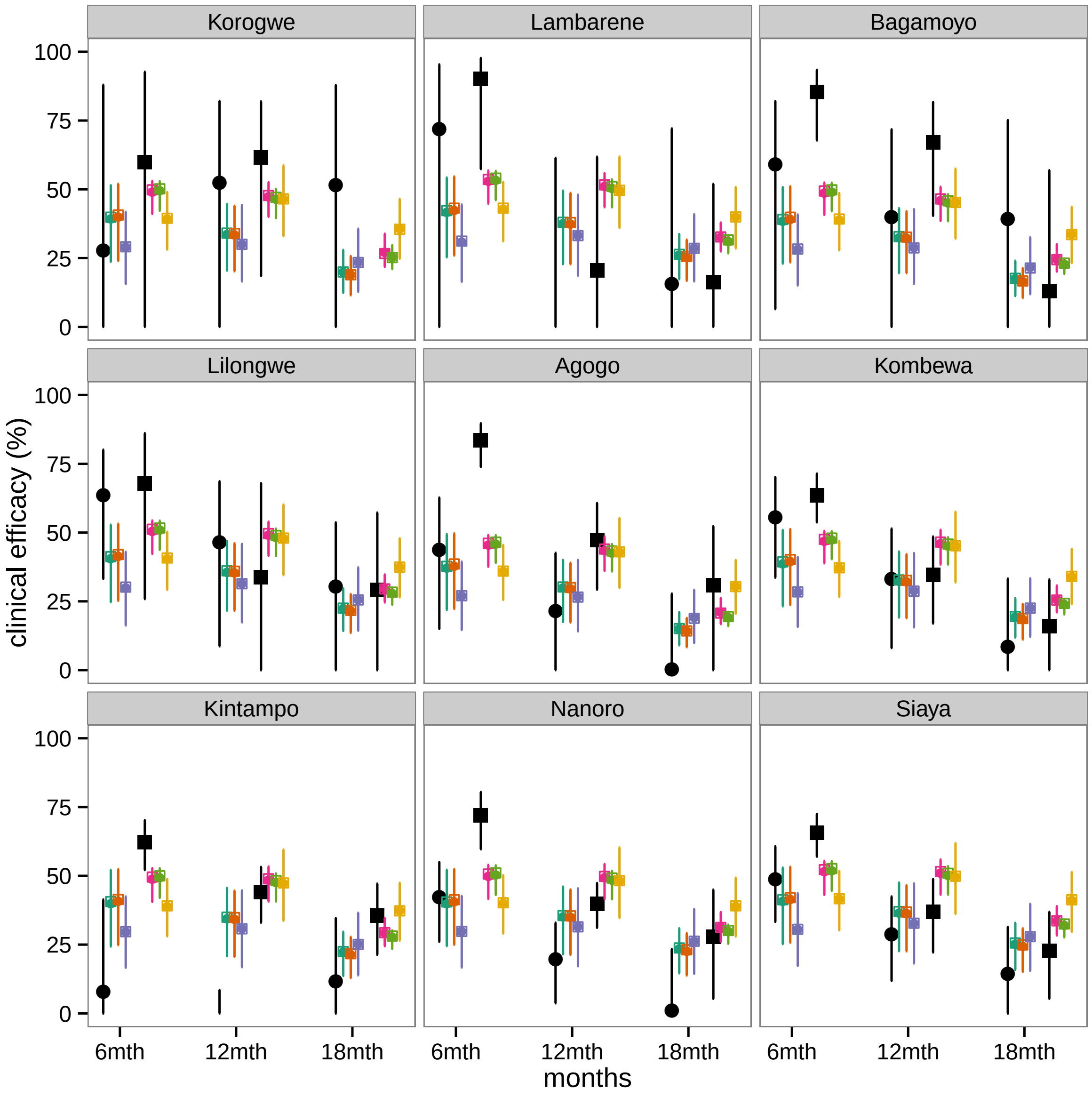

Additional file 2: Figure S3. Clinical efficacy observed and predicted for 18 month followup for 6 monthly periods. Field and predicted estimates of clinical efficacy at each 6 month follow-up for the EPI and 5–17 month cohort, by trial site used in the fitting. Reported efficacy (mean and 95% CI) in the trial site is indicated by black circle for EPI cohort, and black square for the 5–17 month cohort, mean with 95% CI also shown. Prediction estimates (mean and 95% CI) are shown in colour for different fitted models with the adjusted transmission assumptions (ii). Cyan (6–12 weeks cohort (EPI) prediction) and magenta (5–17 month cohort prediction) are fitted predictions for model 18 (fit to both cohorts, fit half-life, and site–specific variation), orange (6–12 weeks cohort (EPI) prediction model 8) and green (5–17 month cohort prediction model 7) are fitted predictions assuming half-life 1 year for models 8 and 7, respectively (fit to corresponding cohort, fit site–specific variation and assume half-life 1 year), purple (EPI month cohort prediction model 11) and yellow (5–17 month cohort prediction model 10) are fitted predictions assuming half-life 3 years for models 10 and 11, respectively (fit to corresponding cohort, fit site–specific variation and assume half-life 3 year)

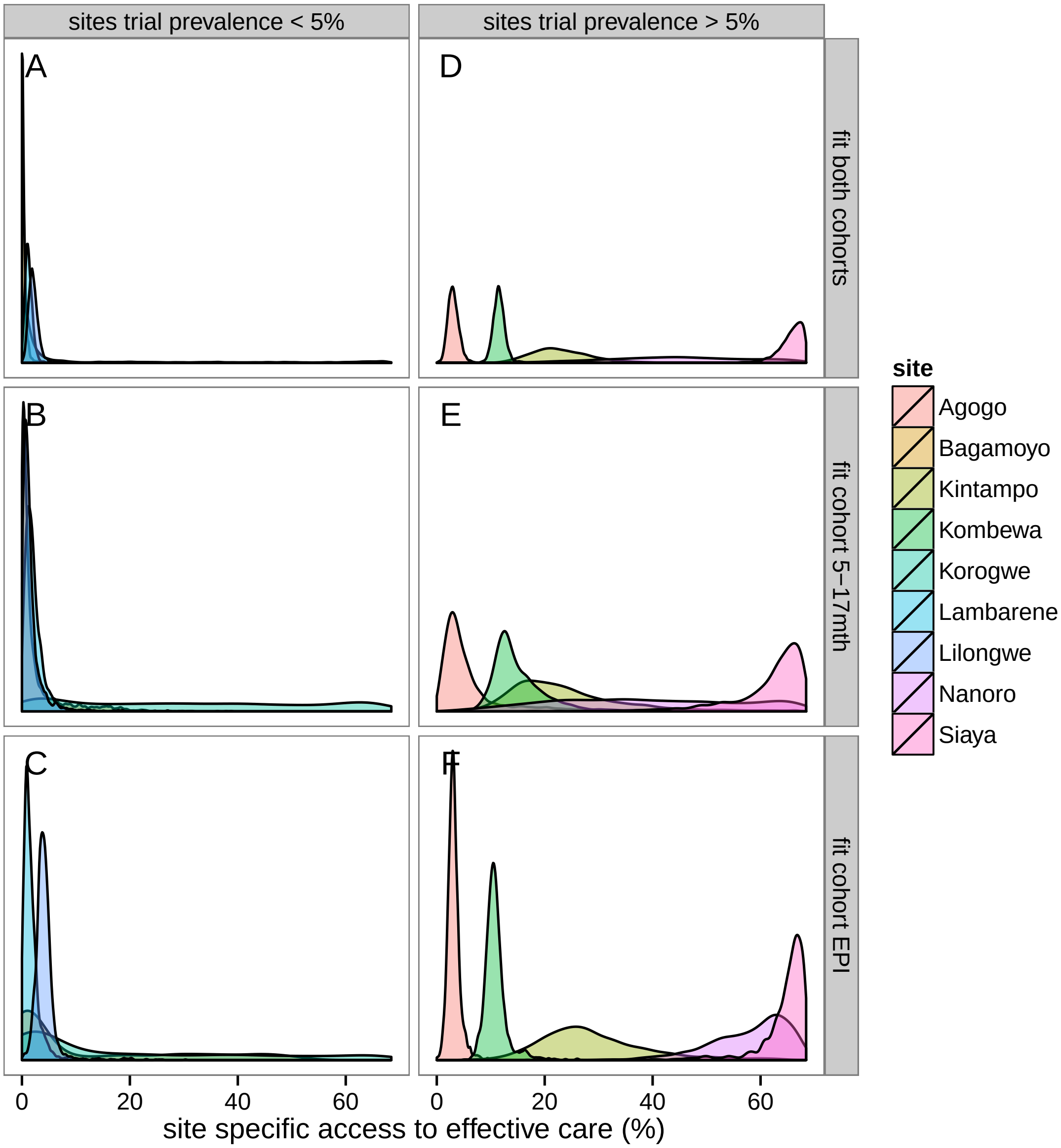

Additional file 2: Figure S4. Posterior distributions of access to effective care for each trial site. Posterior distributions of site–specific access to care for models when half-life is fit with the adjusted transmission assumptions (ii). Panels A, B and C show fits for sites with lowest prevalence from trial data, and Panels D, E and F for the higher prevalence. Panels A and D are when models are fitted to both cohorts, Panels B and E when fit to 5–17 month cohort alone, Panels C and F when fit to 6–12 weeks cohort (EPI) alone. Colour indicates site, given in the legend.

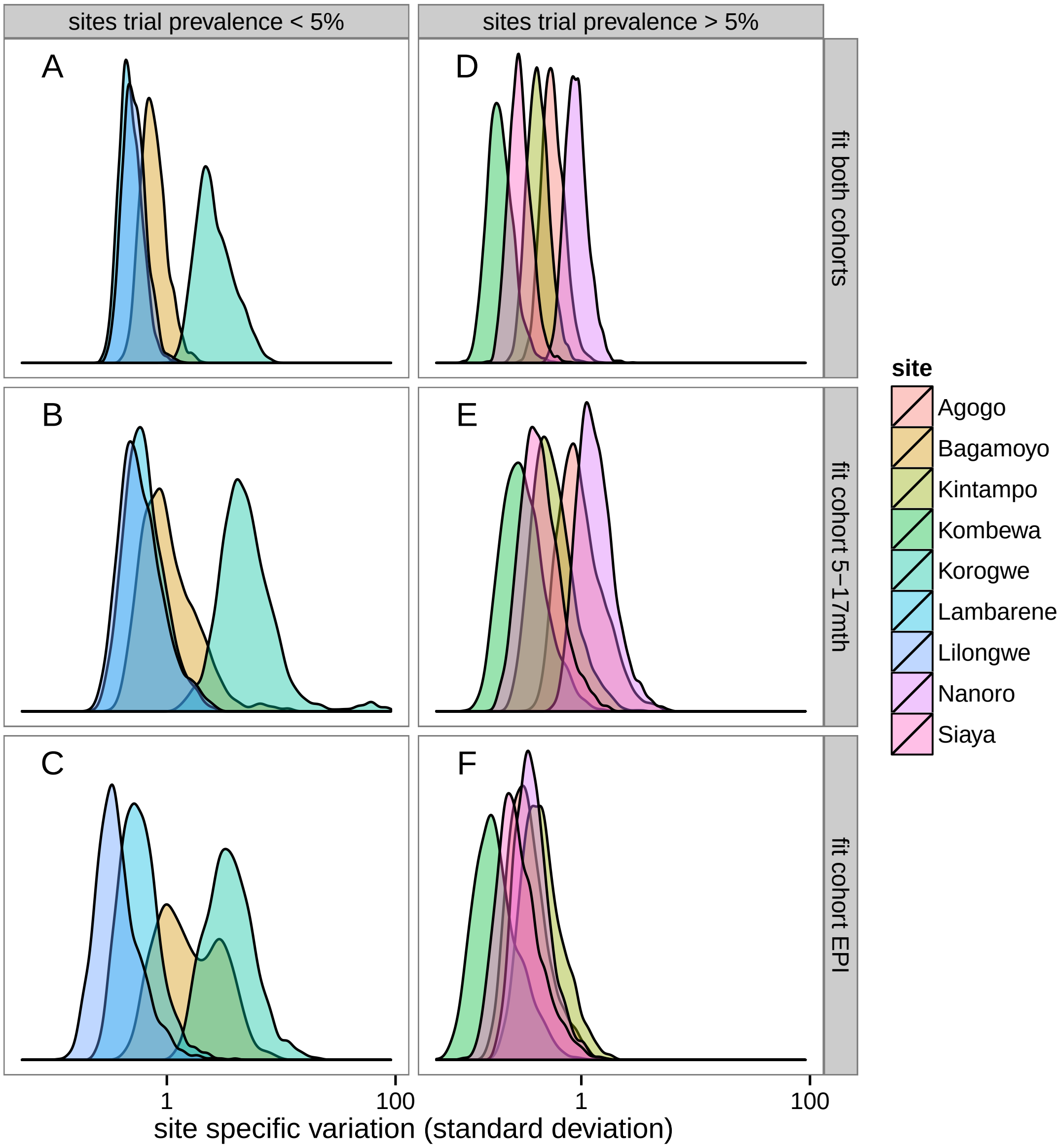

Additional file 2: Figure S5. Posterior distributions of site–specific variation for each trial site. Posterior distributions of site–specific variation in incidence for models when half-life is fit with the adjusted transmission assumptions (ii). Panels A, B and C show fits for sites with lowest prevalence from trial data, and Panels D, E and F for the higher prevalence. Panels A and D are when models are fitted to both cohorts, Panels B and E when fit to 5–17 month cohort alone, Panels C and F when fit to 6–12 weeks cohort (EPI) alone. Colour indicates site, given in the legend.

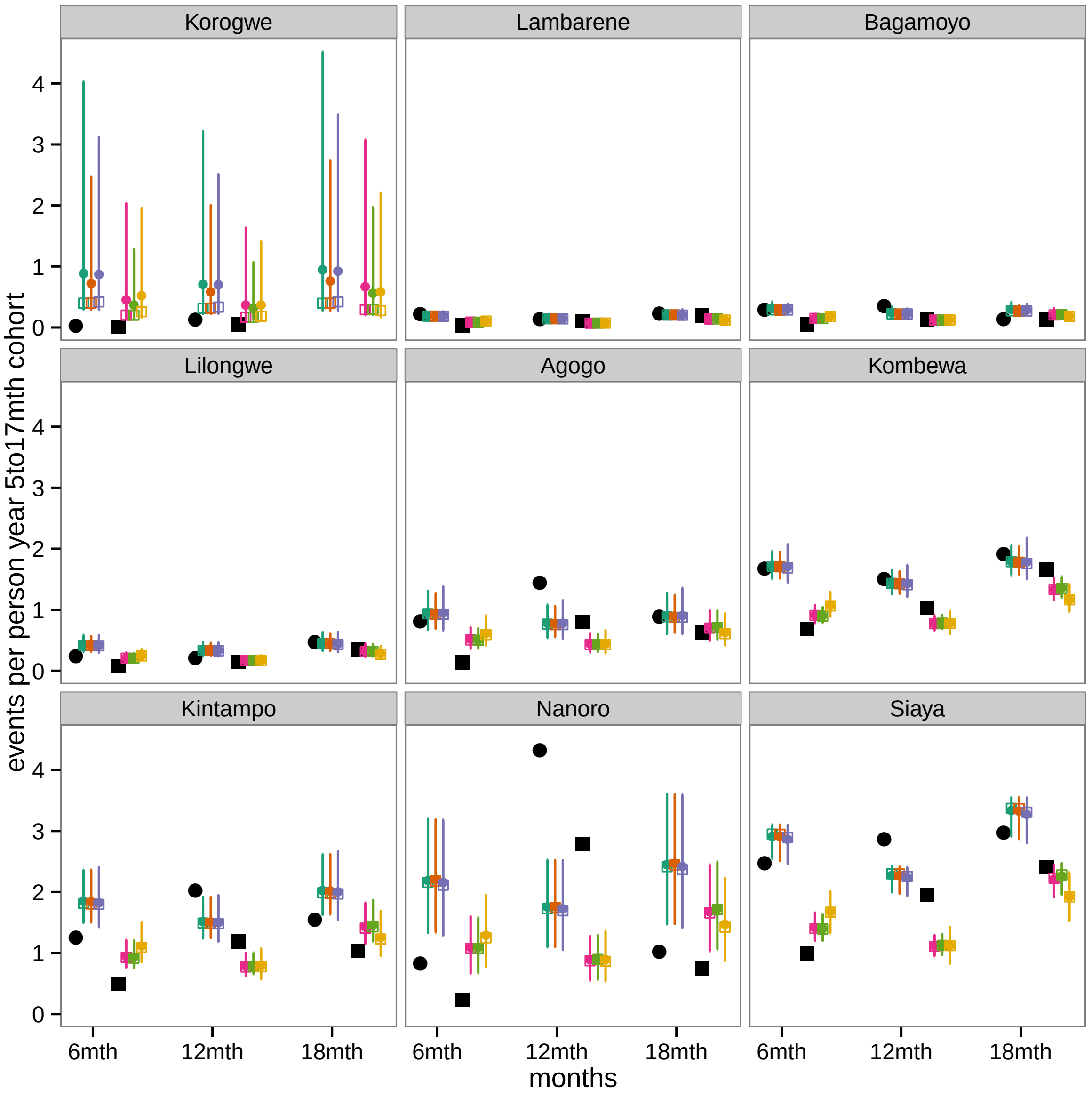

Additional file 2: Figure S6. Clinical incidence observed and predicted for 18 month followup in the 5–17 month cohort. Field and predicted estimates of incidence per person year at each 6 month follow-up for the 5–17 month cohort, by trial site used in the fitting. Reported cases as incidence per person year in the trial site is indicated by black circle for control group and black square for vaccinated cohort. Prediction estimates (mean and 95% CI) are shown in colour for different fitted models with the adjusted transmission assumptions (ii). Cyan (control cohort prediction) and magenta (vaccinated month cohort prediction) are fitted predictions for model 18 (fit to both cohorts, fit half-life, and site–specific variation), orange (control cohort prediction) and green (vaccinated cohort prediction) are fitted predictions for model 8 assuming half-life 1 year (fit to 5–17 month cohort, fit site–specific variation and assume half-life 1 year), purple (control cohort prediction) and yellow (vaccinated cohort prediction) are fitted predictions for model 11 assuming half-life 3 years (fit to 5–17month cohort, fit site–specific variation and assume half-life 3 year)

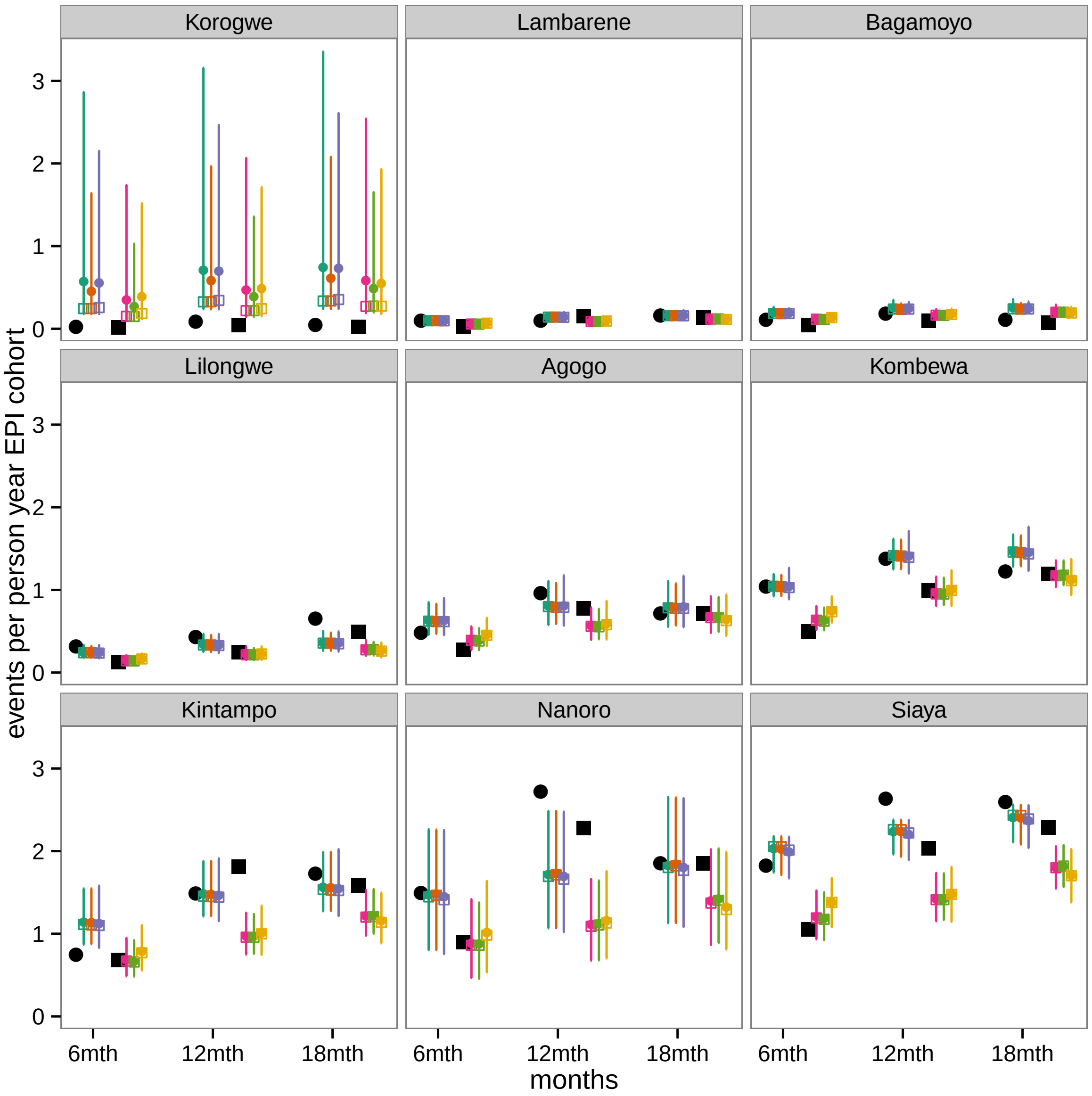

Additional file 2: Figure S7. Clinical incidence observed and predicted for 18 month followup in the EPI cohort. Field and predicted estimates of incidence per person year at each 6 month follow-up for the EPI cohort, by trial site used in the fitting. Reported cases as incidence per person year in the trial site is indicated by black circle for control group and black square for vaccinated cohort. Prediction estimates (mean and 95% CI) are shown in colour for different fitted models with the adjusted transmission assumptions (ii). Cyan (control cohort prediction) and magenta (vaccinated month cohort prediction) are fitted predictions for model 18 (fit to both cohorts, fit half-life, and site–specific variation), orange (control cohort prediction) and green (vaccinated cohort prediction) are fitted predictions for model 8 assuming half-life 1 year (fit to EPI cohort, fit site–specific variation and assume half-life 1 year), purple (control cohort prediction) and yellow (vaccinated cohort prediction) are fitted predictions for model 11 assuming half-life 3 years (fit to EPI cohort, fit site–specific variation and assume half-life 3 year)

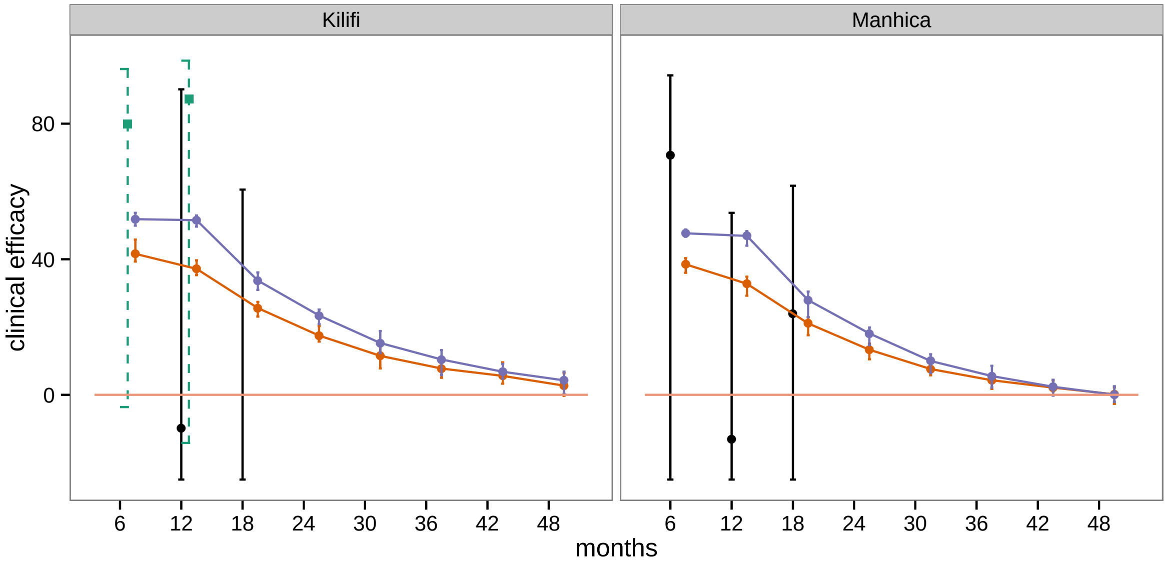

Additional file 2: Figure S8. Predicted clinical efficacy beyond 18 months for EPI and 5–17 month cohort for trials sites not used for the fitting Projections of clinical efficacy by site for the 6–12 weeks cohort (EPI) (orange) and 5–17 month cohort for follow-up longer than 18 months for trials sites Kilifi and Manhica that were not used in the fitting. Predictions are results of assuming vaccine parameters from model 18 (fit for half-life, site–specific variation and to both cohorts) and assuming site levels of exposure from adjusted transmission assumptions (ii). Black indicates mean estimates of trial data with 95% CI for the 6–12 weeks cohort (EPI) and green for 5–17 month cohort, orange the model predictions for 6–12 weeks cohort (EPI) and purple model predictions for the 5–17 month cohort.

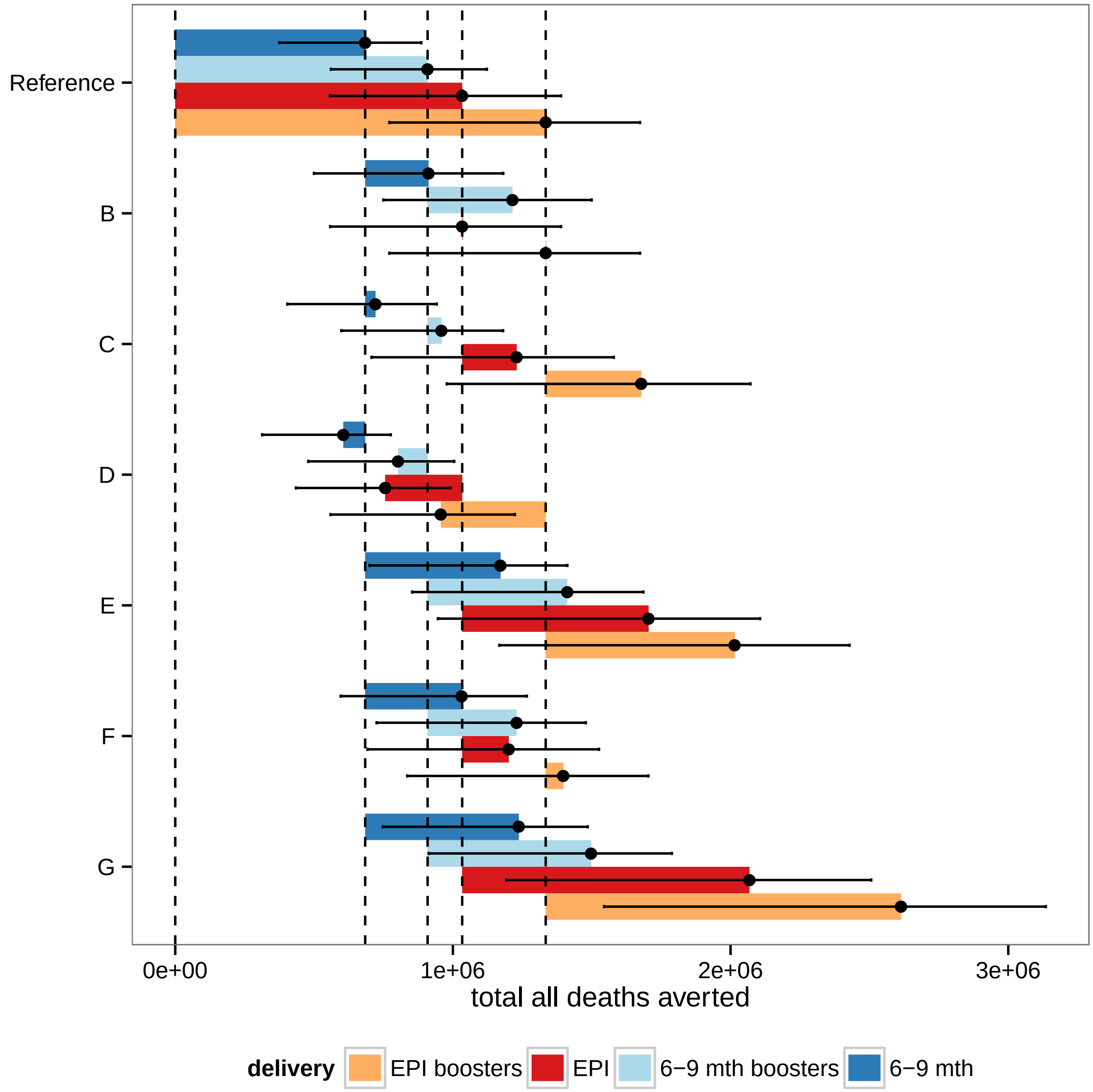

Additional file 2: Figure S9. Predicted cumulative all deaths averted over 10 years for sub-Saharan Africa for each of the four vaccine implementations EPI (6–12 weeks), EPI with boosters, expanded routine and expanded routine with boosters. Predictions of the overall number of all cause deaths averted over ten years, for vaccine and coverage sensitivities B-G (see Table 3 (main text)), for EPI (6–12 weeks) (red), EPI with boosters (orange), expanded routine (dark blue), and expanded routine with booster (light blue). Points correspond to the means of the predictions based on weighted averages over all simulations of the vaccine profile. Vertical lines correspond to the means of the predictions for the reference vaccine profile for each of the four vaccination schedules. Error bars represent the minima and maxima of the predictions based on replication of the simulations with 6 different model variants each with 5 random number seeds.

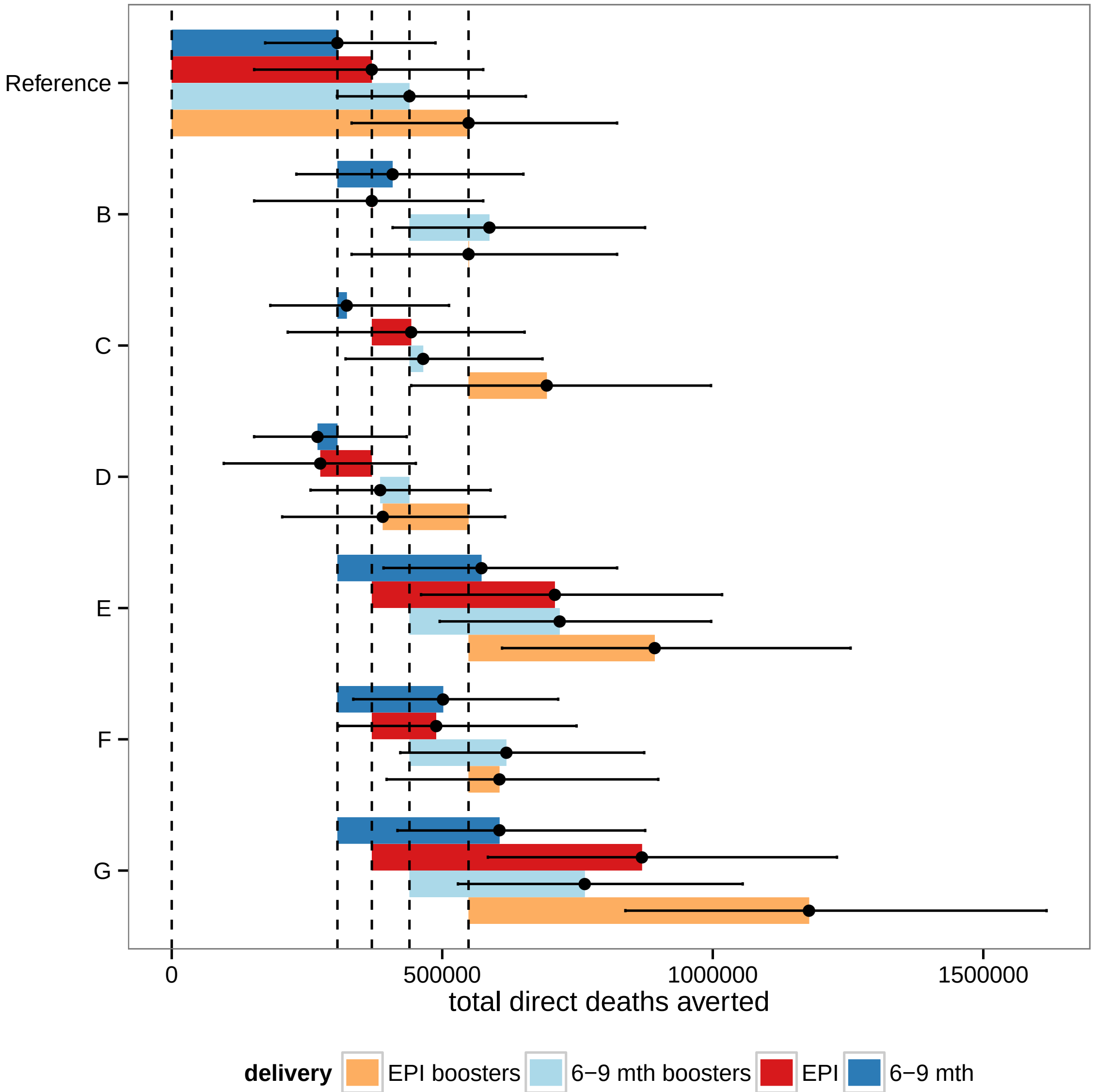

Additional file 2: Figure S10. Predicted cumulative direct deaths averted over 10 years for sub-Saharan Africa for each of the four vaccine implementations EPI (6–12 weeks), EPI with boosters, expanded routine and expanded routine with boosters. Predictions of the overall number of direct malaria deaths averted over ten years, for vaccine and coverage sensitivities B-G (see Table 3 (main text)), for EPI (6–12 weeks) (red), EPI with boosters (orange), expanded routine (dark blue), and expanded routine with booster (light blue). Points correspond to the means of the predictions based on weighted averages over all simulations of the vaccine profile. Vertical lines correspond to the means of the predictions for the reference vaccine profile for each of the four vaccination schedules. Error bars represent the minima and maxima of the predictions based on replication of the simulations with 6 different model variants each with 5 random number seeds.

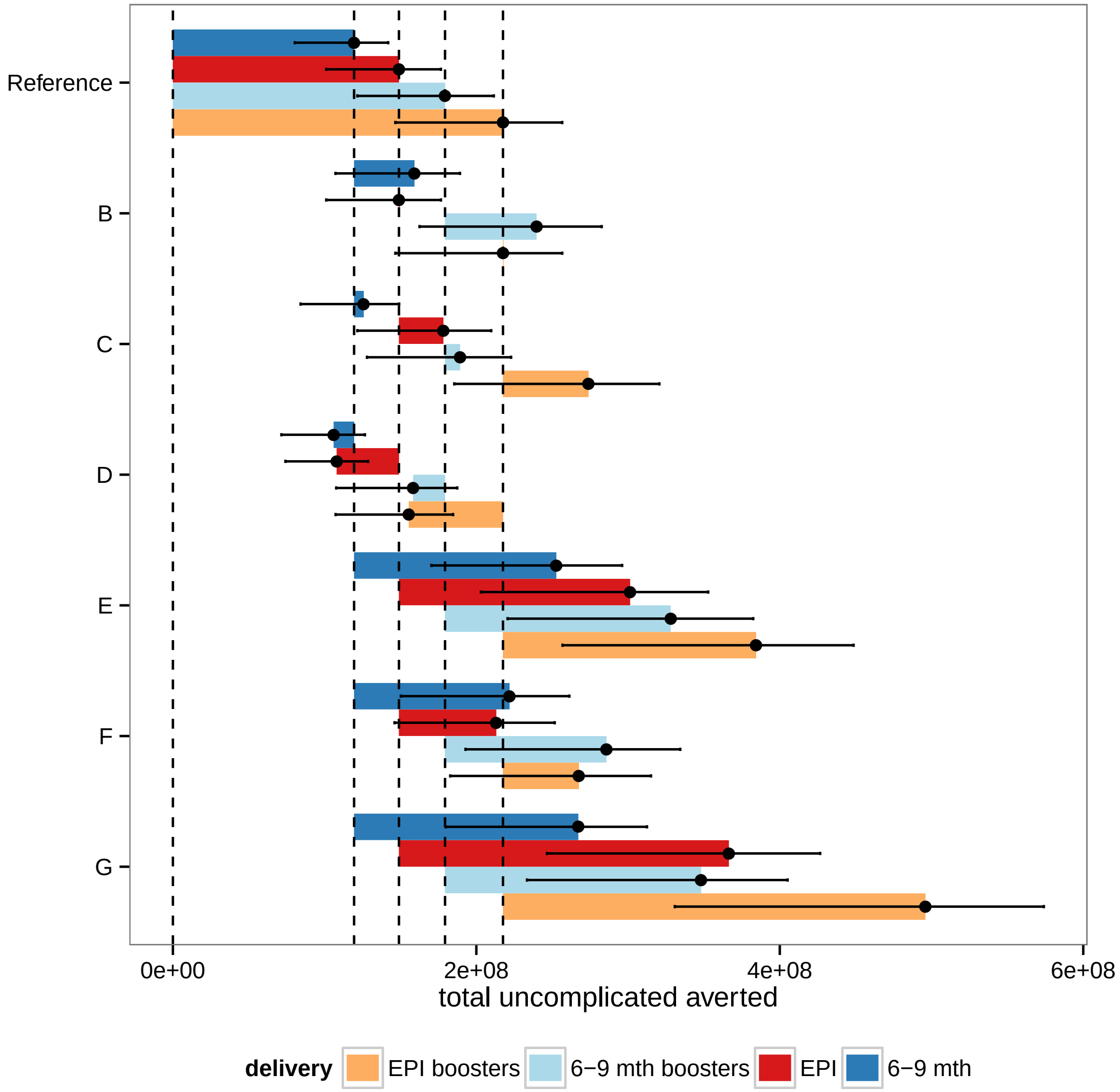

Additional file 2: Figure S11. Predicted cumulative uncomplicated cases averted over 10 years for sub-Saharan Africa for each of the four vaccine implementations EPI (6–12 weeks), EPI with boosters, expanded routine and expanded routine with boosters. Predictions of the overall number of all cause deaths averted over ten years, for vaccine and coverage sensitivities B-G (see Table 3 (main text)), for EPI (6–12 weeks) (red), EPI with boosters (orange), expanded routine (dark blue), and expanded routine with booster (light blue). Points correspond to the means of the predictions based on weighted averages over all simulations of the vaccine profile. Vertical lines correspond to the means of the predictions for the reference vaccine profile for each of the four vaccination schedules. Error bars represent the minima and maxima of the predictions based on replication of the simulations with 6 different model variants each with 5 random number seeds.

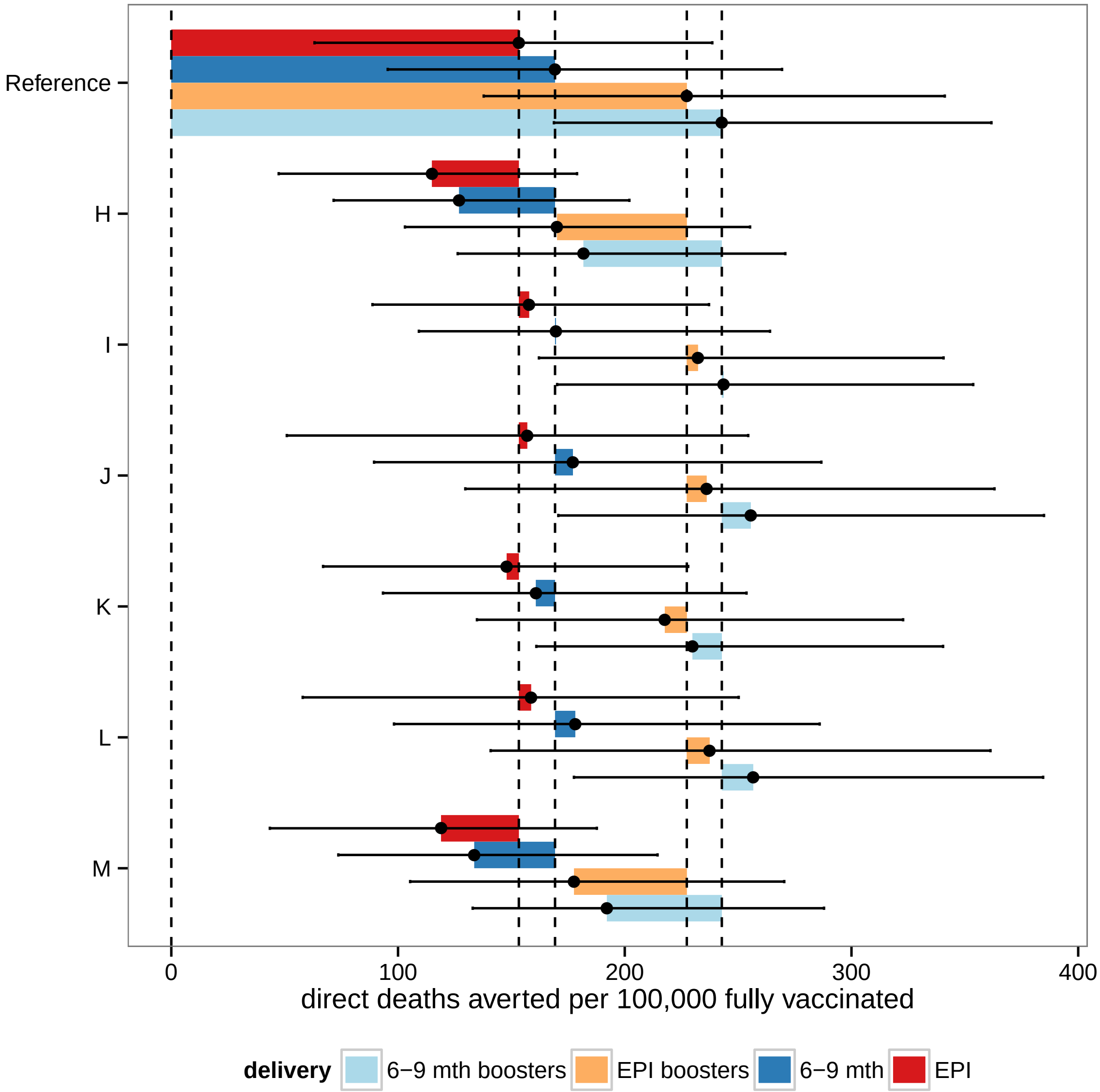

Additional file 2: Figure S12. Predicted cumulative direct deaths averted over 10 years for sub-Saharan Africa for each of the four vaccine implementations EPI (6–12 weeks), EPI with boosters, expanded routine and expanded routine with boosters. Predictions of the overall number of direct malaria deaths averted over ten years, for coverage, transmission and access to effective treatment sensitivities H-M (see Table SM1), for EPI (6–12 weeks) (red), EPI with boosters (orange), expanded routine (dark blue), and expanded routine with booster (light blue). Points correspond to the means of the predictions based on weighted averages over all simulations of the vaccine profile. Vertical lines correspond to the means of the predictions for the reference vaccine profile for each of the four vaccination schedules. Error bars represent the minima and maxima of the predictions based on replication of the simulations with 6 different model variants each with 5 random number seeds.

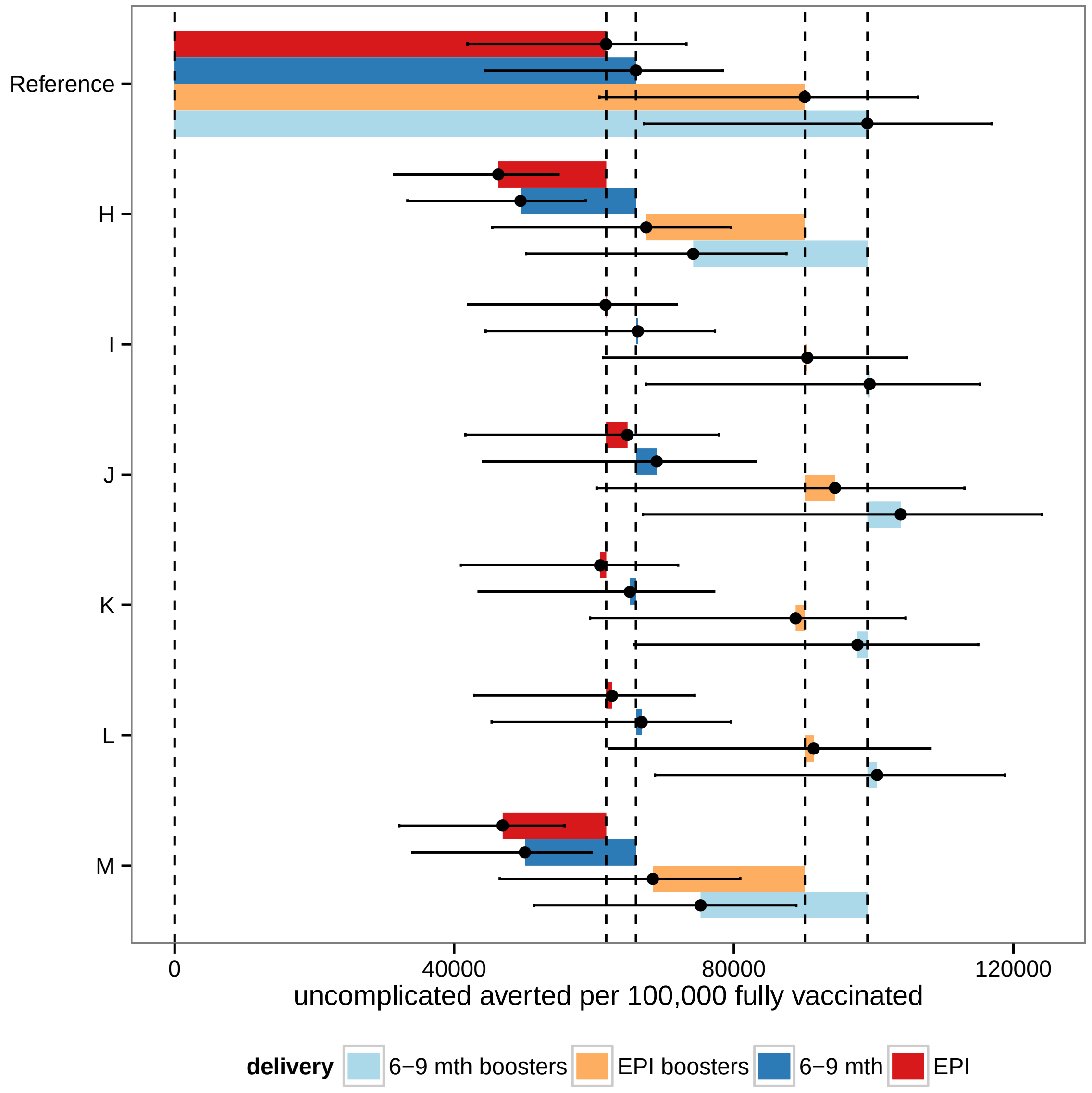

Additional file 2: Figure S13. Predicted cumulative uncomplicated cases averted over 10 years for sub-Saharan Africa for each of the four vaccine implementations EPI (6–12 weeks), EPI with boosters, expanded routine and expanded routine with boosters. Predictions of the overall number of all cause deaths averted over ten years, for coverage, transmission and access to effective treatment sensitvities H-M (see Table SM1), for EPI (6–12 weeks) (red), EPI with boosters (orange), expanded routine (dark blue), and expanded routine with booster (light blue). Points correspond to the means of the predictions based on weighted averages over all simulations of the vaccine profile. Vertical lines correspond to the means of the predictions for the reference vaccine profile for each of the four vaccination schedules. Error bars represent the minima and maxima of the predictions based on replication of the simulations with 6 different model variants each with 5 random number seeds.

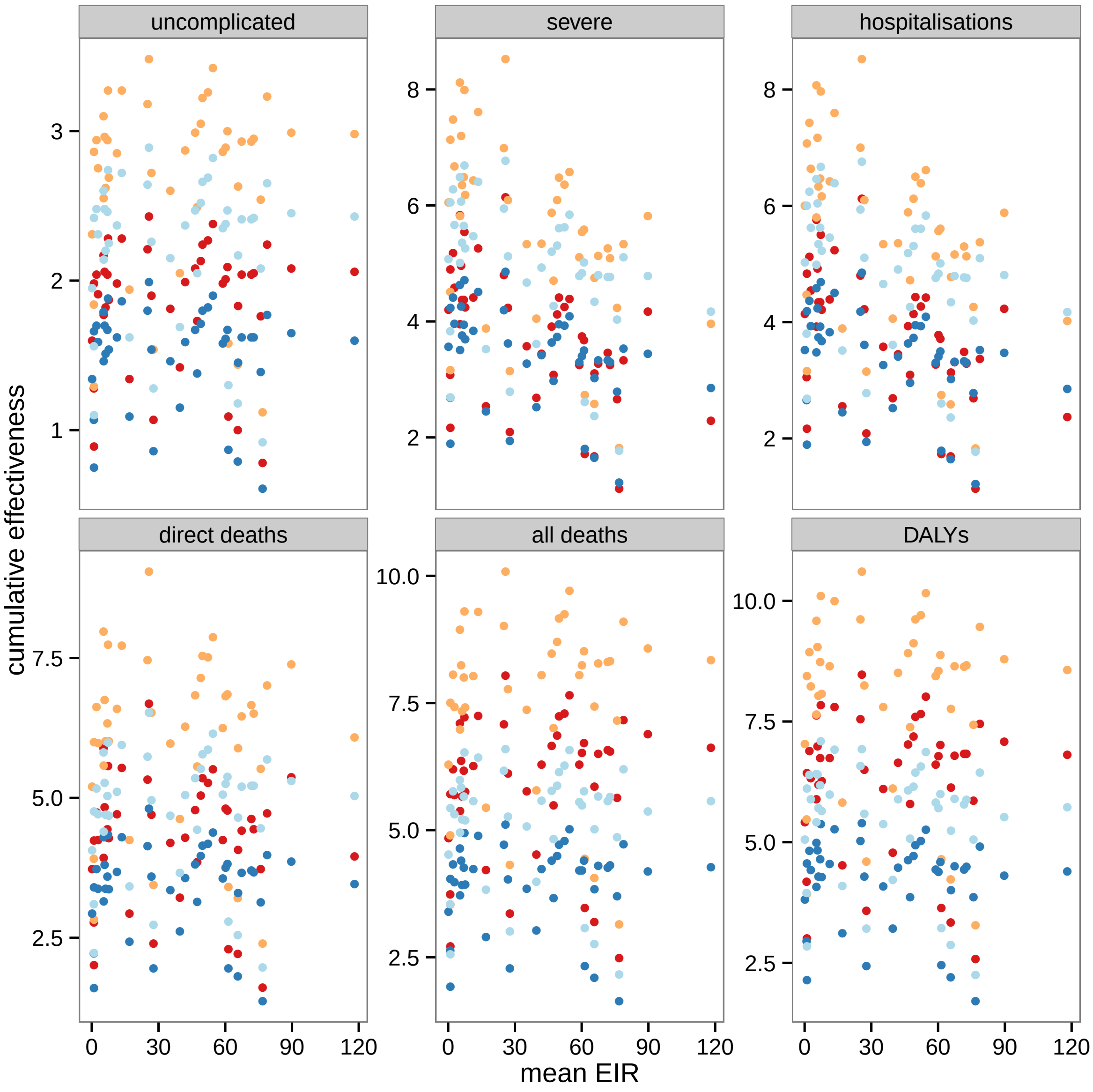

Additional file 2: Figure S14. Mean predicted cumulative effectiveness in terms of events averted over entire population over 10 years by mean level of EIR for each of the 43 sub-Saharan African countries for each of the four vaccine implementations EPI (6–12 weeks), EPI with boosters, expanded routine and expanded routine with boosters. Predictions of the cumulative effectiveness for events averted over all population over ten years, for vaccine reference profile (see Table 3 (main text)) by level of access to effective care. Immunisation strategies are EPI (6–12 weeks) (red), EPI with boosters (orange), expanded routine (dark blue), and expanded routine with booster (light blue). Points correspond to the means of the predictions based on weighted averages over all simulations of the vaccine profile.

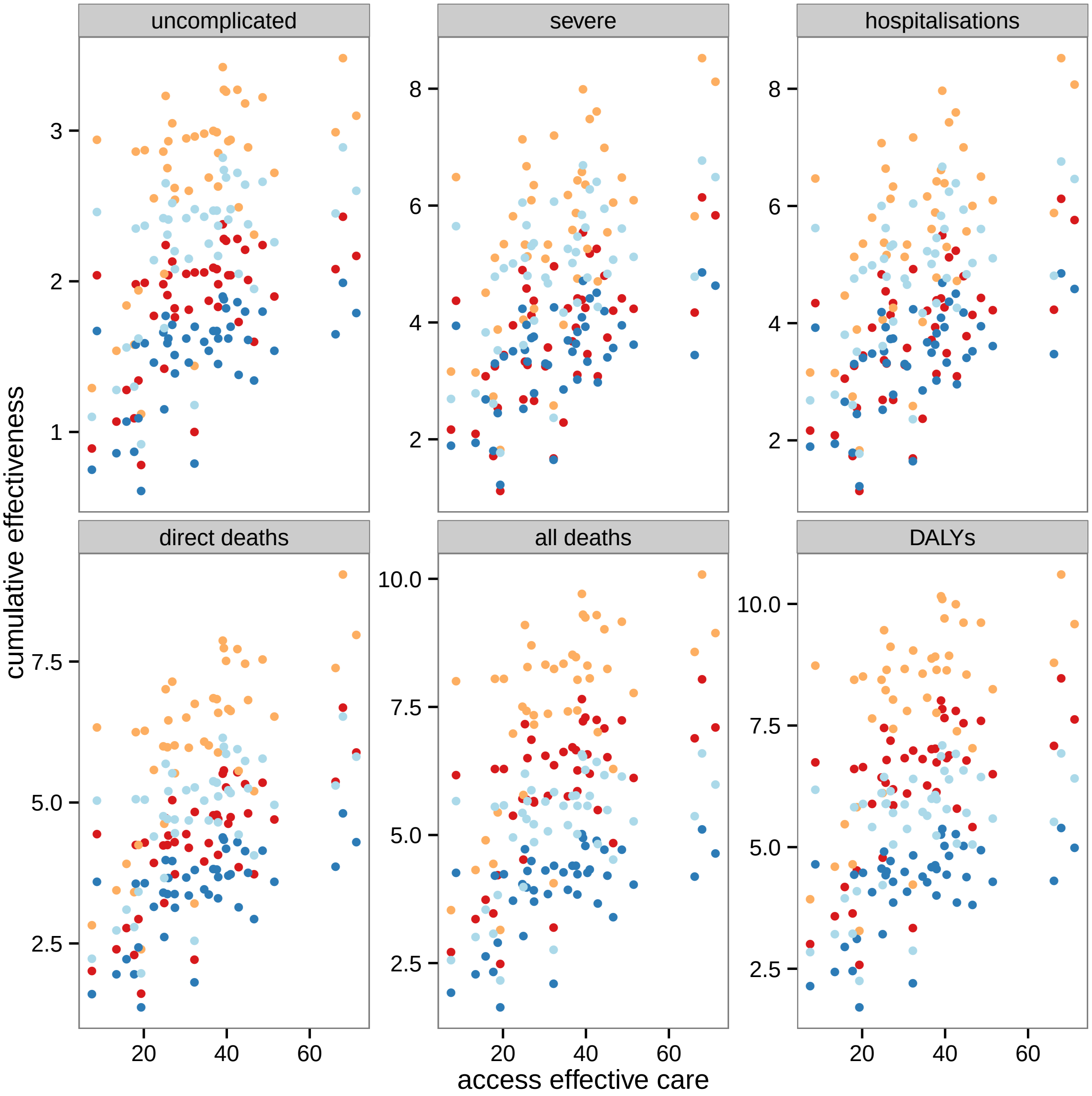

Additional file 2: Figure S15. Mean predicted cumulative effectiveness in terms of events averted over entire population over 10 years by level of access to effective care for each of the 43 sub-Saharan African countries for each of the four vaccine implementations EPI (6–12 weeks), EPI with boosters, expanded routine and expanded routine with boosters. Predictions of the cumulative effectiveness for events averted over all population over ten years, for vaccine reference profile (see Table 3 (main text)) by level of access to effective care. Immunisation strategies are EPI (6–12 weeks) (red), EPI with boosters (orange), expanded routine (dark blue), and expanded routine with booster (light blue). Points correspond to the means of the predictions based on weighted averages over all simulations of the vaccine profile.

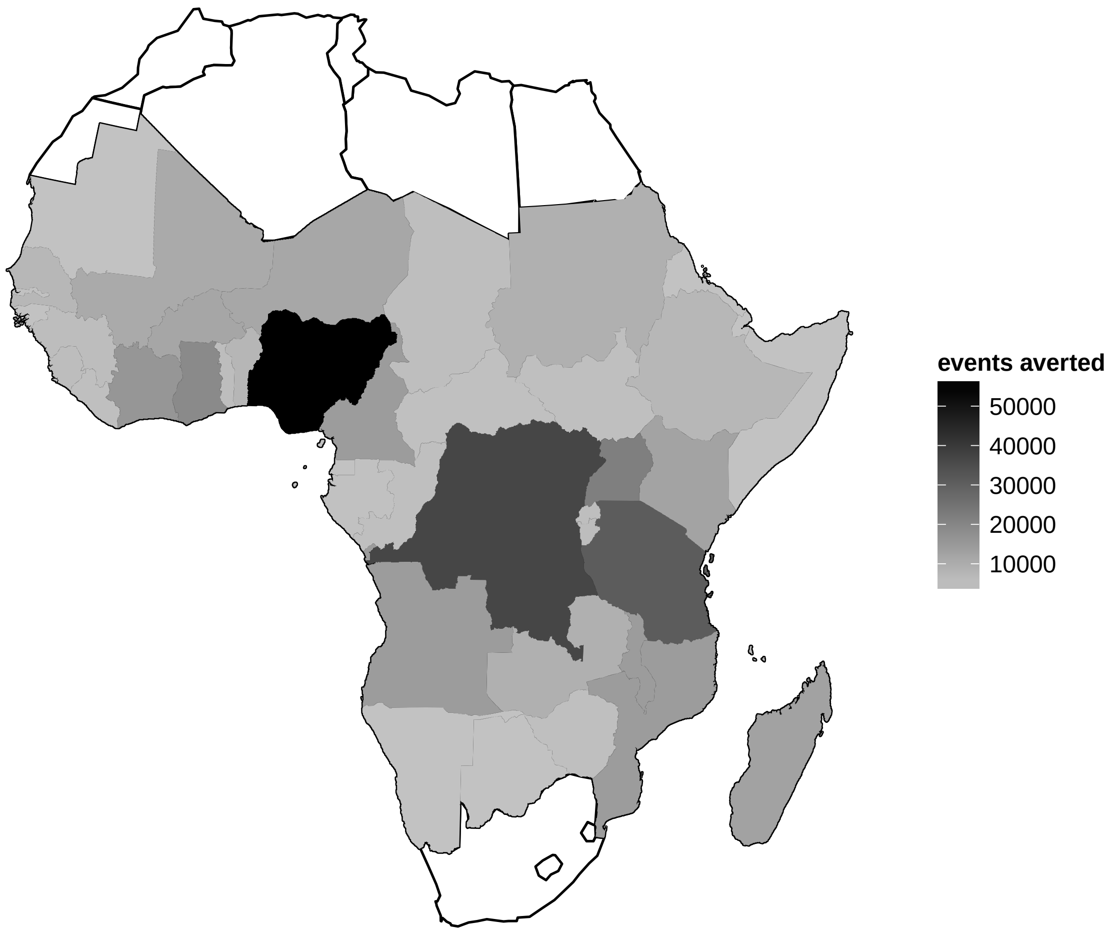

Additional file 2: Figure S16. Mean predicted total direct malaria deaths expected to be averted after 10 years by country for EPI (6–12 weeks) immunisation schedule. Cumulative total direct malaria deaths averted (all ages) by country, cumulative at 10 years post introduction immunising via EPI routine immunisation schedule of 6–12 weeks (vaccination coverage is at DTP3 levels of country immunisation). Total averted is dependent on country population size and underlying level of burden.

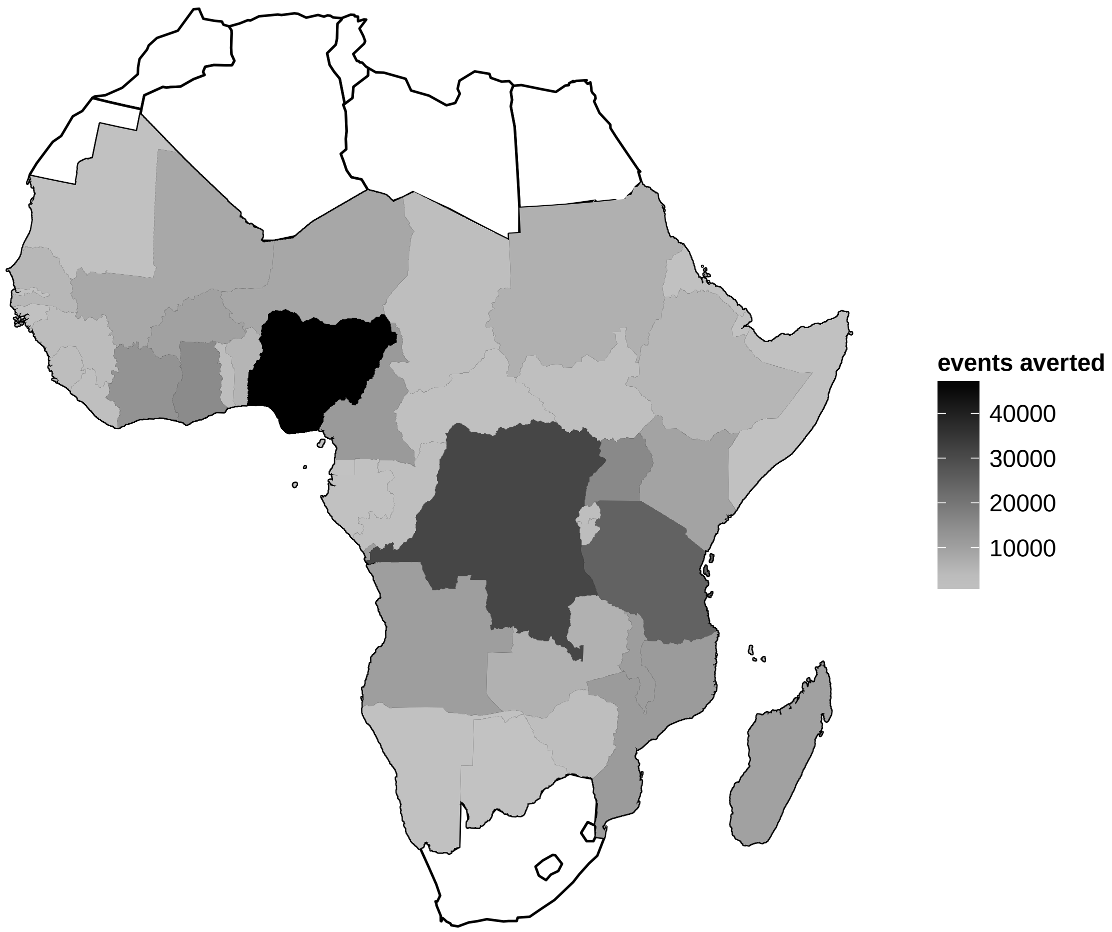

Additional file 2: Figure S17. Mean predicted total direct malaria deaths expected to be averted after 10 years by country for expanded routine (6-9 month) immunisation schedule. Cumulative total direct malaria deaths averted (all ages) by country, cumulative at 10 years post introduction immunising via expanded routine immunisation schedule of 6-9 months (vaccination coverage is at 75% of DTP3 levels of country immunisation). Total averted is dependent on country population size and underlying level of burden.

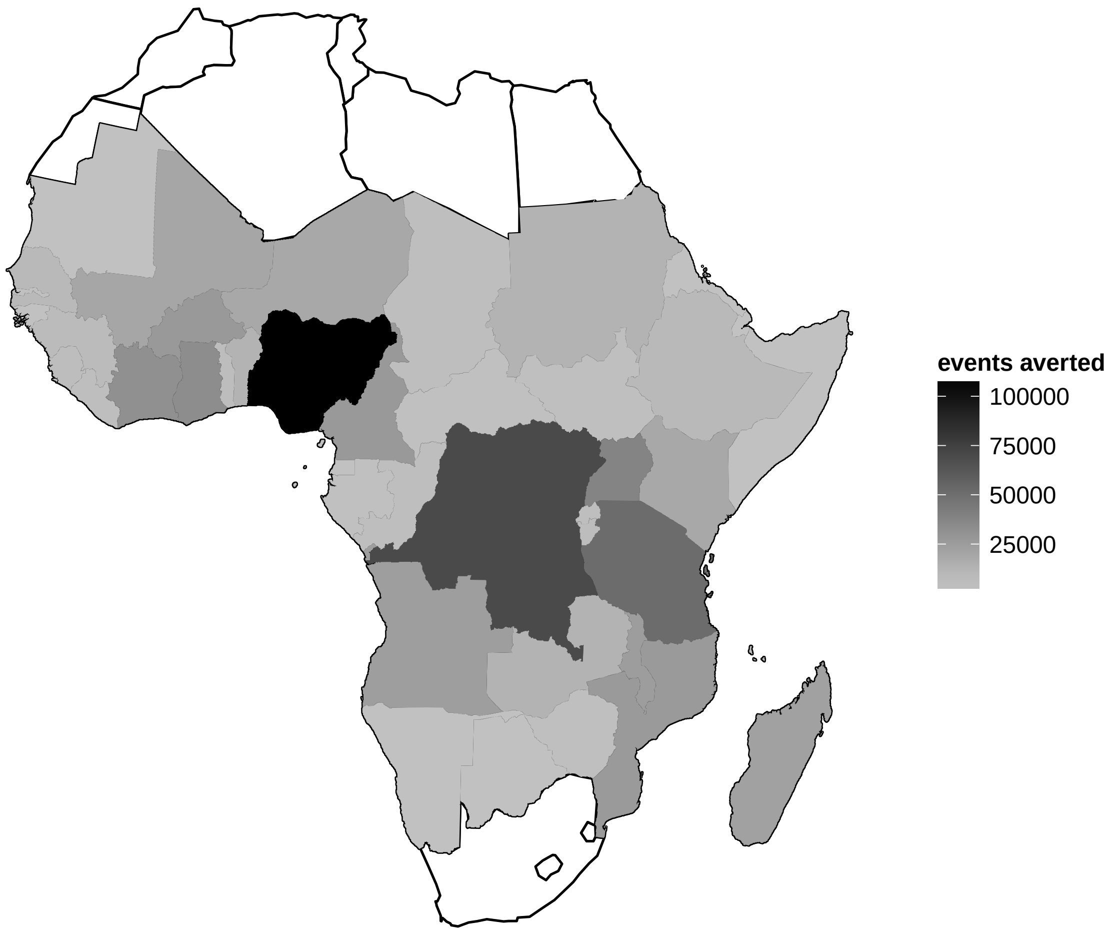

Additional file 2: Figure S18. Mean predicted total all malaria deaths expected to be averted after 10 years by country for expanded routine (6-9 month) immunisation schedule. Cumulative total all malaria deaths averted (all ages) by country, cumulative at 10 years post introduction immunising via expanded routine immunisation schedule of 6-9 months (vaccination coverage is at 75% of DTP3 levels of country immunisation). Total averted is dependent on country population size and underlying level of burden.

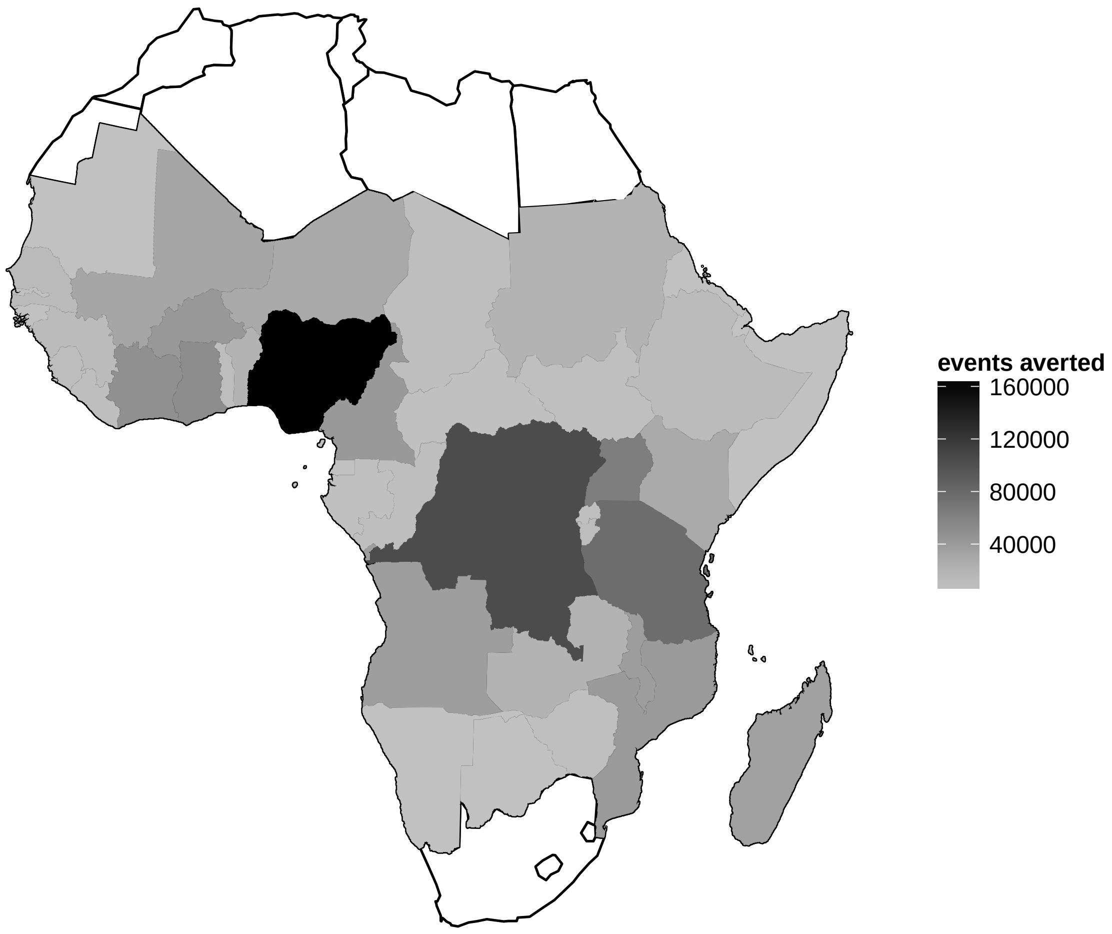

Additional file 2: Figure S19. Mean predicted total all malaria deaths expected to be averted after 10 years by country for EPI (6–12 weeks) immunisation schedule. Cumulative total all malaria deaths averted (all ages) by country, cumulative at 10 years post introduction immunising via EPI routine immunisation schedule of 6–12 weeks (vaccination coverage is at DTP3 levels of country immunisation). Total averted is dependent on country population size and underlying level of burden.

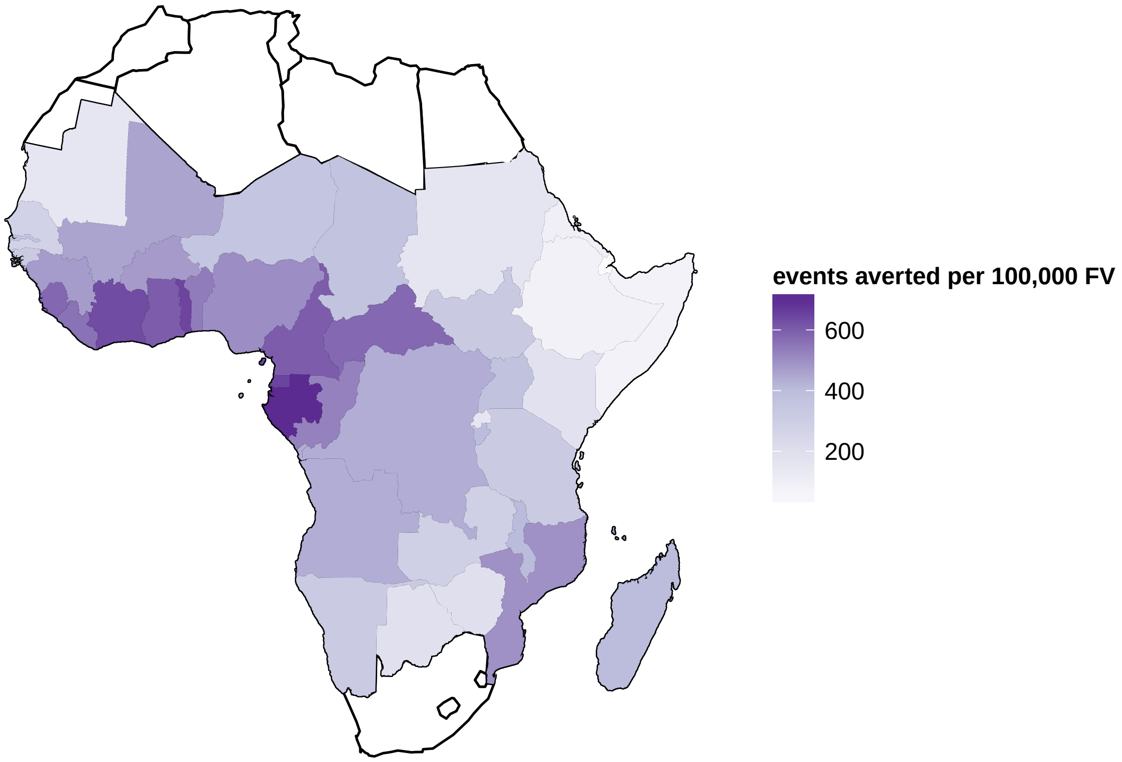

Additional file 2: Figure S20. Mean predicted total all malaria deaths averted per 100,000 fully vaccinated after 10 years by country for expanded routine (6-9 month) immunisation schedule. Cumulative total all malaria deaths averted per 100,000 fully vaccinated by country, cumulative at 10 years post introduction immunising via expanded routine immunisation schedule of 6-9 months (vaccination coverage is at 75% of DTP3 levels of country immunisation).

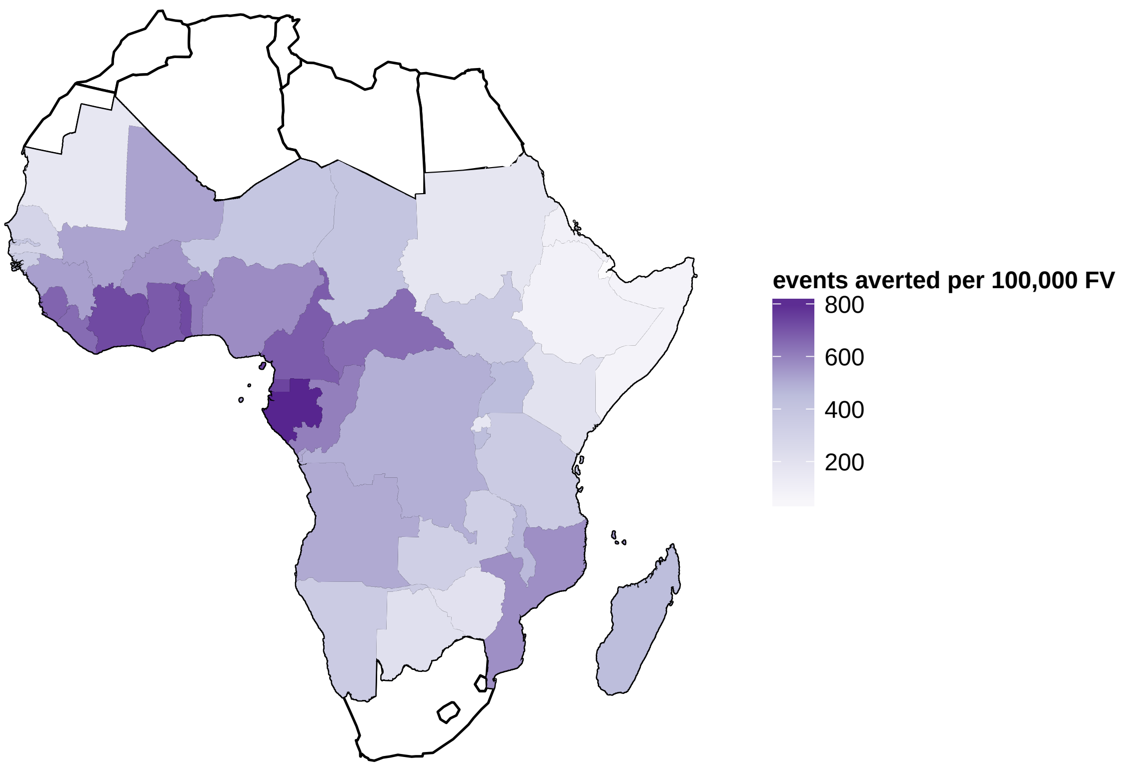

Additional file 2: Figure S21. Mean predicted total all malaria deaths averted per 100,000 fully vaccinated after 10 years by country for EPI (6–12 weeks) immunisation schedule. Cumulative total all malaria deaths averted per 100,000 fully vaccinated by country, cumulative at 10 years post introduction immunising via EPI routine immunisation schedule of 6–12 weeks (vaccination coverage is at DTP3 levels of country immunisation)

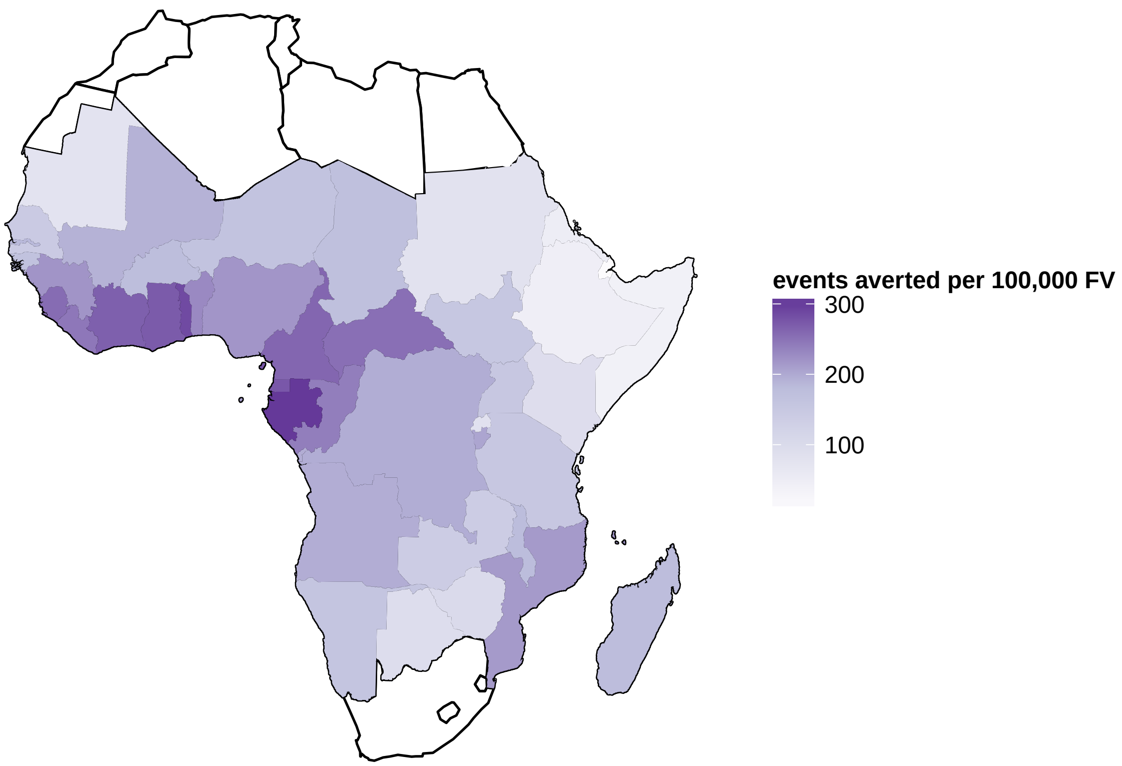

Additional file 2: Figure S22. Mean predicted total direct malaria deaths averted per 100,000 fully vaccinated after 10 years by country for expanded routine (6-9 month) immunisation schedule. Cumulative total direct malaria deaths averted per 100,000 fully vaccinated by country, cumulative at 10 years post introduction immunising via expanded routine immunisation schedule of 6-9 months (vaccination coverage is at 75% of DTP3 levels of country immunisation).

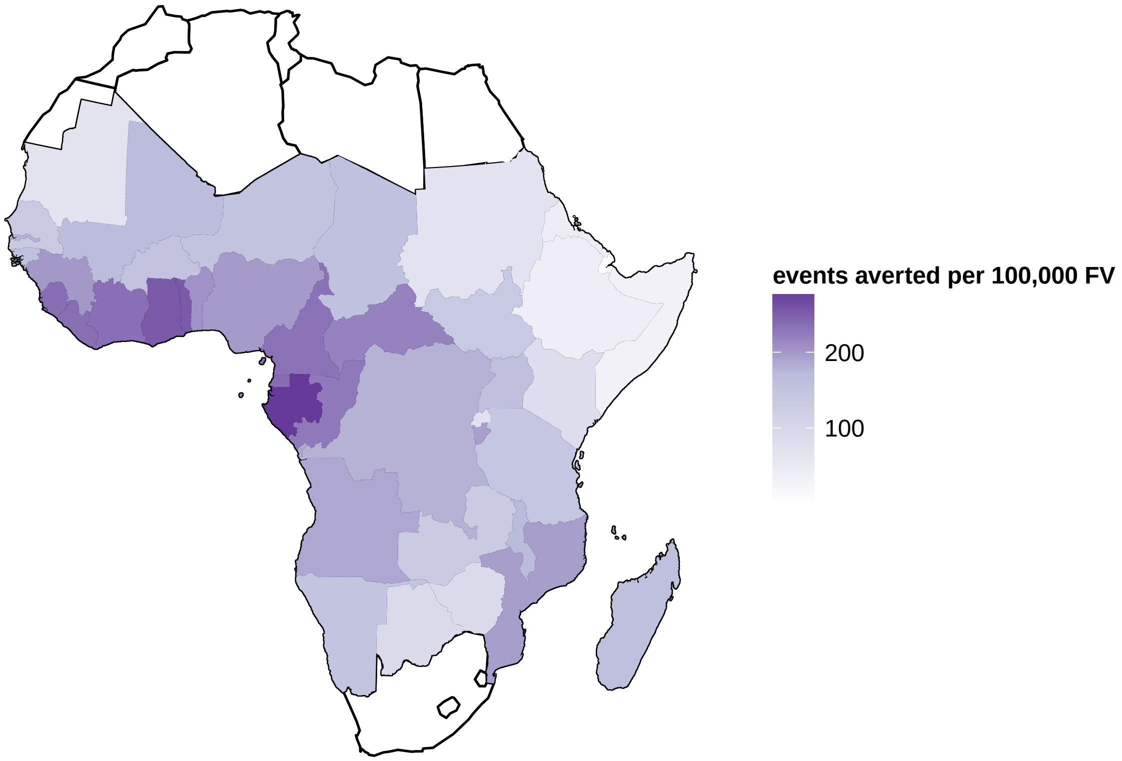

Additional file 2: Figure S23. Mean predicted total direct malaria deaths averted per 100,000 fully vaccinated after 10 years by country for EPI (6–12 weeks) immunisation schedule. Cumulative total direct malaria deaths averted per 100,000 fully vaccinated by country, cumulative at 10 years post introduction immunising via EPI routine immunisation schedule of 6–12 weeks (vaccination coverage is at DTP3 levels of country immunisation)

References

1. The RTS,S Clinical Trials Partnership (2014) (2014) Efficacy and safety of the RTS,S/AS01 malaria vaccine during 18 months after vaccination: A Phase 3 randomized, controlled trial in children and young infants at 11 African sites. PLoS Med 11: e1001685 doi:10.1371.
