# Supplementary material for: The public health impact of malaria vaccine RTS,S in malaria endemic Africa: country-specific predictions using 18 month follow-up Phase III data and simulation models
Source: BMC Med. 2015 Jul 29;13:170. doi: 10.1186/s12916-015-0408-2 (PMC4518512; doi:10.1186/s12916-015-0408-2)
Supplement: Additional file 3 — Supplementary results 2. Additional tables associated with country-specific predictions of vaccine impact. [file 12916_2015_408_MOESM3_ESM.pdf]

The public health impact of malaria vaccine RTS,S in malaria endemic Africa: country-specific predictions using 18 month follow-up Phase III data and simulation models

Melissa A Penny*[1,2], Katya Galactionova[1,2], Michael Tarantino[1,2], Marcel Tanner[1,2], Thomas A Smith[1,2]

[1] Swiss Tropical and Public Health Institute, Basel, Switzerland
[2] University of Basel, Basel, Switzerland

* Corresponding Author:
Melissa A Penny
Department of Epidemiology and Public Health
Swiss Tropical and Public Health Institute
Socinstrasse 57, 4051 Basel, Switzerland
email: melissa.penny@unibas.ch

Additional File 3: Supplementary Results 2- Tables associated with country–specific predictions of the public health impact of RTS,S

country	uncomplicated	severe	hospitalisations
Angola	5769415 (3577181-6951437)	98490 (51154-148988)	47403 (23227-73911)
Benin	2760487 (1835062-3293881)	41967 (8247-70704)	20338 (3661-33879)
Botswana	176370 (57181-265489)	3226 (1252-6098)	1532 (557-2946)
Burkina Faso	5525943 (2727596-7058000)	53816 (-33444-147239)	26838 (-18386-71094)
Burundi	1714459 (1234846-2003572)	38052 (30253-51188)	18099 (14250-25617)
Cameroon	5573456 (3852291-6569889)	88757 (21037-144609)	42924 (9890-69693)
Central African Republic	622972 (455808-727421)	10249 (1963-16813)	4955 (1022-8219)
Chad	1294503 (1047692-1430969)	27211 (15433-35322)	12992 (7267-18036)
Comoros	193723 (132832-227479)	3448 (1719-4966)	1657 (768-2521)
Congo Democratic Republic	14498191 (10482594-16738302)	258365 (107341-368634)	124216 (50082-186294)
Congo	1179838 (801561-1393760)	20694 (10409-30149)	9954 (4757-15167)
Coted Ivoire	6503296 (4351552-7788697)	94469 (7200-170510)	45908 (3393-82049)
Djibouti	6831 (3532-9003)	186 (89-259)	88 (38-129)
Equatorial Guinea	81225 (55953-97250)	1176 (24-2167)	572 (17-1050)
Eritrea	256165 (202123-305701)	7671 (5392-10051)	3607 (2374-5175)
Ethiopia	1882396 (1644570-2108092)	55797 (44627-69579)	26264 (20088-35562)
Gabon	434867 (281216-521726)	6712 (2046-10634)	3252 (859-5225)
The Gambia	426458 (323916-509571)	10321 (8261-13441)	4892 (3882-6696)
Ghana	7518203 (5083733-8870617)	130937 (62647-190602)	63010 (28137-96574)
Guinea	1807064 (1373759-2049431)	34807 (17577-47340)	16680 (8304-24158)
Guinea Bissau	241970 (205346-279814)	6332 (5380-7734)	2996 (2519-3864)
Kenya	4100748 (3178275-4859818)	97030 (80438-123159)	46051 (38241-62256)
Liberia	1289261 (892152-1521788)	20883 (8205-31182)	10092 (3766-15498)
Madagascar	4584185 (3399036-5266431)	81847 (30608-120173)	39321 (14016-58648)
Malawi	5592935 (3826948-6601774)	94493 (40609-138241)	45524 (18124-70149)
Mali	4161758 (2746785-4998651)	60254 (5610-108270)	29270 (2349-52009)
Mauritania	221302 (195559-248163)	5581 (4515-6509)	2643 (2054-3302)
Mozambique	5774429 (3663123-6923206)	91025 (30154-141352)	44019 (12516-70677)
Namibia	268622 (201162-311212)	5946 (4582-7887)	2830 (2136-3970)
Niger	4075102 (3066858-4622637)	79244 (44642-107626)	37936 (20919-54653)
Nigeria	23372156 (15902705-27681328)	373201 (106512-589250)	180411 (48083-285012)
Rwanda	1102625 (724541-1403367)	29555 (18357-38897)	13950 (8223-19359)
Sao Tome Principe	50287 (27973-64743)	839 (583-1417)	402 (249-694)
Senegal	2042086 (1638933-2435340)	52722 (42359-64749)	24945 (19701-32410)
SierraLeone	1722666 (1176070-2027701)	28511 (10745-42290)	13763 (4889-21330)
Somalia	210122 (191713-234558)	6350 (5238-7514)	3011 (2377-3728)
Sudan North	2670912 (2402333-2907909)	65273 (48624-75588)	30934 (21897-38534)
Sudan South	846250 (737821-920416)	17934 (8919-23287)	8614 (4142-11302)
Tanzania	11926406 (8209080-13980381)	237765 (164073-334880)	113555 (76590-167729)
Togo	1573846 (1143858-1831348)	26795 (7535-41829)	12915 (3647-20355)
Uganda	10686701 (5127695-13451417)	150167 (63131-264121)	73269 (26194-131838)
Zambia	3266279 (2065913-3994017)	62617 (43335-93774)	29928 (20407-46040)
Zimbabwe	984940 (817953-1177970)	27359 (21669-34382)	12913 (9883-17442)

Additional file 3: Table P1. Cumulative total events (uncomplicated, severe, hospitalisations) averted (all ages) via EPI (6-12 weeks) immunisation schedule after 10 years.

country	direct deaths	all deaths	direct DALYs	DALYs
Angola	13690 (8009-21373)	37458 (18985-45595)	767275 (466916-1184815)	2018042 (1037787-2445405)
Benin	6553 (1376-10478)	20029 (10419-28387)	368735 (96754-581570)	1079388 (570258-1515906)
Botswana	387 (145-736)	885 (277-1384)	21588 (8348-40912)	47660 (14982-74086)
Burkina Faso	10988 (-2806-23550)	41884 (16696-67077)	625227 (-106108-1311293)	2258745 (922672-3584955)
Burundi	4744 (3512-6947)	11099 (6327-13917)	264309 (195988-381330)	597740 (344369-748069)
Cameroon	13624 (2983-21573)	40926 (21886-57700)	765913 (207811-1208860)	2205423 (1197053-3081709)
Central African Republic	1583 (280-2590)	4774 (2603-6834)	89010 (20628-144717)	257294 (142292-365350)
Chad	3648 (1696-5304)	9430 (5797-12142)	203869 (103293-295187)	507998 (316263-648550)
Comoros	485 (228-745)	1334 (722-1693)	27167 (13787-41183)	71862 (39423-90335)
Congo Democratic Republic	37193 (13378-56755)	104496 (58371-139822)	2085256 (845043-3136449)	5630199 (3188971-7466102)
Congo	2886 (1541-4428)	7841 (4290-9778)	161655 (91395-245073)	422393 (234200-521478)
Coted Ivoire	15303 (1620-25164)	48363 (25104-70375)	862310 (142428-1411674)	2606672 (1374176-3759366)
Djibouti	21 (8-33)	44 (21-62)	1173 (458-1779)	2351 (1128-3318)
Equatorial Guinea	193 (8-327)	618 (325-910)	10902 (1167-18345)	33295 (17803-48615)
Eritrea	844 (496-1180)	1765 (1287-2451)	46986 (27741-65470)	95204 (68952-130203)
Ethiopia	6253 (4278-8292)	13235 (10121-16903)	347795 (238543-456214)	713363 (541156-911912)
Gabon	1010 (353-1593)	2997 (1561-4108)	56768 (22113-88548)	161508 (85410-219248)
The Gambia	1248 (878-1789)	2773 (1640-3517)	69416 (48697-97782)	149298 (89191-189202)
Ghana	18506 (8869-28479)	51121 (27536-65323)	1037050 (533734-1576241)	2754010 (1504374-3485018)
Guinea	4827 (2095-7188)	12959 (7611-16838)	270117 (128612-396319)	698125 (415352-898960)
Guinea Bissau	754 (563-1029)	1657 (1070-2060)	41899 (31266-55897)	89225 (58257-110318)
Kenya	11848 (8915-16896)	27624 (16547-35063)	660719 (496575-922573)	1488625 (901338-1887970)
Liberia	3003 (1486-4610)	8441 (4794-11061)	168466 (88317-255883)	454710 (261594-589712)
Madagascar	11903 (3386-18514)	34073 (18983-46698)	668019 (224817-1032934)	1836269 (1037844-2495317)
Malawi	13601 (5990-21096)	38177 (20877-49599)	762790 (364934-1167862)	2056891 (1140322-2646191)
Mali	9741 (1199-15949)	30741 (15769-44604)	548921 (99920-888396)	1656936 (863564-2382576)
Mauritania	674 (464-906)	1563 (1045-1952)	37570 (26843-49237)	84194 (57022-105067)
Mozambique	13660 (4528-21670)	40530 (20375-55185)	767774 (288675-1202648)	2184081 (1115992-2946041)
Namibia	749 (541-1088)	1793 (1052-2193)	41787 (31457-59661)	96559 (57283-118032)
Niger	10813 (5562-16079)	28332 (16716-35335)	604643 (334069-884760)	1526183 (911607-1885554)
Nigeria	56390 (16213-88724)	166835 (89295-230306)	3168616 (1061306-4919068)	8989980 (4882893-12295519)
Rwanda	3393 (1796-4841)	7189 (4100-9736)	188750 (100040-263378)	387450 (222998-523322)
Sao Tome Principe	104 (68-173)	257 (127-351)	5841 (3791-9589)	13814 (6879-18748)
Senegal	6254 (4397-8618)	13768 (8629-17524)	347838 (244383-468826)	741556 (469845-938873)
SierraLeone	4173 (1626-6441)	11950 (6498-16000)	234151 (100561-357072)	643800 (355071-853806)
Somalia	730 (559-905)	1540 (1215-1941)	40439 (30728-50391)	82815 (66205-104540)
Sudan North	8015 (4899-10785)	19120 (12899-23961)	447210 (291714-589252)	1030449 (704158-1288898)
Sudan South	2437 (1310-3429)	6416 (4049-8470)	136273 (77288-190146)	345692 (220706-453522)
Tanzania	30968 (21584-47072)	77284 (42663-94889)	1729963 (1240941-2592490)	4163244 (2324660-5097922)
Togo	4022 (879-6453)	11811 (6510-16589)	225896 (61463-360415)	636453 (355875-886276)
Uganda	21399 (9672-34981)	63299 (27935-86446)	1203515 (580564-1942708)	3410240 (1546369-4636575)
Zambia	8129 (5671-12846)	20233 (10510-25452)	454263 (320266-709167)	1089926 (572450-1364822)
Zimbabwe	3142 (2148-4102)	6809 (4583-9079)	174857 (119687-227565)	366925 (249839-484132)

Additional file 3: Table P2. Cumulative total events averted (deaths and DALYs) via EPI (6-12 weeks) immunisation schedule after 10 years. Events include direct DALYs which are DALYs calculated from direct malaria deaths rather than all deaths.

country	uncomplicated	severe	hospitalisations
Angola	8352964 (5146713-10005777)	148960 (104354-218074)	71551 (49869-106487)
Benin	4006184 (2631675-4759999)	68145 (33108-100689)	32808 (16900-50240)
Botswana	276213 (91201-411346)	4987 (1851-9024)	2385 (844-4389)
Burkina Faso	8065424 (3979542-10254814)	99791 (3159-198091)	48542 (2265-94813)
Burundi	2522266 (1815161-2928760)	57267 (45194-75533)	27322 (21356-36771)
Cameroon	8086042 (5517259-9493723)	143840 (74809-207548)	69176 (38069-103354)
Central African Republic	906273 (654195-1054102)	16978 (8671-23982)	8159 (4383-11917)
Chad	1891218 (1516205-2081332)	42370 (31793-54334)	20239 (15228-26546)
Comoros	281676 (191231-329076)	5342 (3642-7528)	2562 (1765-3701)
Congo Democratic Republic	21172818 (15128766-24349309)	410092 (261698-566322)	196661 (129591-279782)
Congo	1704602 (1146354-2003584)	31412 (21239-44881)	15084 (10292-22081)
Coted Ivoire	9435714 (6233716-11253879)	156968 (66070-235235)	75646 (34530-117859)
Djibouti	11306 (5859-14800)	311 (143-429)	147 (65-210)
Equatorial Guinea	118035 (80222-140666)	1990 (799-2970)	959 (422-1491)
Eritrea	400732 (321439-471057)	12190 (8436-15501)	5758 (3857-7643)
Ethiopia	2910729 (2562121-3240818)	87701 (67976-106324)	41455 (31635-51853)
Gabon	628434 (401600-750925)	10488 (5516-15667)	5053 (2764-7770)
The Gambia	619008 (471075-733154)	15276 (11506-19570)	7273 (5414-9508)
Ghana	10873668 (7278972-12767407)	201038 (132766-286969)	96527 (64755-141315)
Guinea	2625668 (1976099-2965468)	54226 (38037-72635)	25962 (18482-35717)
Guinea Bissau	353261 (300461-404813)	9451 (7590-11453)	4492 (3598-5563)
Kenya	6186137 (4781685-7269452)	150638 (119390-189757)	71688 (56466-92307)
Liberia	1859506 (1269698-2185483)	31624 (18199-46113)	15232 (9150-22816)
Madagascar	6785631 (4957933-7774698)	133845 (83537-182203)	64113 (41008-89983)
Malawi	8113418 (5488021-9529073)	146451 (91191-210129)	70358 (45011-103890)
Mali	6061046 (3952782-7248290)	100221 (43115-150282)	48288 (22290-75286)
Mauritania	342215 (305340-381133)	8907 (7758-10468)	4231 (3656-5078)
Mozambique	8376375 (5266502-9996988)	144135 (81142-212707)	69337 (39989-105280)
Namibia	407811 (304402-470008)	9271 (7622-12088)	4424 (3628-5893)
Niger	5937908 (4429816-6704386)	122260 (90255-163670)	58525 (43302-80418)
Nigeria	33869041 (22753259-39941274)	594676 (319953-864699)	286023 (161763-429867)
Rwanda	1678527 (1121284-2110231)	45702 (27532-60037)	21677 (12836-29306)
Sao Tome Principe	72201 (40412-92016)	1186 (787-1905)	570 (351-926)
Senegal	3001542 (2441627-3538093)	79224 (60520-96714)	37645 (28643-46941)
SierraLeone	2490108 (1678854-2918997)	44356 (26029-63955)	21330 (13105-31668)
Somalia	319122 (293668-351685)	9757 (7980-11629)	4623 (3687-5665)
Sudan North	4143186 (3756007-4486189)	105451 (90290-123910)	50089 (43150-60181)
Sudan South	1280774 (1093515-1395845)	29217 (20198-37447)	13975 (9606-18108)
Tanzania	17378192 (11918115-20217504)	358291 (283686-492387)	171352 (132415-239575)
Togo	2287138 (1640815-2650330)	43535 (24398-61031)	20896 (12290-30301)
Uganda	15515888 (7444158-19429402)	214312 (121837-358983)	103846 (55812-174170)
Zambia	4728905 (2983205-5736227)	92979 (67981-134812)	44511 (31655-65694)
Zimbabwe	1487357 (1251525-1752816)	42175 (32122-52163)	19987 (15095-25415)

Additional file 3: Table P3. Cumulative total events (uncomplicated, severe, hospitalisations) averted (all ages) via EPI with booster immunisation schedule after 10 years.

country	direct deaths	all deaths	direct DALYs	DALYs
Angola	19792 (12777-29905)	47795 (25728-56277)	1103163 (717020-1653706)	2572196 (1396487-3041597)
Benin	9863 (5105-15400)	25580 (14394-32995)	550906 (295812-852936)	1377317 (781419-1761285)
Botswana	583 (201-1033)	1221 (378-1817)	32402 (11222-57410)	65721 (20485-98328)
Burkina Faso	17500 (3763-31290)	53021 (22948-77314)	984112 (245408-1726789)	2857324 (1254471-4129957)
Burundi	6865 (4799-9748)	14616 (8968-17988)	381000 (264107-535952)	786190 (483343-967508)
Cameroon	20498 (10992-31803)	52401 (30310-66978)	1144165 (634777-1760305)	2821283 (1632424-3575751)
Central African Republic	2414 (1289-3773)	6146 (3640-7900)	134735 (74541-208623)	330956 (195897-422209)
Chad	5396 (3939-7614)	12308 (8095-14612)	299989 (222807-419235)	662392 (434442-784467)
Comoros	713 (490-1060)	1716 (1000-2030)	39697 (27838-58532)	92358 (54271-109398)
Congo Democratic Republic	55540 (35275-82217)	135221 (81503-164321)	3095015 (2013700-4554444)	7278850 (4384932-8771828)
Congo	4174 (2939-6244)	10009 (5840-11740)	232536 (164471-344768)	538590 (316604-634963)
Coted Ivoire	23301 (10740-37435)	61738 (34734-81350)	1302481 (628416-2071198)	3324678 (1872455-4343431)
Djibouti	34 (14-49)	65 (32-94)	1890 (764-2709)	3513 (1716-5047)
Equatorial Guinea	297 (129-484)	791 (449-1047)	16625 (7613-26768)	42583 (24162-55941)
Eritrea	1310 (806-1810)	2523 (1862-3478)	72569 (45274-99231)	135845 (101559-185379)
Ethiopia	9568 (6729-12572)	18651 (14643-23600)	529964 (377553-689733)	1003781 (799136-1270607)
Gabon	1487 (856-2278)	3807 (2115-4821)	83002 (49152-126120)	204907 (114809-257163)
The Gambia	1776 (1234-2462)	3624 (2304-4639)	98439 (68228-135043)	194926 (123687-249638)
Ghana	26971 (18371-40366)	65328 (37735-77186)	1502893 (1043067-2229255)	3515804 (2046599-4171657)
Guinea	7116 (4924-10309)	16746 (10550-19774)	396021 (279463-567486)	901250 (567095-1061502)
Guinea Bissau	1084 (808-1430)	2188 (1507-2733)	60037 (44889-78170)	117703 (81343-147236)
Kenya	17750 (13066-24185)	37464 (23616-47406)	985198 (715788-1326721)	2016060 (1280732-2536483)
Liberia	4333 (2824-6529)	10712 (6429-13100)	241672 (160423-360916)	576463 (348240-698043)
Madagascar	18224 (11047-27365)	44738 (26918-55185)	1016071 (633432-1513202)	2408909 (1446881-2947788)
Malawi	19944 (13002-30010)	48858 (28643-58541)	1111948 (740725-1657652)	2629720 (1552898-3122457)
Mali	14865 (6971-23741)	39354 (21911-51764)	831035 (407768-1313978)	2119364 (1185269-2763738)
Mauritania	1039 (828-1346)	2178 (1526-2691)	57641 (46356-73595)	117212 (82851-144484)
Mozambique	20302 (11887-31040)	51733 (28022-64774)	1133420 (682852-1717538)	2785159 (1522456-3456871)
Namibia	1124 (820-1584)	2433 (1533-2961)	62424 (45058-87027)	130904 (82737-159322)
Niger	15843 (11678-22914)	36699 (23221-43107)	881480 (660773-1261253)	1974899 (1248591-2314220)
Nigeria	83994 (47446-128206)	213086 (123000-268507)	4687761 (2729914-7106286)	11471635 (6673020-14329592)
Rwanda	5080 (2752-6947)	9921 (5903-13761)	281368 (153931-381725)	533813 (320040-736448)
Sao Tome Principe	144 (85-230)	324 (162-422)	8015 (4704-12699)	17424 (8768-22725)
Senegal	9061 (6382-12004)	18298 (12161-23490)	502090 (356605-656917)	984313 (659094-1263683)
SierraLeone	6126 (3799-9212)	15237 (8879-18776)	341630 (216931-509135)	820103 (481459-1001549)
Somalia	1086 (823-1351)	2112 (1795-2593)	59991 (45569-74581)	113493 (97342-139727)
Sudan North	12488 (9343-16309)	26711 (18684-32716)	693373 (530604-892429)	1437770 (1015190-1756424)
Sudan South	3760 (2684-5143)	8654 (5989-10297)	209022 (151793-284225)	465772 (323697-551369)
Tanzania	44732 (31119-65615)	100179 (59252-120301)	2487107 (1737253-3615410)	5390383 (3210904-6457823)
Togo	6073 (3371-9372)	15205 (9003-19205)	338698 (194107-518242)	818625 (484285-1025623)
Uganda	30079 (15583-46951)	79469 (34944-107876)	1682856 (868406-2594935)	4276464 (1890674-5769869)
Zambia	11597 (7518-17661)	25993 (14290-31514)	645042 (420396-974559)	1398598 (774554-1692478)
Zimbabwe	4699 (3264-6162)	9343 (6477-12403)	260397 (182826-338437)	502750 (352081-662350)

Additional file 3: Table P4. Cumulative total events averted (deaths and DALYs) via EPI with booster immunisation schedule after 10 years. Events include direct DALYs which are DALYs calculated from direct malaria deaths rather than all deaths.

country	uncomplicated	severe	hospitalisations
Angola	4634476 (2841787-5593410)	91708 (66321-130143)	43952 (32565-63960)
Benin	2188371 (1439850-2622555)	44959 (28911-62361)	21536 (13384-31377)
Botswana	145364 (46915-217348)	2664 (914-4729)	1268 (415-2256)
Burkina Faso	4286288 (2109527-5526543)	74117 (28595-114948)	35668 (11606-60099)
Burundi	1404804 (1001666-1625662)	33631 (24346-44271)	16021 (11149-22168)
Cameroon	4424719 (3025938-5236176)	94631 (63278-128411)	45277 (29290-64764)
Central African Republic	495502 (360618-580913)	11357 (7529-15160)	5426 (3460-7624)
Chad	1047448 (839559-1157099)	26282 (21591-32821)	12514 (10214-16654)
Comoros	155479 (105335-182881)	3341 (2603-4530)	1598 (1234-2269)
Congo Democratic Republic	11607704 (8301998-13425745)	260633 (194858-343959)	124503 (91549-173659)
Congo	947494 (634992-1121693)	19431 (14983-26876)	9309 (7165-13293)
Coted Ivoire	5134150 (3402625-6182886)	105857 (63972-147325)	50700 (29223-74555)
Djibouti	5721 (2994-7475)	163 (69-228)	77 (32-113)
Equatorial Guinea	64071 (43755-77141)	1358 (805-1876)	650 (365-949)
Eritrea	215066 (171818-254736)	6781 (4324-8885)	3196 (1947-4319)
Ethiopia	1574544 (1395817-1767233)	49355 (35705-61851)	23298 (16222-30064)
Gabon	345433 (220510-416348)	6729 (4479-9562)	3229 (2104-4781)
The Gambia	352385 (265186-416391)	9009 (6206-11560)	4284 (2839-5808)
Ghana	6027118 (4024078-7127490)	125788 (95881-172920)	60228 (45522-86016)
Guinea	1454242 (1093386-1650880)	34026 (26915-43977)	16237 (12730-22234)
Guinea Bissau	200359 (169191-229046)	5582 (4157-6783)	2648 (1905-3398)
Kenya	3369802 (2586114-3963083)	87434 (61790-109521)	41510 (28390-54875)
Liberia	1030190 (701588-1220724)	19596 (13520-27598)	9416 (6515-13686)
Madagascar	3668772 (2700566-4217859)	85659 (63216-111500)	40840 (29390-56432)
Malawi	4478154 (3021417-5299574)	91957 (67256-126738)	44049 (31978-62949)
Mali	3289186 (2151970-3969945)	67262 (40962-93825)	32215 (18754-47476)
Mauritania	182238 (163130-202391)	5095 (4012-6167)	2414 (1843-2979)
Mozambique	4594014 (2889125-5527098)	93082 (64845-130411)	44594 (30250-65069)
Namibia	219426 (162617-251793)	5350 (4080-6908)	2547 (1863-3476)
Niger	3291460 (2446379-3735140)	75377 (61661-97960)	35990 (29429-49339)
Nigeria	18590070 (12491423-22101239)	385713 (260921-530848)	184719 (121949-265437)
Rwanda	920494 (611165-1160633)	25887 (14187-34750)	12249 (6642-16659)
Sao Tome Principe	41088 (22749-52655)	681 (423-1059)	327 (193-524)
Senegal	1692122 (1375154-1996742)	46520 (32622-57857)	22062 (14976-28461)
SierraLeone	1373993 (924726-1623164)	28190 (19996-38827)	13510 (9458-19301)
Somalia	176533 (162392-195960)	5607 (4330-6857)	2663 (1938-3238)
Sudan North	2192332 (1996901-2366395)	60762 (49485-72500)	28785 (22644-35089)
Sudan South	688675 (599325-754716)	18011 (13892-22409)	8597 (6632-10966)
Tanzania	9698677 (6599986-11359883)	215134 (165375-292010)	102701 (76072-145881)
Togo	1253275 (902726-1462705)	28574 (19855-37963)	13644 (9176-19241)
Uganda	8486734 (4007514-10798534)	126997 (73061-200180)	61501 (37186-100211)
Zambia	2657856 (1661738-3247443)	55570 (39079-79368)	26562 (18218-39283)
Zimbabwe	819696 (689881-971513)	24246 (17000-31153)	11466 (7889-15061)

Additional file 3: Table P5. Cumulative total events (uncomplicated, severe, hospitalisations) averted (all ages) via Extended routine (6-9 months) immunisation schedule after 10 years.

country	direct deaths	all deaths	direct DALYs	DALYs
Angola	10944 (6886-17670)	24414 (13266-30567)	612058 (378135-973863)	1316047 (721270-1655462)
Benin	5606 (2746-9246)	13186 (7127-17377)	314096 (163253-508008)	711128 (386576-940618)
Botswana	294 (86-544)	581 (173-877)	16402 (4930-30183)	31311 (9418-47458)
Burkina Faso	10064 (1973-18363)	26960 (12750-38371)	568042 (139960-1011478)	1455814 (689790-2051991)
Burundi	3776 (2376-5640)	7528 (4253-9627)	210043 (133766-309656)	405471 (231523-519527)
Cameroon	11686 (5839-19003)	27103 (14777-35554)	654130 (346829-1043119)	1461488 (801538-1924707)
Central African Republic	1394 (698-2236)	3210 (1807-4148)	78008 (41687-122789)	173104 (97897-224706)
Chad	3048 (2135-4566)	6419 (3846-7831)	169855 (122354-249777)	345876 (208979-423823)
Comoros	399 (268-632)	883 (493-1114)	22282 (15053-34753)	47596 (26771-60300)
Congo Democratic Republic	31335 (19053-49353)	69789 (39254-88697)	1750723 (1106733-2707277)	3762174 (2131214-4801580)
Congo	2324 (1560-3701)	5144 (2904-6470)	129844 (87751-203676)	277215 (157865-350264)
Coted Ivoire	13346 (5612-22292)	31881 (17060-42691)	748187 (341569-1224003)	1719638 (924720-2311213)
Djibouti	17 (6-26)	31 (14-47)	951 (336-1417)	1673 (764-2496)
Equatorial Guinea	171 (66-288)	410 (219-553)	9613 (4095-15778)	22106 (11872-29921)
Eritrea	698 (377-1003)	1271 (883-1906)	38744 (21030-55267)	68507 (47166-101067)
Ethiopia	5150 (3297-7715)	9448 (6928-12642)	285711 (183450-409728)	509062 (369160-670896)
Gabon	832 (473-1367)	1946 (1075-2535)	46600 (27481-75237)	104920 (58370-137132)
The Gambia	993 (616-1444)	1911 (1095-2494)	55152 (34066-79181)	102885 (59675-134670)
Ghana	15094 (10130-24065)	33637 (18755-42598)	843547 (568787-1323560)	1812993 (1019086-2306007)
Guinea	4024 (2676-6191)	8712 (5055-10870)	224502 (154004-339369)	469502 (274603-588357)
Guinea Bissau	607 (409-843)	1155 (719-1483)	33681 (22627-46090)	62197 (39207-79195)
Kenya	9641 (6290-13918)	19039 (11435-24901)	536450 (350676-760724)	1026013 (621814-1328948)
Liberia	2394 (1573-3863)	5460 (3199-6921)	133961 (89786-212782)	294316 (173909-374398)
Madagascar	10270 (5889-16209)	22957 (12902-29179)	574070 (345635-887685)	1237922 (700439-1580238)
Malawi	11150 (7234-17900)	25107 (14131-31913)	623550 (415600-984593)	1353507 (767805-1727409)
Mali	8481 (3702-14186)	20261 (10826-27000)	475564 (224126-779185)	1092972 (586895-1461778)
Mauritania	556 (394-760)	1088 (728-1381)	30904 (21984-41404)	58626 (39615-73545)
Mozambique	11430 (6584-18712)	26547 (14256-34431)	640092 (383452-1029308)	1431538 (773857-1863765)
Namibia	603 (398-895)	1217 (709-1514)	33556 (22360-49081)	65542 (38572-81800)
Niger	8879 (6250-13622)	19018 (11073-23389)	495279 (350116-747375)	1024836 (601898-1266188)
Nigeria	47545 (25765-77356)	109882 (60637-143111)	2661370 (1511749-4250696)	5924948 (3290493-7745688)
Rwanda	2748 (1311-3896)	5081 (2838-7382)	152590 (72996-215188)	273766 (154932-394054)
Sao Tome Principe	77 (38-133)	164 (81-223)	4304 (2150-7345)	8824 (4403-12010)
Senegal	5044 (3193-7036)	9603 (5890-12771)	280124 (176891-384604)	517243 (320904-681813)
SierraLeone	3440 (2080-5523)	7836 (4390-10070)	192395 (120307-303661)	422386 (238378-544927)
Somalia	595 (412-784)	1099 (911-1355)	32930 (23086-42901)	59134 (48970-73207)
Sudan North	6686 (4818-9186)	13293 (9008-16381)	372030 (270046-500766)	716464 (490745-882933)
Sudan South	2088 (1579-2942)	4430 (3079-5225)	116378 (90719-162328)	238719 (166732-280680)
Tanzania	24772 (15767-38533)	51678 (28893-63473)	1381257 (887950-2118386)	2784769 (1572254-3427670)
Togo	3481 (1774-5588)	7912 (4352-10314)	194622 (105343-306225)	426610 (236101-558452)
Uganda	15632 (4995-26425)	38577 (12618-53008)	879543 (292259-1468715)	2080384 (691138-2835765)
Zambia	6405 (3808-10335)	13406 (7159-16852)	357392 (215151-569499)	722486 (389801-908663)
Zimbabwe	2569 (1617-3512)	4813 (3240-6787)	142627 (89954-194020)	259347 (176503-361042)

Additional file 3: Table P6. Cumulative total events averted (deaths and DALYs) via Extended routine (6-9 months) immunisation schedule after 10 years. Events include direct DALYs which are DALYs calculated from direct malaria deaths rather than all deaths.

country	uncomplicated	severe	hospitalisations
Angola	6897229 (4268039-8270251)	132302 (96798-183520)	63278 (45292-90983)
Benin	3293316 (2180416-3911770)	65873 (50845-90271)	31451 (23844-45486)
Botswana	225882 (74437-336029)	4012 (1376-6810)	1917 (649-3454)
Burkina Faso	6576317 (3288540-8359005)	110631 (63531-168334)	52995 (28273-86654)
Burundi	2093480 (1507086-2415764)	49019 (34091-63244)	23339 (16064-31051)
Cameroon	6649579 (4572740-7804473)	138614 (109749-186412)	66119 (51478-93673)
Central African Republic	746930 (543982-868828)	16720 (13128-22216)	7963 (6074-10989)
Chad	1566859 (1259421-1725017)	38446 (33462-47589)	18274 (15565-23392)
Comoros	232459 (158667-271725)	4861 (3888-6495)	2320 (1800-3226)
Congo Democratic Republic	17451952 (12542084-20066002)	382101 (323416-498867)	182080 (148675-248401)
Congo	1407468 (950255-1656152)	28038 (22254-38041)	13399 (10281-18909)
Coted Ivoire	7744033 (5159522-9232029)	155665 (115481-214524)	74291 (53778-108455)
Djibouti	9231 (4839-11994)	258 (106-362)	122 (49-172)
Equatorial Guinea	96888 (66455-115370)	2006 (1475-2748)	956 (683-1389)
Eritrea	333128 (267899-388789)	10339 (6452-13503)	4877 (2965-6509)
Ethiopia	2419848 (2139440-2674228)	74530 (52563-92865)	35187 (24109-44962)
Gabon	516406 (331715-617533)	9749 (7581-13576)	4664 (3590-6832)
The Gambia	518179 (393930-610219)	13019 (8607-16405)	6190 (4041-8058)
Ghana	8973448 (6037768-10544759)	182055 (144712-245966)	86957 (66963-122438)
Guinea	2171183 (1639414-2453027)	49605 (42859-63366)	23620 (19722-31357)
Guinea Bissau	295807 (251359-336760)	8100 (5781-9789)	3843 (2699-4738)
Kenya	5121202 (3962689-5979922)	130129 (88555-163299)	61776 (41301-78352)
Liberia	1531080 (1047436-1802164)	28192 (22087-38818)	13501 (10583-19443)
Madagascar	5584156 (4117140-6387247)	127254 (108158-163943)	60538 (50242-81338)
Malawi	6693900 (4548123-7867404)	133657 (108189-181143)	63847 (49899-90423)
Mali	4976202 (3274294-5946509)	99161 (74204-136913)	47330 (34541-69167)
Mauritania	282040 (252899-311461)	7715 (5831-9339)	3655 (2695-4479)
Mozambique	6892340 (4367744-8226430)	135639 (105133-186924)	64805 (49140-93679)
Namibia	335405 (250561-383813)	7976 (5821-10128)	3796 (2735-4978)
Niger	4916384 (3674017-5554781)	109933 (93591-140871)	52378 (42813-69624)
Nigeria	27876000 (18851159-32877242)	562945 (448753-764371)	268766 (211628-383996)
Rwanda	1398898 (938079-1748370)	38737 (20670-52155)	18357 (9626-24835)
Sao Tome Principe	60088 (33682-76598)	959 (620-1454)	460 (280-738)
Senegal	2511406 (2049716-2943032)	67881 (45759-84085)	32203 (21350-40270)
SierraLeone	2050035 (1389113-2404936)	40875 (33363-55345)	19530 (15384-27744)
Somalia	267606 (247161-293744)	8325 (6154-10209)	3938 (2845-4909)
Sudan North	3410550 (3122251-3657796)	92496 (72401-110555)	43797 (33299-53261)
Sudan South	1059313 (910080-1157975)	26992 (23055-33755)	12837 (10827-16169)
Tanzania	14422295 (9908764-16802783)	312192 (235048-414563)	148860 (110170-203263)
Togo	1883883 (1362010-2181854)	41944 (34105-55152)	19974 (15846-27553)
Uganda	12709604 (6116324-16021613)	178618 (112275-271922)	86304 (52247-137402)
Zambia	3930169 (2482909-4774229)	80089 (56388-111653)	38244 (26231-54528)
Zimbabwe	1239629 (1046512-1452202)	36052 (24506-46041)	17056 (11336-22132)

Additional file 3: Table P7. Cumulative total events (uncomplicated, severe, hospitalisations) averted (all ages) via Extended routine with booster immunisation schedule after 10 years.

country	direct deaths	all deaths	direct DALYs	DALYs
Angola	15483 (10535-23505)	32109 (19014-38907)	861397 (590291-1285013)	1727708 (1026524-2096128)
Benin	8078 (5807-12365)	17409 (10655-21598)	449625 (324803-674906)	936948 (576643-1168208)
Botswana	427 (138-724)	804 (260-1211)	23729 (7584-40878)	43295 (14048-64908)
Burkina Faso	14834 (8201-24644)	35432 (17895-47384)	829271 (469335-1347262)	1908842 (954694-2536579)
Burundi	5365 (3524-7602)	10122 (6302-12715)	297301 (194554-414871)	544203 (341720-683793)
Cameroon	16835 (12303-25417)	35850 (22212-44160)	936395 (687583-1386692)	1929194 (1202359-2388876)
Central African Republic	2018 (1529-2993)	4261 (2717-5184)	112180 (85180-163464)	229277 (147011-280605)
Chad	4367 (3401-6161)	8596 (5824-10199)	242174 (191146-335095)	462325 (315150-549020)
Comoros	569 (409-847)	1170 (729-1402)	31636 (22883-46246)	62944 (39417-75868)
Congo Democratic Republic	45088 (33448-66437)	92964 (59114-111245)	2505146 (1868562-3621759)	5001597 (3198887-6019213)
Congo	3288 (2373-4923)	6771 (4233-8135)	182817 (132868-268888)	364261 (228950-439977)
Coted Ivoire	19320 (13669-29777)	42106 (25703-52854)	1075619 (769165-1625129)	2266383 (1391405-2859164)
Djibouti	26 (9-39)	46 (22-67)	1457 (512-2132)	2455 (1174-3596)
Equatorial Guinea	249 (172-384)	543 (334-683)	13874 (9694-20950)	29205 (18068-36954)
Eritrea	1039 (574-1425)	1807 (1372-2594)	57445 (31959-78332)	97173 (74554-137973)
Ethiopia	7590 (4781-9892)	13325 (10155-17330)	419480 (265562-543679)	716362 (551120-923152)
Gabon	1187 (849-1822)	2555 (1568-3162)	66086 (47594-99537)	137490 (84797-170919)
The Gambia	1395 (841-1931)	2554 (1631-3251)	77210 (46357-105284)	137281 (88351-174924)
Ghana	21435 (15387-32093)	44339 (27522-53453)	1191668 (860969-1752454)	2385402 (1488582-2891071)
Guinea	5750 (4370-8312)	11585 (7576-13683)	319157 (244045-452815)	623163 (410053-736792)
Guinea Bissau	858 (559-1139)	1556 (1079-1956)	47456 (30866-61910)	83618 (58518-104840)
Kenya	14016 (8894-19235)	26144 (16990-33466)	776470 (497242-1045551)	1406071 (921825-1792232)
Liberia	3386 (2553-5117)	7158 (4620-8661)	188488 (143212-279509)	385123 (249991-468090)
Madagascar	14989 (11276-22034)	30938 (19879-36961)	832756 (629194-1199766)	1664773 (1075257-2000739)
Malawi	15893 (11553-23921)	33145 (20774-40006)	884041 (646740-1306027)	1783481 (1124041-2163945)
Mali	12300 (8813-19000)	26814 (16323-33559)	684905 (493452-1036974)	1443397 (883393-1815510)
Mauritania	824 (564-1075)	1526 (1124-1903)	45605 (31613-58293)	82072 (60972-101759)
Mozambique	16379 (11435-25037)	34998 (21051-43103)	911615 (639967-1367281)	1883533 (1138354-2330724)
Namibia	879 (602-1239)	1674 (1079-2050)	48706 (33609-67518)	90008 (58475-110275)
Niger	12666 (9544-18307)	25324 (16527-30002)	703196 (533264-997837)	1362150 (894458-1615091)
Nigeria	68197 (49553-103387)	145001 (90003-178077)	3794265 (2772161-5642850)	7803130 (4870926-9631307)
Rwanda	3997 (1884-5599)	7050 (4259-9846)	221095 (104267-308464)	379111 (230886-527082)
Sao Tome Principe	105 (64-165)	212 (112-283)	5870 (3581-9065)	11414 (6029-15146)
Senegal	7169 (4410-9551)	12989 (8752-16833)	396652 (243604-519117)	698223 (474633-901935)
SierraLeone	4902 (3574-7367)	10319 (6448-12557)	272658 (200042-402244)	555190 (348927-678933)
Somalia	861 (578-1115)	1517 (1294-1921)	47466 (32087-61623)	81423 (69756-103332)
Sudan North	9963 (7013-13100)	18692 (14036-22785)	551827 (393982-709838)	1005300 (761830-1224934)
Sudan South	3068 (2393-4043)	6056 (4438-7064)	170045 (134059-221880)	325681 (239709-380589)
Tanzania	35093 (24566-51504)	68735 (42426-83808)	1948121 (1372835-2812287)	3697137 (2297256-4509109)
Togo	5021 (3743-7483)	10508 (6626-12818)	279051 (208896-407807)	565407 (358743-693618)
Uganda	21775 (11262-35249)	49827 (23061-64301)	1218693 (632674-1927233)	2682882 (1251148-3436707)
Zambia	9000 (5948-13668)	17702 (10275-22120)	500015 (332665-747705)	952299 (555949-1189151)
Zimbabwe	3729 (2296-4945)	6658 (4789-9082)	206245 (127195-272080)	357933 (260027-484788)

Additional file 3: Table P8. Cumulative total events averted (deaths and DALYs) via Extended routine with booster immunisation schedule after 10 years. Events include direct DALYs which are DALYs calculated from direct malaria deaths rather than all deaths.
